# Increasing the dose intensity of chemotherapy by more frequent administration or sequential scheduling: a patient-level meta-analysis of 37 298 women with early breast cancer in 26 randomised trials

**DOI:** 10.1016/S0140-6736(18)33137-4

**Published:** 2019-04-06

**Authors:** Early Breast Cancer Trialists' Collaborative Group (EBCTCG), Richard Gray, Rosie Bradley, Jeremy Braybrooke, Zulian Liu, Richard Peto, Lucy Davies, David Dodwell, Paul McGale, Hongchao Pan, Carolyn Taylor, William Barlow, Judith Bliss, Paolo Bruzzi, David Cameron, George Fountzilas, Sibylle Loibl, John Mackey, Miguel Martin, Lucia Del Mastro, Volker Möbus, Valentina Nekljudova, Sabino De Placido, Sandra Swain, Michael Untch, Kathleen I Pritchard, Jonas Bergh, Larry Norton, Clare Boddington, Julie Burrett, Mike Clarke, Christina Davies, Fran Duane, Vaughan Evans, Lucy Gettins, Jon Godwin, Robert Hills, Sam James, Hui Liu, Elizabeth MacKinnon, Gurdeep Mannu, Theresa McHugh, Philip Morris, Simon Read, Yaochen Wang, Zhe Wang, Peter Fasching, Nadia Harbeck, Pascal Piedbois, Michael Gnant, Guenther Steger, Angelo Di Leo, Stella Dolci, Prue Francis, Denis Larsimont, Jean Marie Nogaret, Catherine Philippson, Martine Piccart, Sabine Linn, Petronella Peer, Vivianne Tjan-Heijnen, Sonja Vliek, John Mackey, Dennis Slamon, John Bartlett, Vivien H Bramwell, Bingshu Chen, Stephen Chia, Karen Gelmon, Paul Goss, Mark Levine, Wendy Parulekar, Joseph Pater, Eileen Rakovitch, Lois Shepherd, Dongsheng Tu, Tim Whelan, Don Berry, Gloria Broadwater, Constance Cirrincione, Hyman Muss, Raymond Weiss, Yi Shan, Yong Fu Shao, Xiang Wang, Binghe Xu, Dong-Bing Zhao, Harry Bartelink, Nina Bijker, Jan Bogaerts, Fatima Cardoso, Tanja Cufer, Jean-Pierre Julien, Philip Poortmans, Emiel Rutgers, Cornelis van de Velde, Eva Carrasco, Miguel Angel Segui, Jens Uwe Blohmer, Serban Costa, Bernd Gerber, Christian Jackisch, Gunter von Minckwitz, Mario Giuliano, Michele De Laurentiis, Christina Bamia, Georgia-Angeliki Koliou, Dimitris Mavroudis, Roger A'Hern, Paul Ellis, Lucy Kilburn, James Morden, John Yarnold, Mohammad Sadoon, Augustinus H Tulusan, Stewart Anderson, Gordon Bass, Joe Costantino, James Dignam, Bernard Fisher, Charles Geyer, Eleftherios P Mamounas, Soon Paik, Carol Redmond, D Lawrence Wickerham, Marco Venturini, Claudia Bighin, Simona Pastorino, Paolo Pronzato, Mario Roberto Sertoli, Theodorus Foukakis, Kathy Albain, Rodrigo Arriagada, Elizabeth Bergsten Nordström, Francesco Boccardo, Etienne Brain, Lisa Carey, Alan Coates, Robert Coleman, Candace Correa, Jack Cuzick, Nancy Davidson, Mitch Dowsett, Marianne Ewertz, John Forbes, Richard Gelber, Aron Goldhirsch, Pamela Goodwin, Daniel Hayes, Catherine Hill, James Ingle, Reshma Jagsi, Wolfgang Janni, Hirofumi Mukai, Yasuo Ohashi, Lori Pierce, Vinod Raina, Peter Ravdin, Daniel Rea, Meredith Regan, John Robertson, Joseph Sparano, Andrew Tutt, Giuseppe Viale, Nicholas Wilcken, Norman Wolmark, Wiliam Wood, Milvia Zambetti

## Abstract

**Background:**

Increasing the dose intensity of cytotoxic therapy by shortening the intervals between cycles, or by giving individual drugs sequentially at full dose rather than in lower-dose concurrent treatment schedules, might enhance efficacy.

**Methods:**

To clarify the relative benefits and risks of dose-intense and standard-schedule chemotherapy in early breast cancer, we did an individual patient-level meta-analysis of trials comparing 2-weekly versus standard 3-weekly schedules, and of trials comparing sequential versus concurrent administration of anthracycline and taxane chemotherapy. The primary outcomes were recurrence and breast cancer mortality. Standard intention-to-treat log-rank analyses, stratified by age, nodal status, and trial, yielded dose-intense versus standard-schedule first-event rate ratios (RRs).

**Findings:**

Individual patient data were provided for 26 of 33 relevant trials identified, comprising 37 298 (93%) of 40 070 women randomised. Most women were aged younger than 70 years and had node-positive disease. Total cytotoxic drug usage was broadly comparable in the two treatment arms; colony-stimulating factor was generally used in the more dose-intense arm. Combining data from all 26 trials, fewer breast cancer recurrences were seen with dose-intense than with standard-schedule chemotherapy (10-year recurrence risk 28·0% *vs* 31·4%; RR 0·86, 95% CI 0·82–0·89; p<0·0001). 10-year breast cancer mortality was similarly reduced (18·9% *vs* 21·3%; RR 0·87, 95% CI 0·83–0·92; p<0·0001), as was all-cause mortality (22·1% *vs* 24·8%; RR 0·87, 95% CI 0·83–0·91; p<0·0001). Death without recurrence was, if anything, lower with dose-intense than with standard-schedule chemotherapy (10-year risk 4·1% *vs* 4·6%; RR 0·88, 95% CI 0·78–0·99; p=0·034). Recurrence reductions were similar in the seven trials (n=10 004) that compared 2-weekly chemotherapy with the same chemotherapy given 3-weekly (10-year risk 24·0% *vs* 28·3%; RR 0·83, 95% CI 0·76–0·91; p<0·0001), in the six trials (n=11 028) of sequential versus concurrent anthracycline plus taxane chemotherapy (28·1% *vs* 31·3%; RR 0·87, 95% CI 0·80–0·94; p=0·0006), and in the six trials (n=6532) testing both shorter intervals and sequential administration (30·4% *vs* 35·0%; RR 0·82, 95% CI 0·74–0·90; p<0·0001). The proportional reductions in recurrence with dose-intense chemotherapy were similar and highly significant (p<0·0001) in oestrogen receptor (ER)-positive and ER-negative disease and did not differ significantly by other patient or tumour characteristics.

**Interpretation:**

Increasing the dose intensity of adjuvant chemotherapy by shortening the interval between treatment cycles, or by giving individual drugs sequentially rather than giving the same drugs concurrently, moderately reduces the 10-year risk of recurrence and death from breast cancer without increasing mortality from other causes.

**Funding:**

Cancer Research UK, Medical Research Council.

## Introduction

Among women with early breast cancer, standard combination chemotherapy that includes an anthracycline and taxane reduces breast cancer mortality by about a third compared with no chemotherapy.^1^ The proportional reduction is largely independent of oestrogen receptor (ER) status, nodal status, or other classical tumour characteristics, so the absolute benefit from chemotherapy for an individual woman depends mainly on her absolute risk of breast cancer recurrence and death.

Trials investigating newer cytotoxic agents for breast cancer have not yet identified any classes that are clearly superior to taxanes and anthracyclines,^2^, ^3^, ^4^ but the optimal dosage and timing of these two drugs is still unclear. Cell biology and cytokinetic modelling suggest that increasing dose intensity (ie, the amount of drug delivered per unit time)^5^ could enhance tumour cell kill, reduce tumour regrowth between cycles, and thereby further improve the likelihood of cure.^6^, ^7^ However, studies comparing anthracycline doses have shown no apparent benefit from escalating beyond standard doses,^8^ although lesser benefit is seen with doses below this threshold,^9^, ^10^, ^11^ suggesting that dose escalation alone cannot kill all tumour cells. This might be due to the non-linearity of dose–response or clonal variation in chemotherapy sensitivity.^12^ Two ways to increase dose intensity without escalating total dose are to shorten the interval between treatment cycles (dose-dense chemotherapy) or to administer individual drugs in sequence (sequential scheduling), which allows higher doses to be used in each cycle than is possible with combined administration of the same drugs (concurrent scheduling).^7^

**Research in context**

**Evidence before this study**

Previous meta-analyses from the Early Breast Cancer Trialists' Collaborative Group (EBCTCG) have shown that anthracycline and taxane-based combination chemotherapy reduces the risk of breast cancer mortality by about a third compared with no chemotherapy. Cell biology suggests that increasing the dose intensity of chemotherapy—for example, by shortening the interval between treatment cycles or by giving higher doses of drugs individually rather than lower doses concurrently—might improve outcomes; various clinical trials have been designed to test this hypothesis. The EBCTCG's ongoing extensive searches of bibliographic databases including MEDLINE, Embase, the Cochrane Library, and meeting abstracts up to March, 2018, identified 33 trials that compared dose-intensive versus standard schedule chemotherapy, but these trials used a wide range of chemotherapy combinations and schedules, with inconsistent results reported.

**Added value of this study**

This collaborative meta-analysis collated, checked, and analysed individual patient-level data from 37 298 patients in 26 trials, comprising 93% of all patients in identified studies of dose intensification. In seven trials comparing chemotherapy administered every 2 weeks versus the same drugs, doses, and number of cycles administered every 3 weeks, the dose-dense 2-weekly schedule reduced the risk of breast cancer recurrence by 17% compared with standard treatment. The absolute reduction in the 10-year risk of breast cancer recurrence was 4·3%, with a 2·8% reduction in the 10-year risk of dying from breast cancer, and no increase in non-breast cancer deaths. Other dose-intensification strategies of administering individual drugs sequentially rather than concurrently showed similar benefits. The proportional reductions in recurrence were similar for oestrogen receptor (ER)-positive and ER-negative tumours and did not differ significantly by other patient or tumour characteristics.

**Implications of all the available evidence**

In comparison with standard schedules for adjuvant anthracycline and taxane chemotherapy, the use of more dose-intense schedules further reduces the risk of breast cancer recurrence or death without increasing mortality from other causes. If chemotherapy is to be given, a dose-intense regimen should at least be considered.

For most multiple-agent chemotherapy regimens, it was initially not possible to administer treatments with intervals shorter than the standard 3-weekly or 4-weekly cycles because of unacceptable toxicity. However, the introduction of better supportive treatments, particularly granulocyte colony-stimulating factor (G-CSF), has allowed investigation of dose-dense and other dose-intensification schedules. To help clarify the relative benefits and risks of dose-intense and standard-schedule chemotherapy, we did collaborative meta-analyses of individual patient-level data from randomised trials that compared 2-weekly dose-dense chemotherapy with standard 3-weekly or 4-weekly administration, and trials of sequential versus concurrent anthracycline and taxane chemotherapy.

## Methods

### Search strategy and selection criteria

Methods of identifying trials, data collection, checking, analysis, and presentation were as described in previous reports from the Early Breast Cancer Trialists' Collaborative Group (EBCTCG),^1^, ^13^, ^14^, ^15^ and conform with the PRISMA-IPD statement.^16^ Trials of adjuvant or neoadjuvant chemotherapy were eligible if they began before 2008 and randomly assigned women either to chemotherapy with shorter intervals between cycles (dose-dense) or to identical or similar-dose chemotherapy with standard intervals between cycles (3-weekly or 4-weekly), or to anthracycline and taxane-based chemotherapy given sequentially versus concurrently (appendix pp 34–43). Trials of dose fractionation (eg, giving drugs once weekly at approximately a third of the dose used in a 3-weekly regimen)^17^ were not eligible. In general, dose-intense regimens included primary prophylaxis with G-CSF. Information was sought from trial groups during 2015–18 for each individual patient on randomisation date, allocated treatment, age, menopausal status, body-mass index (BMI), tumour diameter, grade, histology, spread to locoregional lymph nodes, oestrogen receptor (ER) status, progesterone receptor (PR) status, HER2 status, proliferation index, dates and sites of any breast cancer recurrence or other second primary cancer, and the date and underlying cause of any death without recurrence.

Primary outcomes were any recurrence of invasive breast cancer (distant, locoregional, or new primary in the contralateral breast), breast cancer mortality (by log-rank subtraction), death without recurrence, and all-cause mortality. Prespecified primary subgroup investigations were by site of recurrence, method of dose intensification, use of taxane, number of escalated cycles (≤4 *vs* ≥6), age, ER and PR status, nodal status, tumour diameter, grade, histology (ductal, lobular), HER2 status, proliferation index (Ki-67 <10%, 10–19%, or ≥20%), and follow-up period (years 0–1, 2–4, 5–9, or ≥10).

### Statistical analysis

Statistical methods (stratified log-rank analyses and Kaplan-Meier graphs) were as described in previous EBCTCG reports.^1^, ^13^, ^14^, ^15^, ^16^ Time-to-event analyses were stratified by age, ER status, trial, and, except for studies including neoadjuvant chemotherapy, nodal status. Each analysis compared all women allocated to dose-intense chemotherapy versus all allocated standard-schedule chemotherapy, regardless of treatment compliance (yielding intention-to-treat analyses). Log-rank statistics were used to assess treatment effects and to estimate the first-event-rate ratio (RR) and its confidence interval (95% CI for meta-analyses and 99% CI for individual trials or subgroups). Breast cancer mortality RRs were estimated from corresponding log-rank analyses of mortality with recurrence (obtained by subtracting log-rank analyses of mortality without recurrence from those of overall mortality, which avoids the need to ascertain which deaths after recurrence were from breast cancer).^14^ For each comparison, forest plots and Kaplan-Meier graphs describe the separate trials and their results, and subgroup analyses explore whether proportional risk reductions depend on patient or tumour-related characteristics. Statistical analyses used in-house Fortran programs.

### Role of the funding source

The funders had no role in study design, data collection, data analysis, data interpretation, or writing of the report. The secretariat had access to all data. The writing committee was responsible for the decision to submit for publication.

## Results

33 relevant trials of dose-intense versus standard-schedule chemotherapy were identified; individual patient-level data were provided for 26 trials, comprising 37 298 (93%) of 40 070 women. Patients were treated between 1985 and 2011. Median follow-up was 7·4 years (IQR 5·9–10·3). Excluding neoadjuvant trials (n=2583), nodal status was known for 33 937 (98%) of 34 715 women. Tumour grade was known for 22 840 (61%) women, oestrogen-receptor (ER) status for 35 929 (96%), HER2 status for 18 673 (50%), and Ki-67 status for 6963 (19%). No gene-expression profiles were available.

**Figure 1:**
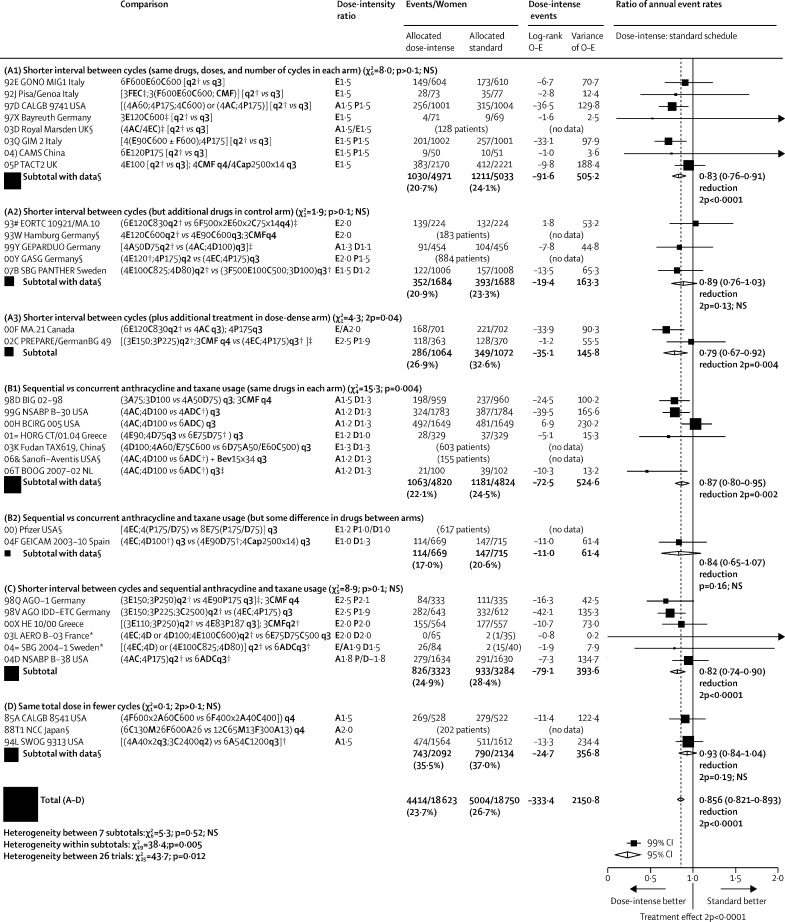
Recurrence in trials testing dose-intense strategies versus standard scheduling Taxanes: D=docetaxel. P=paclitaxel. Anthracyclines: A=doxorubicin. E=epirubicin. Other: C=cyclophosphamide. Cap=capecitabine. F=fluorouracil. M=methotrexate. Bev=bevacizumab (mg/kg). AC=A60C600. EC=E90C600. ADC=A50D75C500.CMF=classical CMFd1d8. Chemotherapy doses are in mg/m^2^. q2=2-weekly. q3=3-weekly. q4=4-weekly. × 14=days 1–14 orally. × 2=day 1, day 8. 2p=two-sided p value. *For balance, the 75 control patients in the two 3-way trials count twice in subtotal (C) and in final total of events/patients. NSABP B-38 trial assumes a 2:1 dose equivalence ratio for **P** to **D**. †Primary prophylaxis with colony-stimulating growth factors. ‡Pre-operative chemotherapy: patients in these trials were analysed as having unknown nodal status. §Seven trials with no data do not contribute to subtotals or to the overall total. Semicolon indicates treatment sequence. First column shows study name and year started. χ^2^ tests in section headers are for heterogeneity between trials.

Figure 1 shows, for each trial, year started, study name, regimens compared, the ratio of dose intensities (mg/m^2^ per week of anthracycline and, if used, taxane), numbers of recurrences, and log-rank analyses, and gives combined results for various trial groupings by summing the trial-specific log-rank statistics. The appendix (pp 3–10) gives similar analyses for distant recurrence at any time, local recurrence as first event, contralateral breast cancer as first event, breast cancer mortality, death without recurrence (first year only, all years), and all-cause mortality. Combining the recurrence results for all 26 trials to test for some effect of dose intensification, the RR was 0·86 (95% CI 0·82–0·89, p<0·0001).

The trials are grouped by how dose intensification was achieved. Groups A1–3 are the dose-dense trials, in which the dose-intense treatment arm had a shorter interval between cycles than the standard-schedule arm. Group A1 trials gave the same chemotherapy drugs, same doses, and same number of cycles in both arms, whereas group A2 trials gave additional drugs in the control arm, and group A3 trials gave additional treatment in the dose-dense arm. Groups B1–2 are the sequential versus concurrent trials, in which the dose-intense arm received anthracycline and taxane sequentially, allowing a higher dose per cycle of one or, generally, both agents. Group B1 trials used the same drugs and the same 3-weekly time intervals between cycles in both arms, whereas in B2 there were some differences in drugs between arms. Finally, group C trials used both methods of increasing dose intensity (shorter interval between cycles and sequential anthracycline and taxane usage), and group D trials gave higher doses per cycle in fewer cycles.

In group A1 (trials of 2-weekly versus 3-weekly chemotherapy cycles using the same drugs and doses), individual patient datasets were provided for seven of the eight relevant trials, comprising 10 004 (99%) of 10 132 women randomised. The average weekly dose of anthracycline (and taxane, if used) in the dose-dense arm was 1·5 times that in the standard-schedule arm. All trials included at least three anthracycline-containing cycles (minimum total dose 240 mg/m^2^ doxorubicin or 360 mg/m^2^ epirubicin). Three trials (comprising 4109 women) also included paclitaxel at 175 mg/m^2^. Growth factors were administered as primary prophylaxis in the 2-weekly cycles and in some studies were permitted, if clinically indicated, in the 3-weekly cycles.

**Figure 2:**
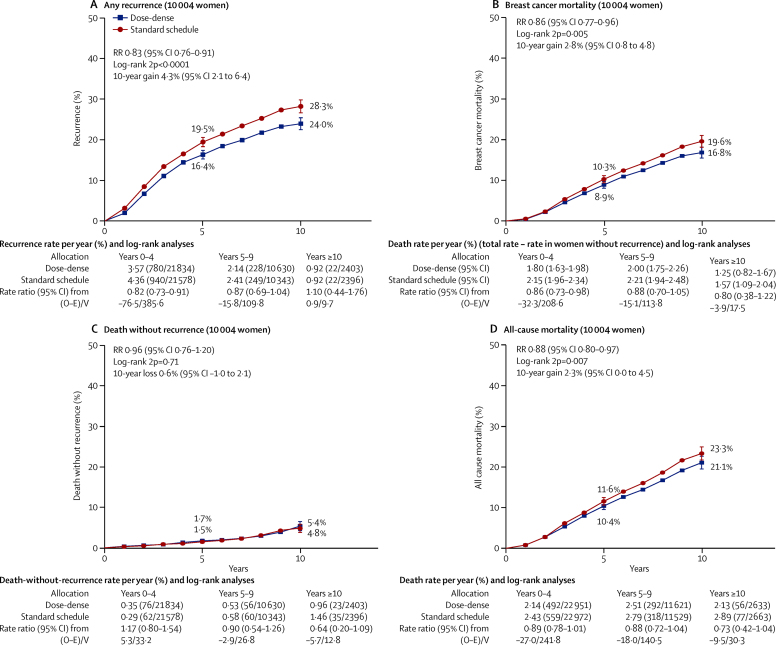
Dose-dense (2-weekly) chemotherapy versus the same chemotherapy given 3-weekly 10-year cumulative risk of any recurrence (A), breast cancer mortality (B), death without recurrence (C), and all-cause mortality (D). Of the 10 004 women, 71% are N+. RR=rate ratio.

The cumulative risks of recurrence, breast cancer mortality, death without recurrence, and death from any cause in these seven trials are shown in figure 2. The 10-year risk of recurrence was 24·0% in the dose-dense arm and 28·3% in the standard-schedule arm (RR 0·83, 95% CI 0·76–0·91; p<0·0001). Exclusion of two studies (GONO and Pisa/Genoa) that used lower doses of anthracycline than are currently standard did not affect the risk reduction (18% without these studies *vs* 17% with these studies; RR 0·82, 95% CI 0·75–0·91; p<0·0001). The proportional reductions were similar for recurrence, breast cancer mortality (RR 0·86, 95% CI 0·77–0·96; p=0·0054), and all-cause mortality (0·88, 0·80–0·97; p=0·007). There was little or no difference in non-breast-cancer mortality (18 deaths *vs* 13 deaths in year 0 and 137 *vs* 144 later).

The proportional reduction in recurrence was similar for ER-negative and ER-positive tumours (RR 0·82, 95% CI 0·71–0·95 *vs* 0·83, 0·75–0·93). Likewise, nodal status and grade did not significantly affect the recurrence RR, although few patients had node-negative or low-grade cancers (appendix pp 11–12).

**Figure 3:**
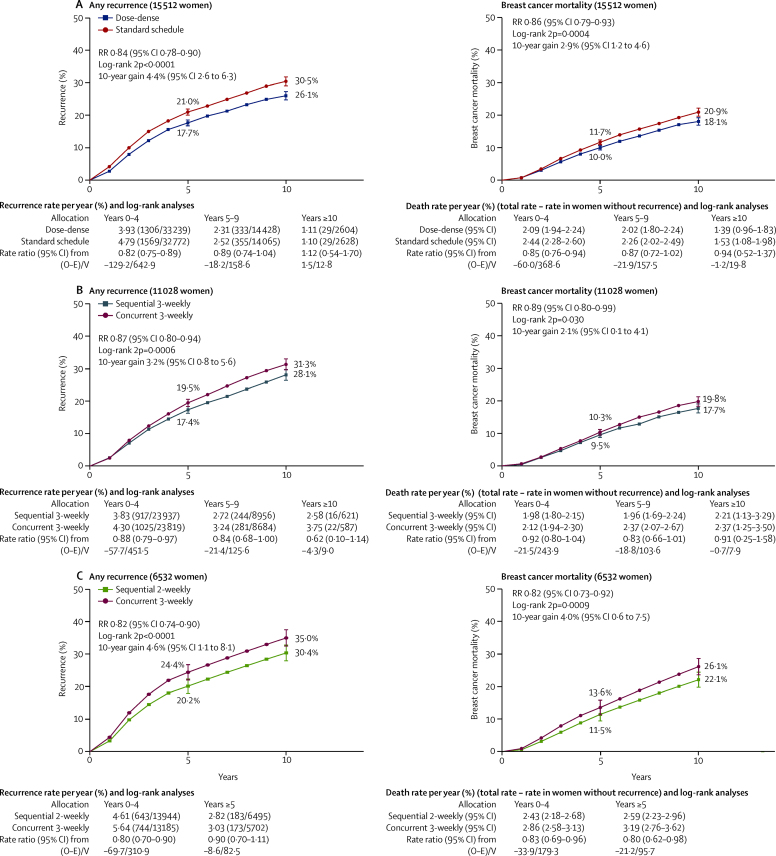
10-year risk of any recurrence (left) and breast cancer death (right) for all trials (confounded and unconfounded) (A) Dose-dense (2-weekly) versus standard schedule (3-weekly) chemotherapy. Of the 15 512 women, 65% are N+. (B) Sequential versus concurrent chemotherapy (both 3-weekly). Of the 11 028 women, 91% are N+. (C) Sequential (2-weekly) versus concurrent (3-weekly) therapy. Of the 6532 women, 90% are N+.

In comparisons A2 and A3 (2-weekly versus 3-weekly or 4-weekly cycles, but with additional drugs in the control or in the dose-dense arm), data were available from five of seven relevant trials, comprising 5508 (84%) of 6575 patients. Three trials had a 2-weekly versus 3-weekly taxane component (one paclitaxel, two docetaxel). As might be expected, the recurrence RR appeared somewhat less extreme in trials with extra drugs in the control arm (RR 0·89, 95% CI 0·76–1·03) than in trials with higher doses or extra drugs in the dose-dense arm (0·79, 0·67–0·92). Combining all trials of dose-dense versus standard-schedule chemotherapy (A1–3, n=15 512) yielded results similar to those in figure 2, but with narrower confidence intervals (recurrence RR 0·84, 95% CI 0·78–0·90; p<0·0001; breast cancer mortality 0·86, 0·79–0·93; p=0·0004; figure 3A; appendix p 13).

In group B1 (sequential versus concurrent anthracycline and taxane scheduling, with no difference in other drugs), information was available from five of seven trials, comprising 9644 (93%) of 10 402 women. These trials compared six to eight cycles of 3-weekly treatment with an anthracycline (usually doxorubicin) followed by a taxane (all single-agent docetaxel) versus four to six cycles of 3-weekly treatment with the same agents given concurrently. The dose-intensity ratio achieved by giving drugs sequentially ranged from 1·0 to 1·5, with a weighted average of 1·23 for anthracycline and 1·32 for docetaxel, and there was a 13% proportional reduction in recurrence (RR 0·87, 95% CI 0·80–0·95; p=0·0016). This recurrence reduction was unchanged when groups B1 and B2 were combined (figure 3B).

Group C comprises the trials that increased dose intensity both by giving 2-weekly rather than 3-weekly treatment and by sequential rather than concurrent anthracycline and taxane usage; information was available from all six such studies (n=6532). By combining the two dose-intensification methods, the ratio of anthracycline dose intensities in the dose-intense arm to that in the standard-schedule arm ranged from 1·5 to 2·5 (figure 1), averaging 2·0 for anthracycline and 1·8 for taxane. The taxane used in three of these trials was paclitaxel, two used docetaxel, and one used paclitaxel (175 mg/m^2^) in the dose-intense arm but docetaxel (75 mg/m^2^) in the control arm. There was an 18% reduction in recurrence (RR 0·82, 95% CI 0·74–0·90; p<0·0001; figure 3C), again favouring the more dose-intense treatment schedule.

In group D, two trials (n=4226) achieved a dose-intensity ratio of 1·5 by administering a 50% higher dose of anthracycline per cycle for four rather than six cycles. The effect on recurrence rates was not significant (RR 0·93, 95% CI 0·84–1·04; p=0·19), but also did not differ significantly from that in the other groups of trials.

**Figure 4:**
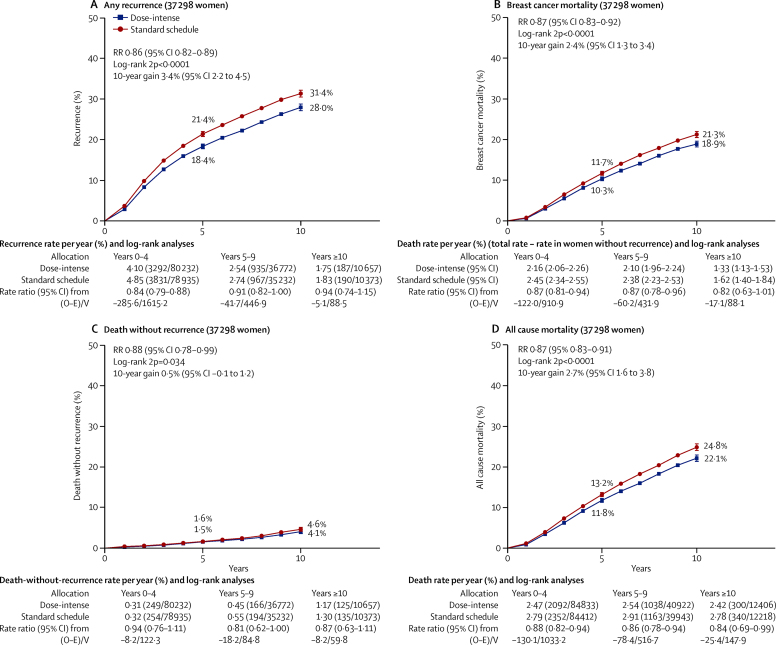
Pooled analysis of all dose-intensification trials 10-year cumulative risk of any recurrence (A), breast cancer mortality (B), death without recurrence (C), all-cause mortality (D), for dose-intense versus standard schedule arm. Of the 37 298 women, 77% are N+. RR=rate ratio.

Despite substantial variation in chemotherapy schedules and trial designs, rate ratios for recurrence for all seven trial groupings shown in figure 1 consistently favoured the more dose-intense treatment arm (p=0·52 for heterogeneity between subtotals). Combining data from all 37 298 women in the 26 dose-intensification studies, the 10-year risk of recurrence for the dose-intense treatment arms was 28·0% compared with 31·4% for standard treatment (gain of 3·4%, 95% CI 2·2–4·5; p<0·0001), with similar improvements in breast cancer mortality (18·9% *vs* 21·3%; gain of 2·4%, 1·3–3·4; p<0·0001), and all-cause mortality (22·1% *vs* 24·8%; gain of 2·7%, 1·6–3·8; p<0·0001; figure 4). These average findings are further subdivided in the appendix (pp 16–21).

Unexpectedly, the 10-year risk of death without recurrence appeared to be somewhat lower in the dose-intense arms than in the standard-schedule arms (4·1% *vs* 4·6%; figure 4C), although this difference was only of borderline significance (RR 0·88, 95% CI 0·78–0·99; p=0·034). Of the deaths without recorded recurrence, half were from an unknown cause, but these were equally divided between dose-intense and control arms (264 [1·4%] of 18 623 *vs* 278 [1·5%] of 18 750; appendix p 22). Additionally, deaths without recurrence were unrelated to tumour size or nodal involvement (appendix p 23), suggesting that most were not misclassified breast cancer deaths.

There was borderline significant (p=0·012) heterogeneity between results of the 26 trials for recurrence (figure 1). Subgroup analyses (appendix pp 14–15) explored whether this heterogeneity might be explained by differences in the numbers of dose-intense treatment cycles or inclusion of a dose-dense versus standard taxane comparison. In the trials with six to eight cycles of 2-weekly versus 3-weekly treatment, 2-weekly chemotherapy was significantly better than standard schedule chemotherapy in preventing recurrence both for anthracycline-based regimens (RR 0·83, 95% CI 0·73–0·95; p=0·0056) and for regimens containing anthracycline plus taxane (0·78, 0·71–0·87; p<0·0001). By contrast, in the trials with only three to four accelerated cycles, reductions in recurrence were not significant regardless of whether taxanes were included or not, although the confidence intervals were wide (RR 0·84, 95% CI 0·63–1·13 with a taxane *vs* 0·94, 0·82–1·09 without a taxane). After combining all trials of dose intensification of anthracycline regimens without a taxane dose-intensification component, including those in group D, the recurrence reduction for such regimens remained significant (RR 0·90, 95% CI 0·84–0·97; p=0·005).

**Figure 5:**
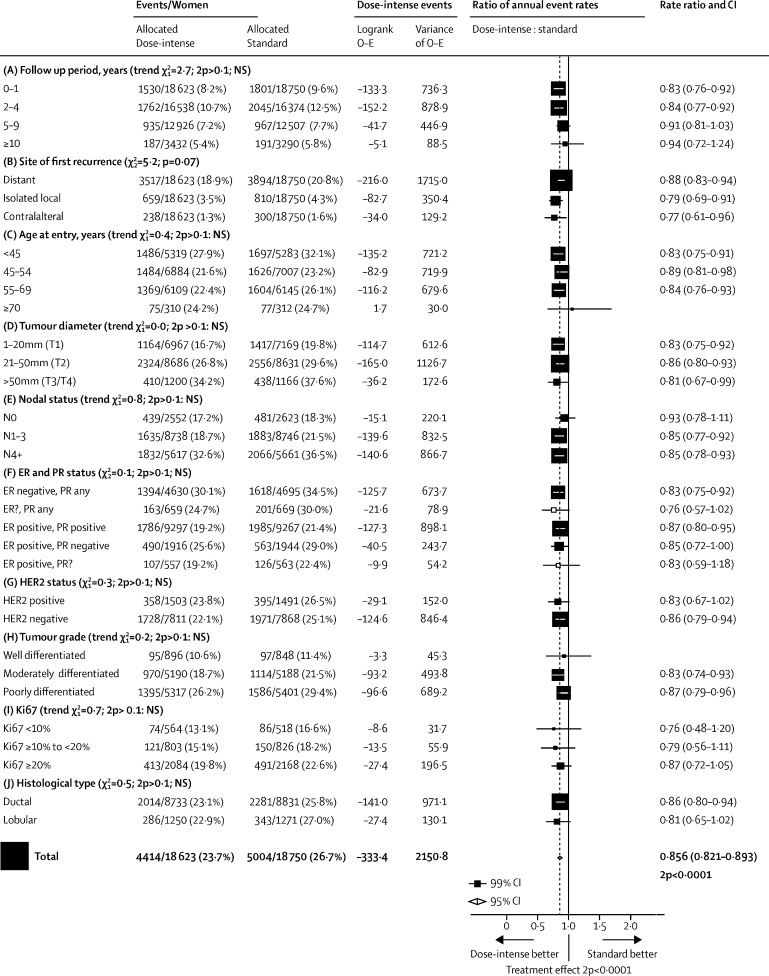
Subset analyses of pooled data from all dose-intensification trials; any first recurrence (including locoregional recurrence, distant recurrence, and new contralateral disease)* ER=oestrogen receptor. PR=progesterone receptor. NS=not significant. *For balance, the 75 control patients in 3-way trials count twice in numbers of events/patients. Unless otherwise specified, patients whose category is unknown are omitted. Unless specified as for trend, χ^2^ tests are for heterogeneity.

To increase the statistical power to investigate whether proportional risk reductions vary by other factors, subgroup analyses (figure 5; appendix p 16) included all women in all trials. The proportional recurrence reduction was highly significant in years 0–1 (RR 0·83, 99% CI 0·76–0·92; p<0·0001) and in years 2–4 (0·84, 0·77–0·92; p<0·0001). The reduction was less definite in years 5–9 (RR 0·91, 99% CI 0·81–1·03; p=0·049) and there were few events thereafter. Distant recurrence (RR 0·87, 95% CI 0·84–0·91; p<0·0001), local recurrence (0·79, 0·71–0·88; p<0·0001), and new contralateral breast cancer (0·77, 0·65–0·91; p=0·0028) were all reduced (appendix p 17).

**Figure 6:**
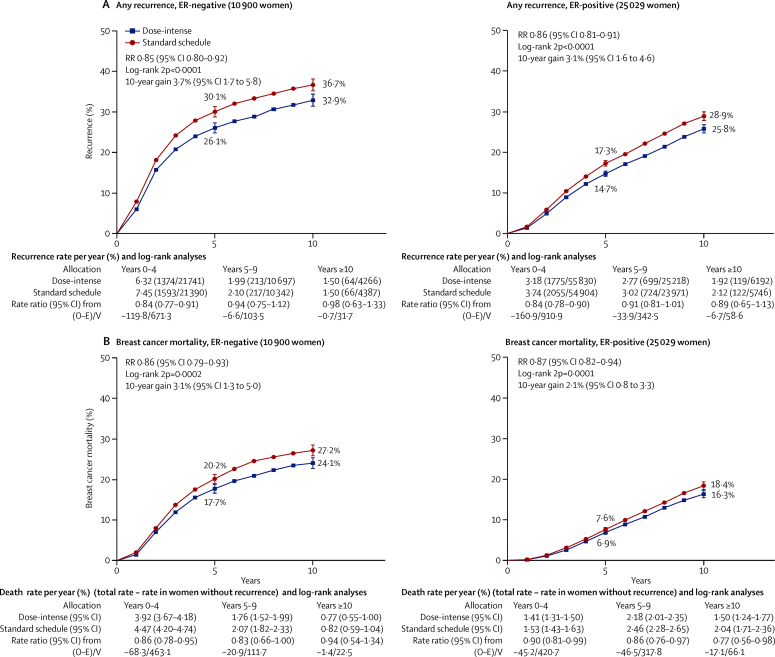
10-year recurrence (A) and breast cancer mortality (B) by oestrogen receptor status Pooled data from all trials of dose intense versus standard schedule chemotherapy. Of the 10 900 women who are oestrogen receptor (ER)-negative, 66% are N+; and of the 25 029 women who are ER-positive, 84% are N+.

Subgroup analyses of recurrence and breast cancer mortality by age, tumour size, nodal status, ER and PR status, HER2 status, grade, tumour proliferation (Ki-67), and histological type showed similar proportional risk reductions with no significant heterogeneity (figure 5; appendix pp 16–21). Although the proportional recurrence reduction was similar in ER-negative and ER-positive breast cancer, the absolute reduction in 10-year recurrence was somewhat larger for ER-negative disease than for ER-positive disease (3·7% *vs* 3·1%; figure 6). For women with node-positive disease, those with four or more involved nodes had substantially worse prognosis than did those with one to three involved nodes; the joint dependence of treatment outcome on ER status and the degree of nodal involvement is given in the appendix (p 20).

There was no increase in deaths without recorded recurrence during the first year after randomisation (0·3% in the dose-intense arm *vs* 0·4% in the standard arm, p=0·053; appendix p 22). There was no difference in deaths from cardiovascular disease (17 *vs* 18, p=0·92), from acute myeloid leukaemia (33 *vs* 27**;** p=0·53), or from other cancers (56 *vs* 66; p=0·27; appendix p 22). Few deaths due to toxicity were reported in trial publications, and numbers did not differ significantly between dose-intense and standard treatment schedules.

Recording of toxicity within each trial varied considerably, so individual patient-level data on non-fatal toxicity were not requested. Adverse events reported in trial publications are, however, summarised in the appendix (pp 24–30). For the 2-weekly dose-dense chemotherapy schedules, primary prophylaxis with G-CSF was mandated in all trials, leading to lower levels of grade 3–4 neutropenia and neutropenic sepsis than in control arms. There were, however, higher levels of grade 3–4 anaemia in the dose-intense arms of some trials. There was no consistent difference in cardiotoxicity or other reported non-haematological toxicity between dose-intense and control arms. There was a moderate increase in the proportion of patients who did not complete all cycles of chemotherapy in the dose-intense compared with standard-schedule arms (11·9% *vs* 9·2%, p<0·0001; appendix p 33), which was mainly explained by patients failing to complete the final courses of the longer sequential therapy regimens. Compliance was similar in both arms in the trials comparing 2-weekly versus 3-weekly chemotherapy using the same drugs, doses, and number of cycles.

## Discussion

This meta-analysis demonstrates that increasing the dose intensity of anthracycline and taxane-based adjuvant chemotherapy regimens for early breast cancer, either by shortening the interval between cycles or by sequential administration of anthracyclines and taxanes (allowing full-dose treatment with each agent), reduces the 10-year risk of recurrence and of death from breast cancer by about 10–15%. This reduction is only moderate but since the standard chemotherapy comparator is already known to reduce the risk of breast cancer death by about a third,^1^ preventing a further 10–15% of deaths would imply that, compared to no chemotherapy, dose-intense adjuvant chemotherapy schedules with anthracycline and taxane could prevent about 40% of deaths from breast cancer during the first decade.

The 26 trials included in this meta-analysis varied substantially in the chemotherapy combinations and in the scheduling used to achieve greater dose intensity. Although this complicates the meta-analysis, the risk reductions were remarkably consistent, with an absolute reduction of about 3% in the 10-year risk of breast cancer mortality in the trials comparing 2-weekly versus 3-weekly chemotherapy cycles, in those of sequential versus concurrent anthracycline and taxane schedules, and in those adopting both strategies, with similar results regardless of whether or not the analyses included the trials with additional drugs or additional cycles given in one arm but not the other.

Heterogeneity between the trial designs makes it difficult to identify reliably more or less effective dose-intensification strategies. Despite limited power to detect any real differences in treatment effects between trial groupings, the largest proportional reduction in breast cancer mortality was seen in the group of trials that used both 2-weekly and sequential scheduling, which also achieved the highest dose-intensity ratios. The smallest proportional reduction was in the sequential versus concurrent 3-weekly scheduling trial group, in which the dose intensity ratio was lowest. In individual trials, the largest risk reductions were generally seen in those with the largest dose-intensity ratios (eg, MA.21,^18^ AGO,^19^ and ETC^20^).

The risk reduction appeared to be smaller in trials with fewer dose-dense cycles than in those with six or more dose-dense cycles, regardless of whether they included an accelerated taxane as well as anthracycline component. The largest trial, TACT2,^21^ compared 2-weekly versus 3-weekly scheduling for only the first four cycles of single-agent epirubicin and achieved one of the smallest risk reductions. The risk reduction with sequential chemotherapy also appeared somewhat lower in trials with six cycles of concurrent chemotherapy in the control arm (BCIRG 005^22^ and NSABP B-38^2^) than in those with just four cycles (AGO,^19^ BIG 02–98,^23^ and NSABP B-30^24^). Similarly, somewhat greater benefit from dose intensification was seen in MA.21,^18^ which compared six 2-weekly cycles in the dose-intense arm with four 3-weekly cycles in the control arm. These findings are consistent with a previous EBCTCG meta-analysis of adding a taxane to anthracycline-based chemotherapy in which longer treatment in the anthracycline control group appeared to negate the benefits of adding a taxane.^1^ By contrast, inclusion of fluorouracil (PANTHER^25^) or cyclophosphamide (GeparDuo^26^) in the standard but not the dose-intensive schedule did not seem important, consistent with trials of adding these agents to anthracycline regimens.^23^, ^24^, ^27^

Five of the dose-dense versus standard taxane comparisons involved paclitaxel (175 mg/m^2^), given 2-weekly versus 3-weekly. Currently, paclitaxel is usually given once weekly at 80 mg/m^2^ as this schedule was found to be superior to 3-weekly paclitaxel at 175 mg/m^2^ in the intergroup E1199 trial,^28^ perhaps because it involved a higher dose intensity (80 mg/m^2^ per week *vs* 58 mg/m^2^ per week). This explanation is supported by the SWOG S0221 study,^17^ which found similar outcomes with once-weekly paclitaxel (80 mg/m^2^) and 2-weekly paclitaxel (175 mg/m^2^), the regimen tested in many trials in our meta-analysis in which the dose intensity is comparable. Moreover, because trials of docetaxel also showed significant benefit from dose intensification (eg, all of the trials of sequential versus concurrent scheduling in section B1 of figure 1 used docetaxel), as did trials with no taxane dose intensification, a better outcome with more dose-intense chemotherapy cannot be accounted for solely by the frequency of paclitaxel administration.

Our meta-analysis therefore suggests that increasing the dose intensity of taxane administration is likely to enhance efficacy irrespective of the agent used or the frequency of administration. We did not identify any trials assessing dose-intensification of taxane regimens without anthracycline, such as the now widely used docetaxel-cyclophosphamide combination. Although our findings suggest that giving this combination 2-weekly instead of the usual 3-weekly administration might enhance efficacy, this would need to be confirmed in randomised trials.

The reduction in new primary contralateral breast cancer was unexpected as no significant reduction in contralateral breast cancer was reported in previous EBCTCG meta-analyses of anthracycline chemotherapy versus no chemotherapy, or in trials of the addition of taxane to anthracycline chemotherapy.^1^ However, in both those meta-analyses there was a borderline significant reduction in contralateral disease in the first 5 years, with no further benefit thereafter. This is consistent with the reduction in new contralateral breast cancer seen in years 0–4 with dose-intense compared to standard schedule chemotherapy (appendix p 17), with no significant additional benefit thereafter, which might be explained by chemotherapy eradicating or delaying the emergence of subclinical contralateral breast cancers but having little impact on the development of future contralateral disease.

Subgroup investigations did not identify any individual patient or tumour characteristic that predicted greater or lesser proportional benefit from dose-intense treatment. In particular, the proportional reductions in recurrence were similar in ER-positive and ER-negative disease. The proportional recurrence reductions were also similar for N1–3 and N4+ disease and appeared to be similar in node-negative disease, although comparatively few women with node-negative disease were enrolled in these trials. This is consistent with other chemotherapy comparisons.^1^ Similarly, although only 50% of patients had known HER2 status, and use of trastuzumab was not routine in some trials, there was no indication that treatment efficacy differed by HER2 status. Also, there was no trend towards increasing benefit with increasing proliferation, as assessed by Ki-67. Too few patients aged older than 70 years, or with low-grade disease, were entered in these trials to directly assess the benefits of dose-intensification in these subgroups.

There was no indication of any increase in death without recurrence with dose intensification, overall or during the period when chemotherapy was administered. No increase in cardiovascular mortality or deaths from haematological malignancies was apparent in patients who received dose-intense treatment, although information on cause of death was incomplete, and longer follow-up is needed to evaluate fully the comparative benefits and risks of dose intensification. Individual patient data on non-fatal toxicity were not collected but trial publications suggest only minor differences between dose-intense and standard chemotherapy. The selective use of colony-stimulating growth factors in the dose-intense treatment arm complicates comparisons of haematological toxicity between treatments, with leucopenia recorded less frequently with dose-intense treatment. Trials that evaluated patient-reported outcomes found overall quality of life to be worse during dose-intense treatment than during standard schedule treatment, but similar quality of life after the treatment phase ended.^21^, ^22^, ^25^, ^29^, ^30^

The balance of benefit versus toxicity, therefore, appears to favour more dose-intense chemotherapy. A further advantage of 2-weekly versus 3-weekly chemotherapy—but not of sequential versus concurrent chemotherapy—is that treatment is completed sooner. As the proportional reductions in recurrence with dose-intense chemotherapy did not differ significantly by any measured tumour characteristic, these findings are applicable to most women who are offered chemotherapy. The present findings are of limited relevance to the question of which women with early breast cancer should be offered chemotherapy, although they do indicate that chemotherapy can reduce breast cancer mortality rates by 40% rather than a third. The absolute gain from this proportional reduction in recurrence depends chiefly on what the risk of distant recurrence would be without chemotherapy, which varies greatly from one woman to another, and is the subject of much ongoing research. The findings are, however, directly relevant to selection of what regimen to use, and they show that, if chemotherapy is to be given, a dose-intense regimen should at least be considered.

Correspondence to: EBCTCG Secretariat, Clinical Trial Service Unit, Nuffield Department of Population Health, Richard Doll Building, Oxford OX3 7LF, UK **bc.overview@ctsu.ox.ac.uk**

## Data sharing

The data sharing policy is available online.
