# Supplementary material for: Increasing the dose intensity of chemotherapy by more frequent administration or sequential scheduling: a patient-level meta-analysis of 37 298 women with early breast cancer in 26 randomised trials
Source: Lancet. 2019 Apr 6;393(10179):1440–52. doi: 10.1016/S0140-6736(18)33137-4 (PMC6451189; doi:10.1016/S0140-6736(18)33137-4)
Supplement: Supplementary appendix [file mmc1.pdf]

THE LANCET

Supplementary appendix

This appendix formed part of the original submission and has been peer reviewed. We post it as supplied by the authors.

Supplement to: Early Breast Cancer Trialists' Collaborative Group (EBCTCG). Increasing the dose intensity of chemotherapy by more frequent administration or sequential scheduling: a patient-level meta-analysis of 37 298 women with early breast cancer in 26 randomised trials. *Lancet* 2019; published online Feb 7. http://dx.doi.org/10.1016/S0140-6736(18)33137-4.

Webappendix: Supplementary figures and tables for "Increasing the dose intensity of chemotherapy by more frequent administration or sequential scheduling: patient level meta-analysis of 37,298 women with early breast cancer in 26 randomised trials"

CONTENTS LIST (Click on any listed item with a page number to jump to it)

Pages 3-10: All trials of dose-intense vs standard schedule chemotherapy: forest plots, one line per trial, for 8 different endpoints (any recurrence, distant recurrence at any time, local recurrence as first event, contralateral breast cancer as first event, breast cancer mortality, death without recurrence in year 0, death without recurrence, all-cause mortality)

Page 11: Trials of dose-dense (2-weekly) chemotherapy vs the same chemotherapy in standard (3-weekly) schedule: subgroup analyses for recurrence

Page 12: 5-year risk of recurrence by ER status in trials of dose-dense (2-weekly) chemotherapy vs the same chemotherapy (3-weekly) schedule [A1]

Page 13: All trials of dose-dense (2-weekly) vs standard schedule (3 or 4-weekly) chemotherapy: subgroup analyses for recurrence

Page 14: All trials of dose-dense (2-weekly) vs standard schedule (3 or 4-weekly) chemotherapy: forest plot of recurrence split by trials with and without taxane, and by those with 4 or fewer or 6 or more dose-dense cycles

Page 15: 10-year risk of recurrence and breast cancer mortality in all dose-dense trials with 6 or more dose-dense cycles (A) anthracycline only, and (B) anthracycline and taxane

Pages 16-23: All trials of dose-intense vs standard schedule chemotherapy

- **Page 16: Subgroup analyses for breast cancer mortality**
- **Page 17: 10-year risk of** distant recurrence at any time, distant recurrence as first event, local recurrence as first event and contralateral breast cancer as first event
- **Pages 18-21: 5 or 10-year recurrence within subgroups** for tumour size, nodal status, ER/nodal status, tumour grade
- **Page 22: Mortality by cause and incidence of second cancers**
- **Page 23: Sensitivity analyses for death without recurrence**

Pages 24–30: Toxicity (as reported in dose intensification study reports)

Pages 31-32: Cardiotoxicity (as reported in dose intensification study reports)

Page 33: Compliance (as reported in study reports if patient level data not provided)

Pages 34-43: Statistical Analysis Plan including list of relevant trials and publications, and of variables requested

P3: Recurrence in trials of dose-intense vs standard schedule chemotherapy

Year code and study name	Comparison	Dose–intensity ratio	Events/Women: Allocated Dose–intense	Events/Women: Allocated Standard	Dose–intense events: Logrank O–E	Dose–intense events: Variance of O–E	Ratio of annual event rates Dose–intense : Standard
**(A1) Shorter interval between cycles (same drugs, doses and number of cycles in each arm)** (χ^2_6 = 8.0; p > 0.1; NS)							
92E GONO MIG1 Italy	6F$_{600}$E$_{60}$C$_{600}$ [q2† v q3]	E1.5	149/604	173/610	–6·7	70·7	
92J Pisa/Genoa Italy	[3FEC‡;3(F$_{600}$E$_{60}$C$_{600}$; CMF)] [q2† v q3]	E1.5	28/73	35/77	–2·8	12·4	
97D CALGB 9741 USA	[(4A$_{60}$;4P$_{175}$;4C$_{600}$) or (4AC;4P$_{175}$)][q2† v q3]	A1.5 P1.5	256/1001	315/1004	–36·5	129·8	
97X Bayreuth Germany	3E$_{120}$C$_{600}$‡ [q2† v q3]	E1.5	4/71	9/69	–1·6	2·5	
03D Royal Marsden UK §	(4AC/4EC)‡ [q2† v q3]	A1.5 / E1.5	(128 patients)		(no data)		
03Q GIM 2 Italy	[4(E$_{90}$C$_{600}$ ± F$_{600}$);4P$_{175}$] [q2† v q3]	E1.5 P1.5	201/1002	257/1001	–33·1	97·9	
04) CAMS China	6E$_{120}$P$_{175}$ [q2† v q3]	E1.5 P1.5	9/50	10/51	–1·0	3·6	
05P TACT2 UK	4E$_{100}$ [q2† v q3];4CMF q4/4Cap$_{2500x14}$ q3	E1.5	383/2170	412/2221	–9·8	188·4	
**(A1) subtotal with data §**			**1030/ 4971 (20·7%)**	**1211/ 5033 (24·1%)**	**–91·6**	**505·2**	**0·83 (0·76 – 0·91) reduction 2p = 0·00005**
**(A2) Shorter interval between cycles (but additional drugs in control arm)** (χ^2_2 = 1.9; p > 0.1; NS)							
93# EORTC 10921/MA.10	(6E$_{120}$C$_{830}$q2† v 6F$_{500x2}$E$_{60x2}$C$_{75x14}$q4)‡	E2.0	139/224	132/224	1·8	53·2	
93W Hamburg Germany §	4E$_{120}$C$_{600}$q2† v 4E$_{90}$C$_{600}$q3;3CMFq4	E2.0	(183 patients)		(no data)		
99Y GEPARDUO Germany	[4A$_{50}$D$_{75}$q2† v (4AC;4D$_{100}$)q3]‡	A1.3 D1.1	91/454	104/456	–7·8	44·8	
00Y GASG Germany §	(4E$_{120}$†;4P$_{175}$)q2 v (4EC;4P$_{175}$)q3	E2.0 P1.5	(884 patients)		(no data)		
07B SBG PANTHER Sweden	(4E$_{100}$C$_{825}$;4D$_{80}$)q2† v (3F$_{500}$E$_{100}$C$_{500}$;3D$_{100}$)q3†	E1.5 D1.2	122/1006	157/1008	–13·5	65·3	
**(A2) subtotal with data §**			**352/ 1684 (20·9%)**	**393/ 1688 (23·3%)**	**–19·4**	**163·3**	**0·89 (0·76 – 1·03) reduction 2p > 0·1; NS**
**(A3) Shorter interval between cycles (plus additional treatment in dose–dense arm)** (χ^2_1 = 4.3; 2p = 0.04)							
00F MA.21 Canada	(6E$_{120}$C$_{830}$q2† v 4AC q3); 4P$_{175}$q3	E/A2.0	168/701	221/702	–33·9	90·3	
02C PREPARE/GermanBG 49	[(3E$_{150}$;3P$_{225}$)q2†;3CMF q4 v (4EC;4P$_{175}$)q3†]‡	E2.5 P1.9	118/363	128/370	–1·2	55·5	
**(A3) subtotal**			**286/ 1064 (26·9%)**	**349/ 1072 (32·6%)**	**–35·1**	**145·8**	**0·79 (0·67 – 0·92) reduction 2p = 0·004**
**(B1) Sequential vs concurrent anthracycline/taxane usage (same drugs in each arm)** (χ^2_4 = 15.3; p = 0.004)							
98D BIG 02–98	(3A$_{75}$;3D$_{100}$ v 4A$_{50}$D$_{75}$) q3; 3CMF q4	A1.5 D1.3	198/959	237/960	–24·5	100·2	
99G NSABP B–30 USA	(4AC;4D$_{100}$ v 4ADC†) q3	A1.2 D1.3	324/1783	387/1784	–39·5	165·6	
00H BCIRG 005 USA	(4AC;4D$_{100}$ v 6ADC) q3	A1.2 D1.3	492/1649	481/1649	6·9	230·2	
01= HORG CT/01.04 Greece	(4E$_{90}$;4D$_{75}$q3 v 6E$_{75}$D$_{75}$†) q3	E1.2 D1.0	28/329	37/329	–5·1	15·3	
03K Fudan TAX 619 China §	(4D$_{100}$;4A$_{60}$/E$_{75}$C$_{600}$ v 6D$_{75}$A$_{50}$/E$_{60}$C$_{500}$) q3	E1.3 D1.3	(603 patients)		(no data)		
06& Sanofi–Aventis USA §	(4AC;4D$_{100}$ v 6ADC)† + Bev$_{15x34}$ q3	A1.2 D1.3	(155 patients)		(no data)		
06T BOOG 2007–02 NL	(4AC;4D$_{100}$ v 6ADC†) q3‡	A1.2 D1.3	21/100	39/102	–10·3	13·2	
**(B1) subtotal with data §**			**1063/ 4820 (22·1%)**	**1181/ 4824 (24·5%)**	**–72·5**	**524·6**	**0·87 (0·80 – 0·95) reduction 2p = 0·002**
**(B2) Sequential vs concurrent anthracycline/taxane usage (but some difference in drugs between arms)**							
00) Pfizer USA §	[4EC;4(P$_{175}$/D$_{75}$) v 8E$_{75}$(P$_{175}$/D$_{75}$)] q3	E1.2 P1.0 / D1.0	(617 patients)		(no data)		
04F GEICAM 2003–10 Spain	(4EC;4D$_{100}$†) q3 v (4E$_{90}$D$_{75}$†;4Cap$_{2500x14}$) q3	E1.0 D1.3	114/669	147/715	–11·0	61·4	
**(B2) subtotal with data §**			**114/ 669 (17·0%)**	**147/ 715 (20·6%)**	**–11·0**	**61·4**	**0·84 (0·65 – 1·07) reduction 2p > 0·1; NS**
**(C) Shorter interval between cycles AND sequential anthracycline/taxane usage** (χ^2_5 = 8.9; p > 0.1; NS)							
98Q AGO–1 Germany	[(3E$_{150}$;3P$_{250}$)q2† v 4E$_{90}$P$_{175}$ q3]‡; 3CMF q4	E2.5 P2.1	84/333	111/335	–16·3	42·5	
98V AGO IDD–ETC Germany	(3E$_{150}$;3P$_{225}$;3C$_{2500}$)q2† v (4EC;4P$_{175}$) q3	E2.5 P1.9	282/643	322/612	–42·1	135·3	
00X HE 10/00 Greece	[(3E$_{110}$;3P$_{250}$)q2† v 4E$_{83}$P$_{187}$ q3]; 3CMFq2†	E2.0 P2.0	155/564	177/557	–10·7	73·0	
03L AERO B–03 France *	(4EC;4D or 4D$_{100}$;4E$_{100}$C$_{600}$)q2† v 6E$_{75}$D$_{75}$C$_{500}$ q3	E2.0 D2.0	0/65	2(1/35)	–0·8	0·2	
04= SBG 2004–1 Sweden *	[(4EC;4D) or (4E$_{100}$C$_{825}$;4D$_{80}$)] q2† v 6ADCq3†	E/A1.9 D1.5	26/84	2(15/40)	–1·9	7·9	
04D NSABP B–38 USA	(4AC;4P$_{175}$)q2† v 6ADCq3†	A1.8 P/D~1.8	279/1634	291/1630	–7·3	134·7	
**(C) subtotal**			**826/ 3323 (24·9%)**	**933/ 3284 (28·4%)**	**–79·1**	**393·6**	**0·82 (0·74 – 0·90) reduction 2p = 0·00007**
**(D) Same total dose in fewer cycles** (χ^2_1 = 0.1; 2p > 0.1; NS)							
85A CALGB 8541 USA	(4F$_{600x2}$A$_{60}$C$_{600}$ v 6F$_{400x2}$A$_{40}$C$_{400}$]) q4	A1.5	269/528	279/522	–11·4	122·4	
88T NCC Japan §	(6C$_{130}$M$_{26}$F$_{600}$A$_{26}$ v 12C$_{65}$M$_{13}$F$_{300}$A$_{13}$) q4	A2.0	(202 patients)		(no data)		
94L SWOG 9313 USA	[(4A$_{40x2}$q3;3C$_{2400}$q2) v 6A$_{54}$C$_{1200}$q3]†	A1.5	474/1564	511/1612	–13·3	234·4	
**(D) subtotal with data §**			**743/ 2092 (35·5%)**	**790/ 2134 (37·0%)**	**–24·7**	**356·8**	**0·93 (0·84 – 1·04) reduction 2p > 0·1; NS**
**Total (A...D)**			**4414/ 18623 (23·7%)**	**5004/ 18750 (26·7%)**	**–333·4**	**2150·8**	**0·856 (0·821 – 0·893) reduction 2p < 0·00001**

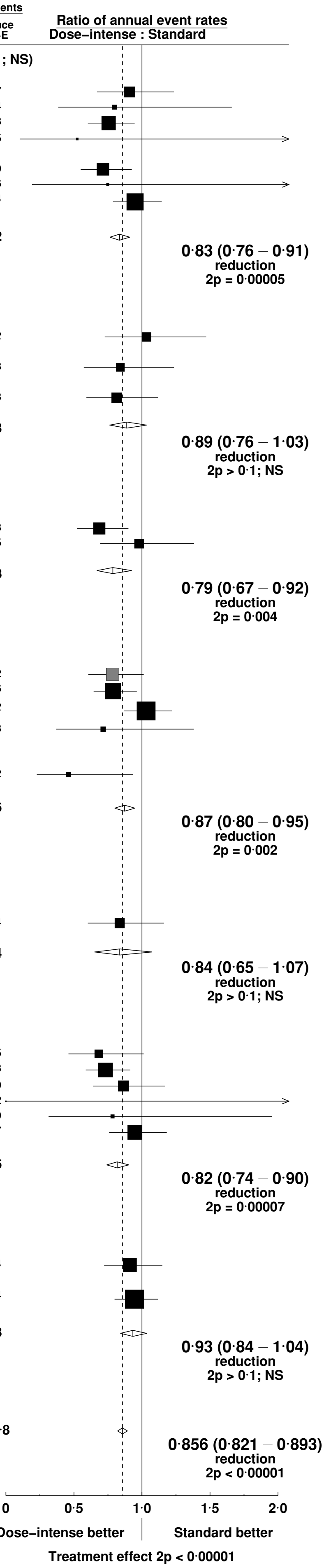

■ 99% or ◇ 95% confidence intervals

Heterogeneity between 7 subtotals: χ^2_6 = 5·3; p > 0·1; NS

Heterogeneity within subtotals: χ^2_{19} = 38·4; p = 0·005

Heterogeneity between 26 trials: χ^2_{25} = 43·7; p = 0·01

Abbreviations: Taxanes: **D** = docetaxel; **P** = paclitaxel. Anthracyclines: **A** = doxorubicin; **E** = epirubicin. Other: **C** = cyclophosphamide; **Cap** = capecitabine; **F** = fluorouracil; **M** = methotrexate; **Bev** = bevacizumab (mg/kg). **AC=A60C600; EC=E90C600; ADC= A50D75C500; CMF** = classical CMFd1d8. Chemotherapy doses are in mg/m2. **q2** = 2-weekly, **q3** = 3-weekly, **q4** = 4-weekly. ×14=days 1-14 orally. x2=day 1, day 8. 2p=two-sided p value. * For balance, the 75 control patients in the two 3-way trials count twice in subtotal (C) and in final total of events/patients. NSABP B-38 trial assumes a 2:1 dose equivalence ratio for P to D. ‡ Pre-operative chemotherapy: patients in these trials were analysed as having unknown nodal status. † Primary prophylaxis with colony-stimulating growth factors. § 7 trials with no data do not contribute to subtotals or to the overall total. Semicolon [;] indicates treatment sequence. χ2 tests in section headers are for heterogeneity between trials.

P4: Distant recurrence at any time in trials of dose-intense vs standard schedule chemotherapy

Year code and study name	Comparison	Dose-intensity ratio	Events/Women: Allocated Dose-intense	Events/Women: Allocated Standard	Dose-intense events: Logrank O−E	Dose-intense events: Variance of O−E	Ratio of annual event rates Dose-intense : Standard
**(A1) Shorter interval between cycles (same drugs, doses and number of cycles in each arm)** (χ^2_6 = 6.7; p > 0.1; NS)							
92E GONO MIG 1 Italy	6$F_{600}E_{60}C_{600}$ [q2† v q3]	E1.5	116/604	137/610	−6·3	56·4	
92J Pisa/Genoa Italy	[3FEC‡;3($F_{600}E_{60}C_{600}$; CMF)] [q2† v q3]	E1.5	21/73	25/77	−1·7	9·5	
97D CALGB 9741 USA	[(4A_{60};4P_{175};4C_{600}) or (4AC;4P_{175})][q2† v q3]	A1.5 P1.5	232/1001	282/1004	−28·6	117·4	
97X Bayreuth Germany	3$E_{120}C_{600}$‡ [q2† v q3]	E1.5	3/71	6/69	−1·1	1·8	
03D Royal Marsden UK §	(4AC/4EC)‡ [q2† v q3]	A1.5 / E1.5	(128 patients)		(no data)		
03Q GIM 2 Italy	[4($E_{90}C_{600}$ ± F_{600});4P_{175}] [q2† v q3]	E1.5 P1.5	174/1002	228/1001	−31·2	86·7	
04) CAMS China	6$E_{120}P_{175}$ [q2† v q3]	E1.5 P1.5	9/50	10/51	−1·0	3·6	
05P TACT2 UK	4E_{100} [q2† v q3];4CMF q4/4$Cap_{2500x14}$ q3	E1.5	312/2170	334/2221	−6·8	153·6	
**(A1) subtotal with data §**			**867/ 4971 (17·4%)**	**1022/ 5033 (20·3%)**	**−76·6**	**429·0**	**0·84 (0·76 – 0·92) reduction 2p = 0·0002**
**(A2) Shorter interval between cycles (but additional drugs in control arm)** (χ^2_2 = 0.7; p > 0.1; NS)							
93# EORTC 10921/MA.10	(6$E_{120}C_{830}$q2† v 6$F_{500x2}E_{60x2}C_{75x14}$q4)‡	E2.0	127/224	124/224	1·0	51·0	
93W Hamburg Germany §	4$E_{120}C_{600}$q2† v 4$E_{90}C_{600}$q3;3CMFq4	E2.0	(183 patients)		(no data)		
99Y GEPARDUO Germany	[4$A_{50}D_{75}$q2† v (4AC;4D_{100})q3]‡	A1.3 D1.1	73/454	79/456	−3·0	35·8	
00Y GASG Germany §	(4E_{120}†;4P_{175})q2 v (4EC;4P_{175})q3	E2.0 P1.5	(884 patients)		(no data)		
07B SBG PANTHER Sweden	(4$E_{100}C_{825}$;4D_{80})q2† v (3$F_{500}E_{100}C_{500}$;3D_{100})q3†	E1.5 D1.2	110/1006	134/1008	−8·0	57·5	
**(A2) subtotal with data §**			**310/ 1684 (18·4%)**	**337/ 1688 (20·0%)**	**−9·9**	**144·3**	**0·93 (0·79 – 1·10) reduction 2p > 0·1; NS**
**(A3) Shorter interval between cycles (plus additional treatment in dose−dense arm)** (χ^2_1 = 3.8; 2p = 0.05)							
00F MA.21 Canada	(6$E_{120}C_{830}$q2† v 4AC q3); 4P_{175}q3	E/A2.0	128/701	176/702	−27·6	71·4	
02C PREPARE/GermanBG 4§	(3E_{150};3P_{225})q2†;3CMF q4 v (4EC;4P_{175})q3†]‡	E2.5 P1.9	102/363	113/370	−1·2	49·0	
**(A3) subtotal**			**230/ 1064 (21·6%)**	**289/ 1072 (27·0%)**	**−28·8**	**120·4**	**0·79 (0·66 – 0·94) reduction 2p = 0·009**
**(B1) Sequential vs concurrent anthracycline/taxane usage (same drugs in each arm)** (χ^2_4 = 13.5; p = 0.009)							
98D BIG 02−98	(3A_{75};3D_{100} v 4$A_{50}D_{75}$) q3; 3CMF q4	A1.5 D1.3	179/959	212/960	−19·9	91·0	
99G NSABP B−30 USA	(4AC;4D_{100} v 4ADC†) q3	A1.2 D1.3	277/1783	317/1784	−26·1	139·8	
00H BCIRG 005 USA	(4AC;4D_{100} v 6ADC) q3	A1.2 D1.3	439/1649	417/1649	12·1	203·3	
01= HORG CT/01.04 Greece	(4E_{90};4D_{75}q3 v 6$E_{75}D_{75}$†) q3	E1.2 D1.0	23/329	31/329	−3·8	12·8	
03K Fudan TAX 619 China §	(4D_{100};4A_{60}/$E_{75}C_{600}$ v 6$D_{75}A_{50}$/$E_{60}C_{500}$) q3	E1.3 D1.3	(603 patients)		(no data)		
06& Sanofi−Aventis USA §	(4AC;4D_{100} v 6ADC)† + Bev_{15x34} q3	A1.2 D1.3	(155 patients)		(no data)		
06T BOOG 2007−02 NL	(4AC;4D_{100} v 6ADC†) q3‡	A1.2 D1.3	21/100	37/102	−9·6	12·6	
**(B1) subtotal with data §**			**939/ 4820 (19·5%)**	**1014/ 4824 (21·0%)**	**−47·3**	**459·4**	**0·90 (0·82 – 0·99) reduction 2p = 0·03**
**(B2) Sequential vs concurrent anthracycline/taxane usage (but some difference in drugs between arms)**							
00) Pfizer USA §	[4EC;4(P_{175}/D_{75}) v 8E_{75}(P_{175}/D_{75})] q3	E1.2 P1.0 / D1.0	(617 patients)		(no data)		
04F GEICAM 2003−10 Spain	(4EC;4D_{100}†) q3 v (4$E_{90}D_{75}$†;4$Cap_{2500x14}$) q3	E1.0 D1.3	93/669	115/715	−6·4	49·3	
**(B2) subtotal with data §**			**93/ 669 (13·9%)**	**115/ 715 (16·1%)**	**−6·4**	**49·3**	**0·88 (0·66 – 1·16) reduction 2p > 0·1; NS**
**(C) Shorter interval between cycles AND sequential anthracycline/taxane usage** (χ^2_5 = 8.8; p > 0.1; NS)							
98Q AGO−1 Germany	[(3E_{150};3P_{250})q2† v 4$E_{90}P_{175}$ q3]‡; 3CMF q4	E2.5 P2.1	84/333	111/335	−16·3	42·5	
98V AGO IDD−ETC Germany	(3E_{150};3P_{225};3C_{2500})q2† v (4EC;4P_{175}) q3	E2.5 P1.9	270/643	306/612	−38·1	129·4	
00X HE 10/00 Greece	[(3E_{110};3P_{250})q2† v 4$E_{83}P_{187}$ q3]; 3CMFq2†	E2.0 P2.0	144/564	164/557	−10·4	68·1	
03L AERO B−03 France *	(4EC;4D or 4D_{100};4$E_{100}C_{600}$)q2† v 6$E_{75}D_{75}C_{500}$ q3	E2.0 D2.0	0/65	2(1/35)	−0·8	0·2	
04= SBG 2004−1 Sweden *	[(4EC;4D) or (4$E_{100}C_{825}$;4D_{80})] q2† v 6ADCq3†	E/A1.9 D1.5	23/84	2(14/40)	−2·1	7·3	
04D NSABP B−38 USA	(4AC;4P_{175})q2† v 6ADCq3†	A1.8 P/D~1.8	243/1634	248/1630	−3·8	116·7	
**(C) subtotal**			**764/ 3323 (23·0%)**	**859/ 3284 (26·2%)**	**−71·4**	**364·3**	**0·82 (0·74 – 0·91) reduction 2p = 0·0002**
**(D) Same total dose in fewer cycles** (χ^2_1 = 0.5; 2p > 0.1; NS)							
85A CALGB 8541 USA	(4$F_{600x2}A_{60}C_{600}$ v 6$F_{400x2}A_{40}C_{400}$]) q4	A1.5	228/528	220/522	1·3	102·2	
88T NCC Japan §	(6$C_{130}M_{26}F_{600}A_{26}$ v 12$C_{65}M_{13}F_{300}A_{13}$) q4	A2.0	(202 patients)		(no data)		
94L SWOG 9313 USA	[(4A_{40x2}q3;3C_{2400}q2) v 6$A_{54}C_{1200}$q3]†	A1.5	454/1564	496/1612	−15·8	225·9	
**(D) subtotal with data §**			**682/ 2092 (32·6%)**	**716/ 2134 (33·6%)**	**−14·5**	**328·2**	**0·96 (0·86 – 1·07) reduction 2p > 0·1; NS**
**Total (A...D)**			**3885/ 18623 (20·9%)**	**4352/ 18750 (23·2%)**	**−254·9**	**1895·0**	**0·874 (0·836 – 0·914) reduction 2p < 0·00001**

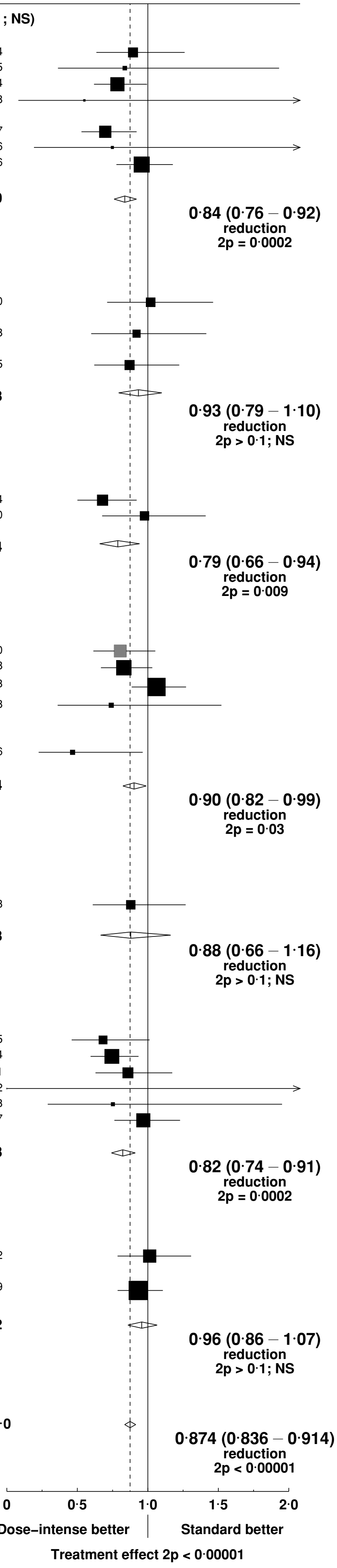

99% or 95% confidence intervals

Heterogeneity between 7 subtotals: χ^2_6 = 7·3; p > 0·1; NS

Heterogeneity within subtotals: χ^2_{19} = 34·0; p = 0·02

Heterogeneity between 26 trials: χ^2_{25} = 41·3; p = 0·02

Abbreviations: Taxanes: **D** = docetaxel; **P** = paclitaxel. Anthracyclines: **A** = doxorubicin; **E** = epirubicin. Other: **C** = cyclophosphamide; **Cap** = capecitabine; **F** = fluorouracil; **M** = methotrexate; **Bev** = bevacizumab (mg/kg). **AC**=A60C600; **EC**=E90C600; **ADC**= A50**D**75C500; **CMF** = classical CMFd1d8. Chemotherapy doses are in mg/m2. **q2** = 2-weekly, **q3** = 3-weekly, **q4** = 4-weekly. ×14=days 1-14 orally. x2=day 1, day 8. 2p=two-sided p value. * For balance, the 75 control patients in the two 3-way trials count twice in subtotal (C) and in final total of events/patients. NSABP B-38 trial assumes a 2:1 dose equivalence ratio for P to D. ‡ Pre-operative chemotherapy: patients in these trials were analysed as having unknown nodal status. † Primary prophylaxis with colony-stimulating growth factors. § 7 trials with no data do not contribute to subtotals or to the overall total. Semicolon [;] indicates treatment sequence. χ2 tests in section headers are for heterogeneity between trials.

P5: Local recurrence as first event in trials of dose-intense vs standard schedule chemotherapy

Year code and study name	Comparison	Dose-intensity ratio	Events/Women: Allocated Dose-intense	Events/Women: Allocated Standard	Dose-intense events: Logrank O−E	Dose-intense events: Variance of O−E	Ratio of annual event rates Dose-intense : Standard
**(A1) Shorter interval between cycles (same drugs, doses and number of cycles in each arm)** (χ^2_5 = 2.6; p > 0.1; NS)							
92E GONO MIG1 Italy	6F600E60C600 [q2† v q3]	E1.5	31/604	40/610	−4·8	16·7	
92J Pisa/Genoa Italy	[3FEC‡;3(F600E60C600; CMF)] [q2† v q3]	E1.5	6/73	6/77	−0·1	2·5	
97D CALGB 9741 USA	[(4A60;4P175;4C600) or (4AC;4P175)][q2† v q3]	A1.5 P1.5	45/1001	59/1004	−8·8	24·8	
97X Bayreuth Germany	3E120C600‡ [q2† v q3]	E1.5	1/71	3/69	−0·5	0·8	
03D Royal Marsden UK §	(4AC/4EC)‡ [q2† v q3]	A1.5 / E1.5	*(128 patients)*		*(no data)*		
03Q GIM 2 Italy	[4(E90C600 ± F600);4P175] [q2† v q3]	E1.5 P1.5	24/1002	29/1001	−2·9	12·0	
04) CAMS China	6E120P175 [q2† v q3]	E1.5 P1.5	0/50	0/51			
05P TACT2 UK	4E100 [q2† v q3];4CMF q4/4Cap2500x14 q3	E1.5	93/2170	96/2221	−0·4	46·5	
**(A1) subtotal with data §**			**200/ 4971 (4·0%)**	**233/ 5033 (4·6%)**	**−17·5**	**103·4**	**0·84 (0·70 – 1·02) reduction 2p = 0·09**
**(A2) Shorter interval between cycles (but additional drugs in control arm)** (χ^2_2 = 0.0; p > 0.1; NS)							
93# EORTC 10921/MA.10	(6E120C830q2† v 6F500x2E60x2C75x14q4)‡	E2.0	38/224	53/224	−7·3	20·2	
93W Hamburg Germany §	4E120C600q2† v 4E90C600q3;3CMFq4	E2.0	*(183 patients)*		*(no data)*		
99Y GEPARDUO Germany	[4A50D75q2† v (4AC;4D100)q3]‡	A1.3 D1.1	28/454	39/456	−6·0	15·7	
00Y GASG Germany §	(4E120†;4P175)q2 v (4EC;4P175)q3	E2.0 P1.5	*(884 patients)*		*(no data)*		
07B SBG PANTHER Sweden	(4E100C825;4D80)q2† v (3F500E100C500;3D100)q3†	E1.5 D1.2	21/1006	30/1008	−4·7	12·4	
**(A2) subtotal with data §**			**87/ 1684 (5·2%)**	**122/ 1688 (7·2%)**	**−18·0**	**48·3**	**0·69 (0·52 – 0·91) reduction 2p = 0·010**
**(A3) Shorter interval between cycles (plus additional treatment in dose−dense arm)** (χ^2_1 = 4.5; 2p = 0.03)							
00F MA.21 Canada	(6E120C830q2† v 4AC q3); 4P175q3	E/A2.0	33/701	69/702	−20·4	24·7	
02C PREPARE/GermanBG 49	[(3E150;3P225)q2†;3CMF q4 v (4EC;4P175)q3†]‡	E2.5 P1.9	27/363	30/370	−1·4	13·5	
**(A3) subtotal**			**60/ 1064 (5·6%)**	**99/ 1072 (9·2%)**	**−21·8**	**38·1**	**0·57 (0·41 – 0·78) reduction 2p = 0·0004**
**(B1) Sequential vs concurrent anthracycline/taxane usage (same drugs in each arm)** (χ^2_4 = 3.7; p > 0.1; NS)							
98D BIG 02−98	(3A75;3D100 v 4A50D75) q3; 3CMF q4	A1.5 D1.3	30/959	29/960	−1·0	14·1	
99G NSABP B−30 USA	(4AC;4D100 v 4ADC†) q3	A1.2 D1.3	64/1783	77/1784	−7·7	33·8	
00H BCIRG 005 USA	(4AC;4D100 v 6ADC) q3	A1.2 D1.3	70/1649	60/1649	4·9	32·0	
01= HORG CT/01.04 Greece	(4E90;4D75q3 v 6E75D75†) q3	E1.2 D1.0	6/329	10/329	−2·3	3·8	
03K Fudan TAX 619 China §	(4D100;4A60/E75C600 v 6D75A50/E60C500) q3	E1.3 D1.3	*(603 patients)*		*(no data)*		
06& Sanofi−Aventis USA §	(4AC;4D100 v 6ADC)† + Bev15x34 q3	A1.2 D1.3	*(155 patients)*		*(no data)*		
06T BOOG 2007−02 NL	(4AC;4D100 v 6ADC†) q3‡	A1.2 D1.3	3/100	4/102	−0·6	1·7	
**(B1) subtotal with data §**			**173/ 4820 (3·6%)**	**180/ 4824 (3·7%)**	**−6·6**	**85·5**	**0·93 (0·75 – 1·14) reduction 2p > 0·1; NS**
**(B2) Sequential vs concurrent anthracycline/taxane usage (but some difference in drugs between arms)**							
00) Pfizer USA §	[4EC;4(P175/D75) v 8E75(P175/D75)] q3	E1.2 P1.0 / D1.0	*(617 patients)*		*(no data)*		
04F GEICAM 2003−10 Spain	(4EC;4D100†) q3 v (4E90D75†;4Cap2500x14) q3	E1.0 D1.3	17/669	23/715	−2·2	9·8	
**(B2) subtotal with data §**			**17/ 669 (2·5%)**	**23/ 715 (3·2%)**	**−2·2**	**9·8**	**0·80 (0·43 – 1·50) reduction 2p > 0·1; NS**
**(C) Shorter interval between cycles AND sequential anthracycline/taxane usage** (χ^2_2 = 1.4; p > 0.1; NS)							
98Q AGO−1 Germany	[(3E150;3P250)q2† v 4E90P175 q3]‡; 3CMF q4	E2.5 P2.1	0/333	0/335			
98V AGO IDD−ETC Germany	(3E150;3P225;3C2500)q2† v (4EC;4P175) q3	E2.5 P1.9	0/643	0/612			
00X HE 10/00 Greece	[(3E110;3P250)q2† v 4E83P187 q3]; 3CMFq2†	E2.0 P2.0	15/564	17/557	−0·8	7·5	
03L AERO B−03 France *	(4EC;4D or 4D100;4E100C600)q2† v 6E75D75C500 q3	E2.0 D2.0	0/65	2(0/35)			
04= SBG 2004−1 Sweden *	[(4EC;4D) or (4E100C825;4D80)] q2† v 6ADCq3†	E/A1.9 D1.5	2/84	2(2/40)	−1·0	0·7	
04D NSABP B−38 USA	(4AC;4P175)q2† v 6ADCq3†	A1.8 P/D~1.8	47/1634	46/1630	0·3	23·0	
**(C) subtotal**			**64/ 3323 (1·9%)**	**67/ 3284 (2·0%)**	**−1·5**	**31·2**	**0·95 (0·67 – 1·35) reduction 2p > 0·1; NS**
**(D) Same total dose in fewer cycles**							
85A CALGB 8541 USA	(4F600x2A60C600 v 6F400x2A40C400]) q4	A1.5	58/528	86/522	−15·1	34·1	
88T NCC Japan §	(6C130M26F600A26 v 12C65M13F300A13) q4	A2.0	*(202 patients)*		*(no data)*		
94L SWOG 9313 USA	[(4A40x2q3;3C2400q2) v 6A54C1200q3]†	A1.5	0/1564	0/1612			
**(D) subtotal with data §**			**58/ 2092 (2·8%)**	**86/ 2134 (4·0%)**	**−15·1**	**34·1**	**0·64 (0·46 – 0·90) reduction 2p = 0·010**
**Total (A...D)**			**659/ 18623 (3·5%)**	**810/ 18750 (4·3%)**	**−82·7**	**350·4**	**0·790 (0·711 – 0·877) reduction 2p < 0·00001**

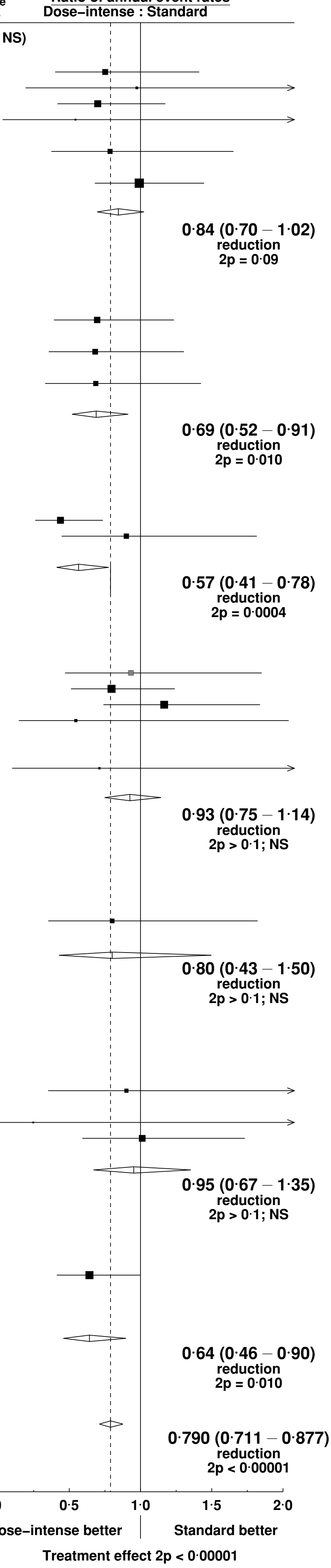

■ 99% or ◇ 95% confidence intervals

Heterogeneity between 7 subtotals: χ^2_6 = 10·4; p > 0·1; NS

Heterogeneity within subtotals: χ^2_{14} = 12·2; p > 0·1; NS

Heterogeneity between 21 trials: χ^2_{20} = 22·5; p > 0·1; NS

Abbreviations: Taxanes: **D** = docetaxel; **P** = paclitaxel. Anthracyclines: **A** = doxorubicin; **E** = epirubicin. Other: **C** = cyclophosphamide; **Cap** = capecitabine; **F** = fluorouracil; **M** = methotrexate; **Bev** = bevacizumab (mg/kg). **AC**=**A**60**C**600; **EC**=**E**90**C**600; **ADC**= **A**50**D**75**C**500; **CMF** = classical CMFd1d8. Chemotherapy doses are in mg/m2. **q2** = 2-weekly, **q3** = 3-weekly, **q4** = 4-weekly. ×14=days 1-14 orally. x2=day 1, day 8. 2p=two-sided p value. * For balance, the 75 control patients in the two 3-way trials count twice in subtotal (C) and in final total of events/patients. NSABP B-38 trial assumes a 2:1 dose equivalence ratio for P to D. ‡ Pre-operative chemotherapy: patients in these trials were analysed as having unknown nodal status. † Primary prophylaxis with colony-stimulating growth factors. § 7 trials with no data do not contribute to subtotals or to the overall total. Semicolon [;] indicates treatment sequence. χ2 tests in section headers are for heterogeneity between trials.

P6: Contralateral breast cancer as first event in trials of dose-intense vs standard schedule chemotherapy

Year code and study name	Comparison	Dose–intensity ratio	Events/Women: Allocated Dose–intense	Events/Women: Allocated Standard	Dose–intense events: Logrank O–E	Dose–intense events: Variance of O–E	Ratio of annual event rates Dose–intense : Standard
**(A1) Shorter interval between cycles (same drugs, doses and number of cycles in each arm)** (χ^2_4 = 1.9; p > 0.1; NS)							
92E GONO MIG1 Italy	$6F_{600}E_{60}C_{600}$ [q2† v q3]	E1.5	13/604	17/610	–0·1	6·3	
92J Pisa/Genoa Italy	[3FEC‡;3($F_{600}E_{60}C_{600}$; CMF)] [q2† v q3]	E1.5	1/73	4/77	–1·3	1·2	
97D CALGB 9741 USA	[($4A_{60}$;$4P_{175}$;$4C_{600}$) or (4AC;$4P_{175}$)][q2† v q3]	A1.5 P1.5	16/1001	24/1004	–4·7	9·6	
97X Bayreuth Germany	$3E_{120}C_{600}$‡ [q2† v q3]	E1.5	0/71	0/69			
03D Royal Marsden UK §	(4AC/4EC)‡ [q2† v q3]	A1.5 / E1.5	*(128 patients)*		*(no data)*		
03Q GIM 2 Italy	[4($E_{90}C_{600}$ ± F_{600});$4P_{175}$] [q2† v q3]	E1.5 P1.5	10/1002	18/1001	–3·3	6·2	
04) CAMS China	$6E_{120}P_{175}$ [q2† v q3]	E1.5 P1.5	0/50	0/51			
05P TACT2 UK	$4E_{100}$ [q2† v q3];4CMF q4/$4Cap_{2500x14}$ q3	E1.5	30/2170	39/2221	–4·4	17·1	
■ **(A1) subtotal with data §**			**70/ 4971 (1·4%)**	**102/ 5033 (2·0%)**	**–13·7**	**40·4**	**0·71 (0·52 – 0·97) reduction 2p = 0·03**
**(A2) Shorter interval between cycles (but additional drugs in control arm)** (χ^2_1 = 0.8; p > 0.1; NS)							
93# EORTC 10921/MA.10	($6E_{120}C_{830}$q2† v $6F_{500x2}E_{60x2}C_{75x14}$q4)‡	E2.0	0/224	1/224	–0·6	0·2	
93W Hamburg Germany §	$4E_{120}C_{600}$q2† v $4E_{90}C_{600}$q3;3CMFq4	E2.0	*(183 patients)*		*(no data)*		
99Y GEPARDUO Germany	[$4A_{50}D_{75}$q2† v (4AC;$4D_{100}$)q3]‡	A1.3 D1.1	0/454	0/456			
00Y GASG Germany §	($4E_{120}$†;$4P_{175}$)q2 v (4EC;$4P_{175}$)q3	E2.0 P1.5	*(884 patients)*		*(no data)*		
07B SBG PANTHER Sweden	($4E_{100}C_{825}$;$4D_{80}$)q2† v ($3F_{500}E_{100}C_{500}$;$3D_{100}$)q3†	E1.5 D1.2	4/1006	8/1008	–1·6	2·9	
▪ **(A2) subtotal with data §**			**4/ 1684 (0·2%)**	**9/ 1688 (0·5%)**	**–2·1**	**3·1**	**0·51 (0·17 – 1·53) reduction 2p > 0·1; NS**
**(A3) Shorter interval between cycles (plus additional treatment in dose–dense arm)**							
00F MA.21 Canada	($6E_{120}C_{830}$q2† v 4AC q3); $4P_{175}$q3	E/A2.0	22/701	18/702	0·0	9·5	
02C PREPARE/GermanBG 49	[($3E_{150}$;$3P_{225}$)q2†;3CMF q4 v (4EC;$4P_{175}$)q3†]‡	E2.5 P1.9	0/363	0/370			
▪ **(A3) subtotal**			**22/ 1064 (2·1%)**	**18/ 1072 (1·7%)**	**0·0**	**9·5**	**1·00 (0·53 – 1·90) increase 2p > 0·1; NS**
**(B1) Sequential vs concurrent anthracycline/taxane usage (same drugs in each arm)** (χ^2_4 = 1.8; p > 0.1; NS)							
98D BIG 02–98	($3A_{75}$;$3D_{100}$ v $4A_{50}D_{75}$) q3; 3CMF q4	A1.5 D1.3	3/959	9/960	–3·2	2·9	
99G NSABP B–30 USA	(4AC;$4D_{100}$ v 4ADC†) q3	A1.2 D1.3	30/1783	44/1784	–8·3	18·2	
00H BCIRG 005 USA	(4AC;$4D_{100}$ v 6ADC) q3	A1.2 D1.3	23/1649	30/1649	–3·7	13·1	
01= HORG CT/01.04 Greece	($4E_{90}$;$4D_{75}$q3 v $6E_{75}D_{75}$†) q3	E1.2 D1.0	1/329	1/329	–0·2	0·5	
03K Fudan TAX 619 China §	($4D_{100}$;$4A_{60}$/$E_{75}C_{600}$ v $6D_{75}A_{50}$/$E_{60}C_{500}$) q3	E1.3 D1.3	*(603 patients)*		*(no data)*		
06& Sanofi–Aventis USA §	(4AC;$4D_{100}$ v 6ADC)† + Bev_{15x34} q3	A1.2 D1.3	*(155 patients)*		*(no data)*		
06T BOOG 2007–02 NL	(4AC;$4D_{100}$ v 6ADC†) q3‡	A1.2 D1.3	2/100	2/102	–0·0	1·0	
■ **(B1) subtotal with data §**			**59/ 4820 (1·2%)**	**86/ 4824 (1·8%)**	**–15·3**	**35·8**	**0·65 (0·47 – 0·90) reduction 2p = 0·01**
**(B2) Sequential vs concurrent anthracycline/taxane usage (but some difference in drugs between arms)**							
00) Pfizer USA §	[4EC;4(P_{175}/D_{75}) v $8E_{75}$(P_{175}/D_{75})] q3	E1.2 P1.0 / D1.0	*(617 patients)*		*(no data)*		
04F GEICAM 2003–10 Spain	(4EC;$4D_{100}$†) q3 v ($4E_{90}D_{75}$†;$4Cap_{2500x14}$) q3	E1.0 D1.3	9/669	14/715	–2·1	5·7	
▪ **(B2) subtotal with data §**			**9/ 669 (1·3%)**	**14/ 715 (2·0%)**	**–2·1**	**5·7**	**0·69 (0·30 – 1·56) reduction 2p > 0·1; NS**
**(C) Shorter interval between cycles AND sequential anthracycline/taxane usage** (χ^2_3 = 0.4; p > 0.1; NS)							
98Q AGO–1 Germany	[($3E_{150}$;$3P_{250}$)q2† v $4E_{90}P_{175}$ q3]‡; 3CMF q4	E2.5 P2.1	0/333	0/335			
98V AGO IDD–ETC Germany	($3E_{150}$;$3P_{225}$;$3C_{2500}$)q2† v (4EC;$4P_{175}$) q3	E2.5 P1.9	13/643	16/612	–2·5	7·2	
00X HE 10/00 Greece	[($3E_{110}$;$3P_{250}$)q2† v $4E_{83}P_{187}$ q3]; 3CMFq2†	E2.0 P2.0	5/564	6/557	–0·4	2·5	
03L AERO B–03 France *	(4EC;4D or $4D_{100}$;$4E_{100}C_{600}$)q2† v $6E_{75}D_{75}C_{500}$ q3	E2.0 D2.0	0/65	2(0/35)			
04= SBG 2004–1 Sweden *	[(4EC;4D) or ($4E_{100}C_{825}$;$4D_{80}$)] q2† v 6ADCq3†	E/A1.9 D1.5	2/84	2(1/40)	–0·0	0·6	
04D NSABP B–38 USA	(4AC;$4P_{175}$)q2† v 6ADCq3†	A1.8 P/D~1.8	23/1634	24/1630	–0·5	11·6	
■ **(C) subtotal**			**43/ 3323 (1·3%)**	**48/ 3284 (1·5%)**	**–3·3**	**21·9**	**0·86 (0·56 – 1·31) reduction 2p > 0·1; NS**
**(D) Same total dose in fewer cycles**							
85A CALGB 8541 USA	($4F_{600x2}A_{60}C_{600}$ v $6F_{400x2}A_{40}C_{400}$]) q4	A1.5	11/528	7/522	0·6	3·9	
88T NCC Japan §	($6C_{130}M_{26}F_{600}A_{26}$ v $12C_{65}M_{13}F_{300}A_{13}$) q4	A2.0	*(202 patients)*		*(no data)*		
94L SWOG 9313 USA	[($4A_{40x2}$q3;$3C_{2400}$q2) v $6A_{54}C_{1200}$q3]†	A1.5	20/1564	16/1612	2·1	8·9	
▪ **(D) subtotal with data §**			**31/ 2092 (1·5%)**	**23/ 2134 (1·1%)**	**2·7**	**12·9**	**1·23 (0·71 – 2·13) increase 2p > 0·1; NS**
■ **Total (A...D)**			**238/ 18623 (1·3%)**	**300/ 18750 (1·6%)**	**–34·0**	**129·2**	**0·769 (0·647 – 0·914) reduction 2p = 0·003**

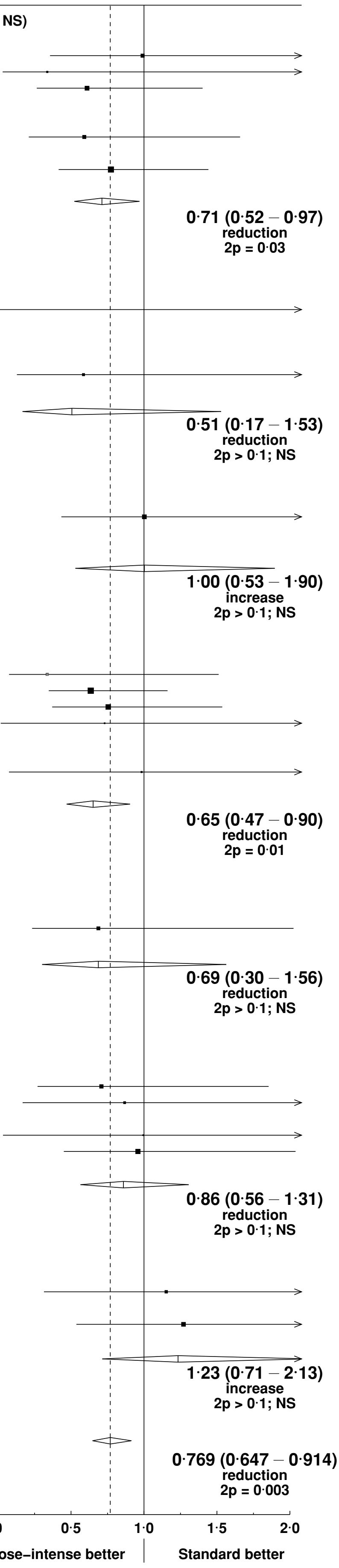

-■- 99% or <> 95% confidence intervals

Heterogeneity between 7 subtotals: χ^2_6 = 5·7; p > 0·1; NS

Heterogeneity within subtotals: χ^2_{13} = 4·9; p > 0·1; NS

Heterogeneity between 20 trials: χ^2_{19} = 10·6; p > 0·1; NS

Abbreviations: Taxanes: **D** = docetaxel; **P** = paclitaxel. Anthracyclines: **A** = doxorubicin; **E** = epirubicin. Other: **C** = cyclophosphamide; **Cap** = capecitabine; **F** = fluorouracil; **M** = methotrexate; **Bev** = bevacizumab (mg/kg). **AC=A60C600; EC=E90C600; ADC= A50D75C500; CMF** = classical CMFd1d8. Chemotherapy doses are in mg/m2. **q2** = 2-weekly, **q3** = 3-weekly, **q4** = 4-weekly. ×14=days 1-14 orally. x2=day 1, day 8. 2p=two-sided p value. * For balance, the 75 control patients in the two 3-way trials count twice in subtotal (C) and in final total of events/patients. NSABP B-38 trial assumes a 2:1 dose equivalence ratio for P to D. ‡ Pre-operative chemotherapy: patients in these trials were analysed as having unknown nodal status. † Primary prophylaxis with colony-stimulating growth factors. § 7 trials with no data do not contribute to subtotals or to the overall total. Semicolon [;] indicates treatment sequence. χ2 tests in section headers are for heterogeneity between trials.

P7: Breast cancer mortality in trials of dose-intense vs standard schedule chemotherapy

Year code	Study name	Comparison	Dose–intensity ratio	Deaths/Women: Allocated Dose–intense	Deaths/Women: Allocated Standard	Dose–intense deaths: Logrank O–E	Dose–intense deaths: Variance of O–E	Ratio of annual death rates Dose–intense : Standard
**(A1) Shorter interval between cycles (same drugs, doses and number of cycles in each arm) (χ^2_6 = 17.8; p = 0.007)**								
92E	GONO MIG1 ITaly	6F$_{600}$E$_{60}$C$_{600}$ [q2† v q3]	E1.5	93/604	108/610	–5·6	45·3	
92J	Pisa/Genoa Italy	[3FEC‡;3(F$_{600}$E$_{60}$C$_{600}$; CMF)] [q2† v q3]	E1.5	23/73	23/77	–0·6	9·7	
97D	CALGB 9741 USA	[(4A$_{60}$;4P$_{175}$;4C$_{600}$) or (4AC;4P$_{175}$)][q2† v q3]	A1.5 P1.5	216/1001	261/1004	–24·4	108·8	
97X	Bayreuth Germany	3E$_{120}$C$_{600}$‡ [q2† v q3]	E1.5	3/71	0/69	1·4	0·7	
03D	Royal Marsden UK §	(4AC/4EC)‡ [q2† v q3]	A1.5 / E1.5	*(128 patients)*		*(no data)*		
03Q	GIM 2 Italy	[4(E$_{90}$C$_{600}$ ± F$_{600}$);4P$_{175}$] [q2† v q3]	E1.5 P1.5	92/1002	145/1001	–27·4	52·8	
04)	CAMS China	6E$_{120}$P$_{175}$ [q2† v q3]	E1.5 P1.5	1/50	5/51	–1·5	1·0	
05P	TACT2 UK	4E$_{100}$ [q2† v q3];4CMF q4/4Cap$_{2500x14}$ q3	E1.5	257/2170	255/2221	6·7	121·6	
	**(A1) subtotal with data §**			**685/ 4971 (13·8%)**	**797/ 5033 (15·8%)**	**–51·4**	**339·9**	**0·86 (0·77 – 0·96) reduction 2p = 0·005**
**(A2) Shorter interval between cycles (but additional drugs in control arm) (χ^2_2 = 1.6; p > 0.1; NS)**								
93#	EORTC 10921/MA.10	(6E$_{120}$C$_{830}$q2† v 6F$_{500x2}$E$_{60x2}$C$_{75x14}$q4)‡	E2.0	112/224	107/224	3·4	46·1	
93W	Hamburg Germany §	4E$_{120}$C$_{600}$q2† v 4E$_{90}$C$_{600}$q3;3CMFq4	E2.0	*(183 patients)*		*(no data)*		
99Y	GEPARDUO Germany	[4A$_{50}$D$_{75}$q2† v (4AC;4D$_{100}$)q3]‡	A1.3 D1.1	54/454	55/456	–0·9	24·8	
00Y	GASG Germany §	(4E$_{120}$†;4P$_{175}$)q2 v (4EC;4P$_{175}$)q3	E2.0 P1.5	*(884 patients)*		*(no data)*		
07B	SBG PANTHER Sweden	(4E$_{100}$C$_{825}$;4D$_{80}$)q2† v (3F$_{500}$E$_{100}$C$_{500}$;3D$_{100}$)q3†	E1.5 D1.2	73/1006	93/1008	–7·9	39·4	
	**(A2) subtotal with data §**			**239/ 1684 (14·2%)**	**255/ 1688 (15·1%)**	**–5·4**	**110·4**	**0·95 (0·79 – 1·15) reduction 2p > 0·1; NS**
**(A3) Shorter interval between cycles (plus additional treatment in dose–dense arm) (χ^2_1 = 1.6; 2p > 0.1; NS)**								
00F	MA.21 Canada	(6E$_{120}$C$_{830}$q2† v 4AC q3); 4P$_{175}$q3	E/A2.0	104/701	146/702	–22·2	59·0	
02C	PREPARE/GermanBG 49	[(3E$_{150}$;3P$_{225}$)q2†;3CMF q4 v (4EC;4P$_{175}$)q3†]‡	E2.5 P1.9	72/363	85/370	–4·2	36·7	
	**(A3) subtotal**			**176/ 1064 (16·5%)**	**231/ 1072 (21·5%)**	**–26·4**	**95·7**	**0·76 (0·62 – 0·93) reduction 2p = 0·007**
**(B1) Sequential vs concurrent anthracycline/taxane usage (same drugs in each arm) (χ^2_4 = 7.8; p = 0.10)**								
98D	BIG 02–98	(3A$_{75}$;3D$_{100}$ v 4A$_{50}$D$_{75}$) q3; 3CMF q4	A1.5 D1.3	112/959	137/960	–15·5	58·8	
99G	NSABP B–30 USA	(4AC;4D$_{100}$ v 4ADC†) q3	A1.2 D1.3	213/1783	238/1784	–14·3	106·0	
00H	BCIRG 005 USA	(4AC;4D$_{100}$ v 6ADC) q3	A1.2 D1.3	299/1649	298/1649	–0·5	142·3	
01=	HORG CT/01.04 Greece	(4E$_{90}$;4D$_{75}$q3 v 6E$_{75}$D$_{75}$†) q3	E1.2 D1.0	11/329	17/329	–2·8	6·8	
03K	Fudan TAX 619 China §	(4D$_{100}$;4A$_{60}$/E$_{75}$C$_{600}$ v 6D$_{75}$A$_{50}$/E$_{60}$C$_{500}$) q3	E1.3 D1.3	*(603 patients)*		*(no data)*		
06&	Sanofi–Aventis USA §	(4AC;4D$_{100}$ v 6ADC)† + Bev$_{15x34}$ q3	A1.2 D1.3	*(155 patients)*		*(no data)*		
06T	BOOG 2007–02 NL	(4AC;4D$_{100}$ v 6ADC†) q3‡	A1.2 D1.3	16/100	29/102	–7·7	10·2	
	**(B1) subtotal with data §**			**651/ 4820 (13·5%)**	**719/ 4824 (14·9%)**	**–40·8**	**324·1**	**0·88 (0·79 – 0·98) reduction 2p = 0·02**
**(B2) Sequential vs concurrent anthracycline/taxane usage (but some difference in drugs between arms)**								
00)	Pfizer USA §	[4EC;4(P$_{175}$/D$_{75}$) v 8E$_{75}$(P$_{175}$/D$_{75}$)] q3	E1.2 P1.0 / D1.0	*(617 patients)*		*(no data)*		
04F	GEICAM 2003–10 Spain	(4EC;4D$_{100}$†) q3 v (4E$_{90}$D$_{75}$†;4Cap$_{2500x14}$) q3	E1.0 D1.3	61/669	71/715	–0·3	31·2	
	**(B2) subtotal with data §**			**61/ 669 (9·1%)**	**71/ 715 (9·9%)**	**–0·3**	**31·2**	**0·99 (0·70 – 1·41) reduction 2p > 0·1; NS**
**(C) Shorter interval between cycles AND sequential anthracycline/taxane usage (χ^2_5 = 10.2; p = 0.07)**								
98Q	AGO–1 Germany	[(3E$_{150}$;3P$_{250}$)q2† v 4E$_{90}$P$_{175}$ q3]‡; 3CMF q4	E2.5 P2.1	44/333	59/335	–8·5	23·6	
98V	AGO IDD–ETC Germany	(3E$_{150}$;3P$_{225}$;3C$_{2500}$)q2† v (4EC;4P$_{175}$) q3	E2.5 P1.9	201/643	245/612	–35·2	104·4	
00X	HE 10/00 Greece	[(3E$_{110}$;3P$_{250}$)q2† v 4E$_{83}$P$_{187}$ q3]; 3CMFq2†	E2.0 P2.0	110/564	126/557	–9·5	53·3	
03L	AERO B–03 France *	(4EC;4D or 4D$_{100}$;4E$_{100}$C$_{600}$)q2† v 6E$_{75}$D$_{75}$C$_{500}$ q3	E2.0 D2.0	0/65	2(1/35)	–0·8	0·2	
04=	SBG 2004–1 Sweden *	[(4EC;4D) or (4E$_{100}$C$_{825}$;4D$_{80}$)] q2† v 6ADCq3†	E/A1.9 D1.5	17/84	2(13/40)	–3·4	6·0	
04D	NSABP B–38 USA	(4AC;4P$_{175}$)q2† v 6ADCq3†	A1.8 P/D~1.8	186/1634	183/1630	1·5	87·8	
	**(C) subtotal**			**558/ 3323 (16·8%)**	**641/ 3284 (19·5%)**	**–55·8**	**275·3**	**0·82 (0·73 – 0·92) reduction 2p = 0·0008**
**(D) Same total dose in fewer cycles (χ^2_1 = 0.5; 2p > 0.1; NS)**								
85A	CALGB 8541 USA	(4F$_{600x2}$A$_{60}$C$_{600}$ v 6F$_{400x2}$A$_{40}$C$_{400}$]) q4	A1.5	225/528	232/522	–2·5	103·8	
88T	NCC Japan §	(6C$_{130}$M$_{26}$F$_{600}$A$_{26}$ v 12C$_{65}$M$_{13}$F$_{300}$A$_{13}$) q4	A2.0	*(202 patients)*		*(no data)*		
94L	SWOG 9313 USA	[(4A$_{40x2}$q3;3C$_{2400}$q2) v 6A$_{54}$C$_{1200}$q3]†	A1.5	295/1564	340/1612	–17·6	151·1	
	**(D) subtotal with data §**			**520/ 2092 (24·9%)**	**572/ 2134 (26·8%)**	**–20·0**	**254·9**	**0·92 (0·82 – 1·05) reduction 2p > 0·1; NS**
	**Total (A...D)**			**2890/ 18623 (15·5%)**	**3286/ 18750 (17·5%)**	**–200·1**	**1431·5**	**0·870 (0·826 – 0·916) reduction 2p < 0·00001**

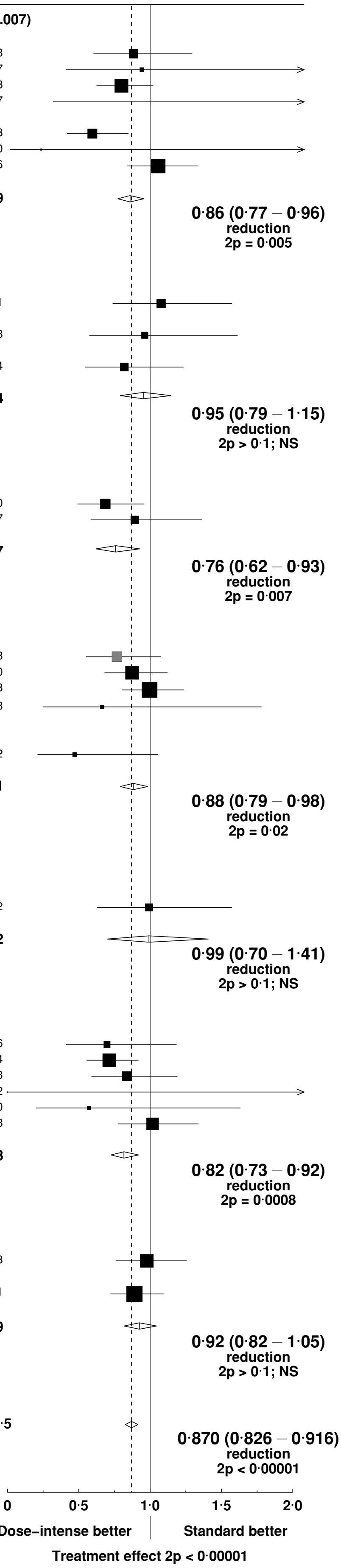

99% or 95% confidence intervals

Heterogeneity between 7 subtotals: χ^2_6 = 5·4; p > 0·1; NS

Heterogeneity within subtotals: χ^2_{19} = 39·5; p = 0·004

Heterogeneity between 26 trials: χ^2_{25} = 44·8; p = 0·009

Abbreviations: Taxanes: **D** = docetaxel**; P** = paclitaxel. Anthracyclines: **A** = doxorubicin; **E** = epirubicin. Other: **C** = cyclophosphamide; **Cap** = capecitabine; **F** = fluorouracil; **M** = methotrexate; **Bev** = bevacizumab (mg/kg). **AC=A60C600; EC=E90C600; ADC= A50D75C500; CMF** = classical CMFd1d8. Chemotherapy doses are in mg/m2. **q2** = 2-weekly, **q3** = 3-weekly, **q4** = 4-weekly. ×14=days 1-14 orally. x2=day 1, day 8. 2p=two-sided p value. * For balance, the 75 control patients in the two 3-way trials count twice in subtotal (C) and in final total of events/patients. NSABP B-38 trial assumes a 2:1 dose equivalence ratio for P to D. ‡ Pre-operative chemotherapy: patients in these trials were analysed as having unknown nodal status. † Primary prophylaxis with colony-stimulating growth factors. § 7 trials with no data do not contribute to subtotals or to the overall total. Semicolon [;] indicates treatment sequence. χ2 tests in section headers are for heterogeneity between trials.

P8: Death without recurrence in year 0 in trials of dose-intense vs standard schedule chemotherapy

Year code and study name	Comparison	Dose–intensity ratio	Deaths/Women: Allocated Dose–intense	Deaths/Women: Allocated Standard	Dose–intense deaths: Logrank O–E	Dose–intense deaths: Variance of O–E	Ratio of annual death rates Dose–intense : Standard
**(A1) Shorter interval between cycles (same drugs, doses and number of cycles in each arm)** (χ^2_4 = 6.8; p > 0.1; NS)							
92E GONO MIG1 Italy	6F600E60C600 [q2† v q3]	E1.5	2/604	0/610	1·1	0·5	
92J Pisa/Genoa Italy	[3FEC‡;3(F600E60C600; CMF)] [q2† v q3]	E1.5	0/73	2/77	−1·1	0·5	
97D CALGB 9741 USA	[(4A60;4P175;4C600) or (4AC;4P175)][q2† v q3]	A1.5 P1.5	4/1001	2/1004	1·0	1·5	
97X Bayreuth Germany	3E120C600‡ [q2† v q3]	E1.5	2/71	0/69	0·9	0·5	
03D Royal Marsden UK §	(4AC/4EC)‡ [q2† v q3]	A1.5 / E1.5	*(128 patients)*		*(no data)*		
03Q GIM 2 Italy	[4(E90C600 ± F600);4P175] [q2† v q3]	E1.5 P1.5	0/1002	0/1001			
04) CAMS China	6E120P175 [q2† v q3]	E1.5 P1.5	0/50	0/51			
05P TACT2 UK	4E100 [q2† v q3];4CMF q4/4Cap2500x14 q3	E1.5	10/2170	9/2221	0·2	4·7	
**(A1) subtotal with data §**			**18/ 4971 (0·4%)**	**13/ 5033 (0·3%)**	**2·1**	**7·6**	**1·31 (0·64 – 2·67) increase 2p > 0·1; NS**
**(A2) Shorter interval between cycles (but additional drugs in control arm)** (χ^2_2 = 3.8; p > 0.1; NS)							
93# EORTC 10921/MA.10	(6E120C830q2† v 6F500x2E60x2C75x14q4)‡	E2.0	1/224	3/224	−1·6	0·7	
93W Hamburg Germany §	4E120C600q2† v 4E90C600q3;3CMFq4	E2.0	*(183 patients)*		*(no data)*		
99Y GEPARDUO Germany	[4A50D75q2† v (4AC;4D100)q3]‡	A1.3 D1.1	5/454	3/456	0·8	1·9	
00Y GASG Germany §	(4E120†;4P175)q2 v (4EC;4P175)q3	E2.0 P1.5	*(884 patients)*		*(no data)*		
07B SBG PANTHER Sweden	(4E100C825;4D80)q2† v (3F500E100C500;3D100)q3†	E1.5 D1.2	1/1006	3/1008	−1·1	1·0	
**(A2) subtotal with data §**			**7/ 1684 (0·4%)**	**9/ 1688 (0·5%)**	**−1·9**	**3·7**	**0·59 (0·21 – 1·65) reduction 2p > 0·1; NS**
**(A3) Shorter interval between cycles (plus additional treatment in dose–dense arm)**							
00F MA.21 Canada	(6E120C830q2† v 4AC q3); 4P175q3	E/A2.0	1/701	0/702	0·5	0·2	
02C PREPARE/GermanBG 49	[(3E150;3P225)q2†;3CMF q4 v (4EC;4P175)q3†]‡	E2.5 P1.9	0/363	0/370			
**(A3) subtotal**			**1/ 1064 (0·1%)**	**0/ 1072 (0·0%)**	**0·5**	**0·2**	**7·69 (0·15 – 387·58) increase 2p > 0·1; NS**
**(B1) Sequential vs concurrent anthracycline/taxane usage (same drugs in each arm)** (χ^2_2 = 1.7; p > 0.1; NS)							
98D BIG 02−98	(3A75;3D100 v 4A50D75) q3; 3CMF q4	A1.5 D1.3	1/959	2/960	−0·2	0·7	
99G NSABP B−30 USA	(4AC;4D100 v 4ADC†) q3	A1.2 D1.3	4/1783	16/1784	−5·8	4·9	
00H BCIRG 005 USA	(4AC;4D100 v 6ADC) q3	A1.2 D1.3	4/1649	5/1649	−0·4	2·2	
01= HORG CT/01.04 Greece	(4E90;4D75q3 v 6E75D75†) q3	E1.2 D1.0	0/329	0/329			
03K Fudan TAX 619 China §	(4D100;4A60/E75C600 v 6D75A50/E60C500) q3	E1.3 D1.3	*(603 patients)*		*(no data)*		
06& Sanofi−Aventis USA §	(4AC;4D100 v 6ADC)† + Bev15x34 q3	A1.2 D1.3	*(155 patients)*		*(no data)*		
06T BOOG 2007−02 NL	(4AC;4D100 v 6ADC†) q3‡	A1.2 D1.3	0/100	0/102			
**(B1) subtotal with data §**			**9/ 4820 (0·2%)**	**23/ 4824 (0·5%)**	**−6·5**	**7·9**	**0·44 (0·22 – 0·88) reduction 2p = 0·02**
**(B2) Sequential vs concurrent anthracycline/taxane usage (but some difference in drugs between arms)**							
00) Pfizer USA §	[4EC;4(P175/D75) v 8E75(P175/D75)] q3	E1.2 P1.0 / D1.0	*(617 patients)*		*(no data)*		
04F GEICAM 2003−10 Spain	(4EC;4D100†) q3 v (4E90D75†;4Cap2500x14) q3	E1.0 D1.3	1/669	2/715	−0·6	0·7	
**(B2) subtotal with data §**			**1/ 669 (0·1%)**	**2/ 715 (0·3%)**	**−0·6**	**0·7**	**0·43 (0·04 – 4·17) reduction 2p > 0·1; NS**
**(C) Shorter interval between cycles AND sequential anthracycline/taxane usage** (χ^2_2 = 4.4; p > 0.1; NS)							
98Q AGO−1 Germany	[(3E150;3P250)q2† v 4E90P175 q3]‡; 3CMF q4	E2.5 P2.1	3/333	2/335	0·2	1·2	
98V AGO IDD−ETC Germany	(3E150;3P225;3C2500)q2† v (4EC;4P175) q3	E2.5 P1.9	0/643	0/612			
00X HE 10/00 Greece	[(3E110;3P250)q2† v 4E83P187 q3]; 3CMFq2†	E2.0 P2.0	4/564	1/557	1·4	1·2	
03L AERO B−03 France *	(4EC;4D or 4D100;4E100C600)q2† v 6E75D75C500 q3	E2.0 D2.0	0/65	2(0/35)			
04= SBG 2004−1 Sweden *	[(4EC;4D) or (4E100C825;4D80)] q2† v 6ADCq3†	E/A1.9 D1.5	0/84	2(0/40)			
04D NSABP B−38 USA	(4AC;4P175)q2† v 6ADCq3†	A1.8 P/D~1.8	6/1634	14/1630	−4·3	4·9	
**(C) subtotal**			**13/ 3323 (0·4%)**	**17/ 3284 (0·5%)**	**−2·7**	**7·3**	**0·69 (0·33 – 1·43) reduction 2p > 0·1; NS**
**(D) Same total dose in fewer cycles** (χ^2_1 = 1.1; 2p > 0.1; NS)							
85A CALGB 8541 USA	(4F600x2A60C600 v 6F400x2A40C400]) q4	A1.5	0/528	2/522	−0·9	0·5	
88T NCC Japan §	(6C130M26F600A26 v 12C65M13F300A13) q4	A2.0	*(202 patients)*		*(no data)*		
94L SWOG 9313 USA	[(4A40x2q3;3C2400q2) v 6A54C1200q3]†	A1.5	4/1564	5/1612	−0·5	2·2	
**(D) subtotal with data §**			**4/ 2092 (0·2%)**	**7/ 2134 (0·3%)**	**−1·5**	**2·7**	**0·58 (0·18 – 1·91) reduction 2p > 0·1; NS**
**Total (A...D)**			**53/ 18623 (0·3%)**	**71/ 18750 (0·4%)**	**−10·6**	**30·1**	**0·703 (0·492 – 1·005) reduction 2p = 0·05**

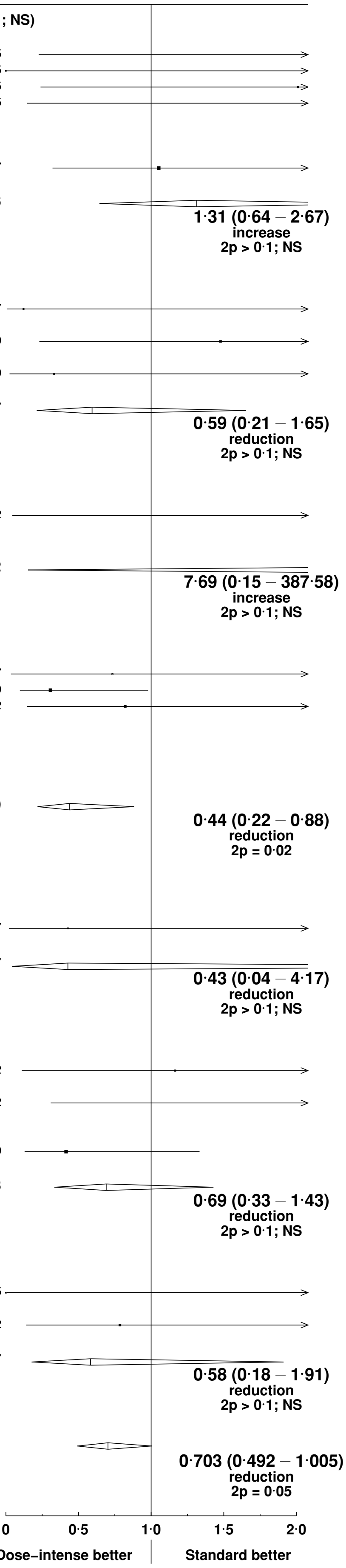

■ 99% or ◇ 95% confidence intervals

Heterogeneity between 7 subtotals: χ^2_6 **= 6·5; p > 0·1; NS**

Heterogeneity within subtotals: χ^2_{11} **= 17·8; p = 0·09**

Heterogeneity between 18 trials: χ^2_{17} **= 24·3; p > 0·1; NS**

Abbreviations: Taxanes: **D** = docetaxel; **P** = paclitaxel. Anthracyclines: **A** = doxorubicin; **E** = epirubicin. Other: **C** = cyclophosphamide; **Cap** = capecitabine; **F** = fluorouracil; **M** = methotrexate; **Bev** = bevacizumab (mg/kg). **AC**=A60C600; **EC**=E90C600; **ADC**= A50D75C500; **CMF** = classical CMFd1d8. Chemotherapy doses are in mg/m2. **q2** = 2-weekly, **q3** = 3-weekly, **q4** = 4-weekly. ×14=days 1-14 orally. x2=day 1, day 8. 2p=two-sided p value. * For balance, the 75 control patients in the two 3-way trials count twice in subtotal (C) and in final total of events/patients. NSABP B-38 trial assumes a 2:1 dose equivalence ratio for P to D. ‡ Pre-operative chemotherapy: patients in these trials were analysed as having unknown nodal status. † Primary prophylaxis with colony-stimulating growth factors. § 7 trials with no data do not contribute to subtotals or to the overall total. Semicolon [;] indicates treatment sequence. χ2 tests in section headers are for heterogeneity between trials.

P9: Death without recurrence in trials of dose-intense vs standard schedule chemotherapy

Year code and study name	Comparison	Dose-intensity ratio	Deaths/Women: Allocated Dose-intense	Deaths/Women: Allocated Standard	Dose-intense deaths: Logrank O−E	Dose-intense deaths: Variance of O−E	Ratio of annual death rates Dose-intense : Standard
**(A1) Shorter interval between cycles (same drugs, doses and number of cycles in each arm)** (χ^2_5 = 6.2; p > 0.1; NS)							
92E GONO MIG1 Italy	6F$_{600}$E$_{60}$C$_{600}$ [q2† v q3]	E1.5	30/604	33/610	−0·2	13·6	
92J Pisa/Genoa Italy	[3FEC‡;3(F$_{600}$E$_{60}$C$_{600}$; CMF)] [q2† v q3]	E1.5	6/73	8/77	−2·2	2·9	
97D CALGB 9741 USA	[(4A$_{60}$;4P$_{175}$;4C$_{600}$) or (4AC;4P$_{175}$)][q2† v q3]	A1.5 P1.5	62/1001	67/1004	−3·5	30·4	
97X Bayreuth Germany	3E$_{120}$C$_{600}$‡ [q2† v q3]	E1.5	2/71	0/69	0·9	0·5	
03D Royal Marsden UK §	(4AC/4EC)‡ [q2† v q3]	A1.5 / E1.5	*(128 patients)*		*(no data)*		
03Q GIM 2 Italy	[4(E$_{90}$C$_{600}$ ± F$_{600}$);4P$_{175}$] [q2† v q3]	E1.5 P1.5	11/1002	4/1001	3·0	3·4	
04) CAMS China	6E$_{120}$P$_{175}$ [q2† v q3]	E1.5 P1.5	0/50	0/51			
05P TACT2 UK	4E$_{100}$ [q2† v q3];4CMF q4/4Cap$_{2500x14}$ q3	E1.5	44/2170	45/2221	−1·1	22·0	
**(A1) subtotal with data §**			**155/ 4971 (3·1%)**	**157/ 5033 (3·1%)**	**−3·2**	**72·8**	**0·96 (0·76 – 1·20) reduction 2p > 0·1; NS**
**(A2) Shorter interval between cycles (but additional drugs in control arm)** (χ^2_2 = 0.1; p > 0.1; NS)							
93# EORTC 10921/MA.10	(6E$_{120}$C$_{830}$q2† v 6F$_{500x2}$E$_{60x2}$C$_{75x14}$q4)‡	E2.0	5/224	4/224	−0·2	2·0	
93W Hamburg Germany §	4E$_{120}$C$_{600}$q2† v 4E$_{90}$C$_{600}$q3;3CMFq4	E2.0	*(183 patients)*		*(no data)*		
99Y GEPARDUO Germany	[4A$_{50}$D$_{75}$q2† v (4AC;4D$_{100}$)q3]‡	A1.3 D1.1	20/454	23/456	−3·2	9·8	
00Y GASG Germany §	(4E$_{120}$†;4P$_{175}$)q2 v (4EC;4P$_{175}$)q3	E2.0 P1.5	*(884 patients)*		*(no data)*		
07B SBG PANTHER Sweden	(4E$_{100}$C$_{825}$;4D$_{80}$)q2† v (3F$_{500}$E$_{100}$C$_{500}$;3D$_{100}$)q3†	E1.5 D1.2	9/1006	11/1008	−1·8	4·9	
**(A2) subtotal with data §**			**34/ 1684 (2·0%)**	**38/ 1688 (2·3%)**	**−5·2**	**16·6**	**0·73 (0·45 – 1·18) reduction 2p > 0·1; NS**
**(A3) Shorter interval between cycles (plus additional treatment in dose−dense arm)** (χ^2_1 = 0.2; 2p > 0.1; NS)							
00F MA.21 Canada	(6E$_{120}$C$_{830}$q2† v 4AC q3); 4P$_{175}$q3	E/A2.0	20/701	13/702	2·1	7·8	
02C PREPARE/GermanBG 49	[(3E$_{150}$;3P$_{225}$)q2†;3CMF q4 v (4EC;4P$_{175}$)q3†]‡	E2.5 P1.9	4/363	3/370	0·9	1·5	
**(A3) subtotal**			**24/ 1064 (2·3%)**	**16/ 1072 (1·5%)**	**3·0**	**9·3**	**1·39 (0·73 – 2·63) increase 2p > 0·1; NS**
**(B1) Sequential vs concurrent anthracycline/taxane usage (same drugs in each arm)** (χ^2_4 = 1.8; p > 0.1; NS)							
98D BIG 02−98	(3A$_{75}$;3D$_{100}$ v 4A$_{50}$D$_{75}$) q3; 3CMF q4	A1.5 D1.3	3/959	3/960	0·1	1·4	
99G NSABP B−30 USA	(4AC;4D$_{100}$ v 4ADC†) q3	A1.2 D1.3	32/1783	45/1784	−6·7	18·7	
00H BCIRG 005 USA	(4AC;4D$_{100}$ v 6ADC) q3	A1.2 D1.3	20/1649	34/1649	−7·0	13·4	
01= HORG CT/01.04 Greece	(4E$_{90}$;4D$_{75}$q3 v 6E$_{75}$D$_{75}$†) q3	E1.2 D1.0	0/329	2/329	−1·0	0·5	
03K Fudan TAX 619 China §	(4D$_{100}$;4A$_{60}$/E$_{75}$C$_{600}$ v 6D$_{75}$A$_{50}$/E$_{60}$C$_{500}$) q3	E1.3 D1.3	*(603 patients)*		*(no data)*		
06& Sanofi−Aventis USA §	(4AC;4D$_{100}$ v 6ADC)† + Bev$_{15x34}$ q3	A1.2 D1.3	*(155 patients)*		*(no data)*		
06T BOOG 2007−02 NL	(4AC;4D$_{100}$ v 6ADC†) q3‡	A1.2 D1.3	1/100	1/102	0·0	0·5	
**(B1) subtotal with data §**			**56/ 4820 (1·2%)**	**85/ 4824 (1·8%)**	**−14·6**	**34·6**	**0·66 (0·47 – 0·91) reduction 2p = 0·01**
**(B2) Sequential vs concurrent anthracycline/taxane usage (but some difference in drugs between arms)**							
00) Pfizer USA §	[4EC;4(P$_{175}$/D$_{75}$) v 8E$_{75}$(P$_{175}$/D$_{75}$)] q3	E1.2 P1.0 / D1.0	*(617 patients)*		*(no data)*		
04F GEICAM 2003−10 Spain	(4EC;4D$_{100}$†) q3 v (4E$_{90}$D$_{75}$†;4Cap$_{2500x14}$) q3	E1.0 D1.3	9/669	12/715	−1·7	5·2	
**(B2) subtotal with data §**			**9/ 669 (1·3%)**	**12/ 715 (1·7%)**	**−1·7**	**5·2**	**0·72 (0·30 – 1·70) reduction 2p > 0·1; NS**
**(C) Shorter interval between cycles AND sequential anthracycline/taxane usage** (χ^2_3 = 3.4; p > 0.1; NS)							
98Q AGO−1 Germany	[(3E$_{150}$;3P$_{250}$)q2† v 4E$_{90}$P$_{175}$ q3]‡; 3CMF q4	E2.5 P2.1	8/333	12/335	−2·9	4·7	
98V AGO IDD−ETC Germany	(3E$_{150}$;3P$_{225}$;3C$_{2500}$)q2† v (4EC;4P$_{175}$) q3	E2.5 P1.9	0/643	0/612			
00X HE 10/00 Greece	[(3E$_{110}$;3P$_{250}$)q2† v 4E$_{83}$P$_{187}$ q3]; 3CMFq2†	E2.0 P2.0	21/564	15/557	2·9	8·8	
03L AERO B−03 France *	(4EC;4D or 4D$_{100}$;4E$_{100}$C$_{600}$)q2† v 6E$_{75}$D$_{75}$C$_{500}$ q3	E2.0 D2.0	0/65	2(0/35)			
04= SBG 2004−1 Sweden *	[(4EC;4D) or (4E$_{100}$C$_{825}$;4D$_{80}$)] q2† v 6ADCq3†	E/A1.9 D1.5	1/84	2(1/40)	−0·5	0·4	
04D NSABP B−38 USA	(4AC;4P$_{175}$)q2† v 6ADCq3†	A1.8 P/D~1.8	46/1634	46/1630	0·5	22·6	
**(C) subtotal**			**76/ 3323 (2·3%)**	**75/ 3284 (2·3%)**	**−0·1**	**36·5**	**1·00 (0·72 – 1·38) reduction 2p > 0·1; NS**
**(D) Same total dose in fewer cycles** (χ^2_1 = 1.8; 2p > 0.1; NS)							
85A CALGB 8541 USA	(4F$_{600x2}$A$_{60}$C$_{600}$ v 6F$_{400x2}$A$_{40}$C$_{400}$]) q4	A1.5	41/528	58/522	−8·9	23·4	
88T NCC Japan §	(6C$_{130}$M$_{26}$F$_{600}$A$_{26}$ v 12C$_{65}$M$_{13}$F$_{300}$A$_{13}$) q4	A2.0	*(202 patients)*		*(no data)*		
94L SWOG 9313 USA	[(4A$_{40x2}$q3;3C$_{2400}$q2) v 6A$_{54}$C$_{1200}$q3]†	A1.5	145/1564	143/1612	−4·0	68·1	
**(D) subtotal with data §**			**186/ 2092 (8·9%)**	**201/ 2134 (9·4%)**	**−12·9**	**91·5**	**0·87 (0·71 – 1·07) reduction 2p > 0·1; NS**
**Total (A...D)**			**540/ 18623 (2·9%)**	**584/ 18750 (3·1%)**	**−34·6**	**266·5**	**0·878 (0·779 – 0·990) reduction 2p = 0·03**

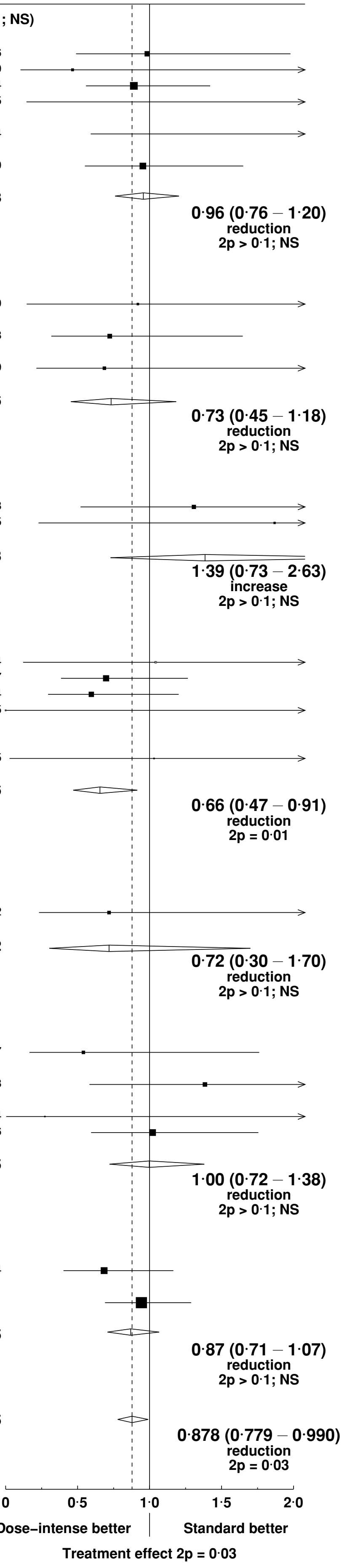

■ 99% or ◇ 95% confidence intervals

Heterogeneity between 7 subtotals: χ^2_6 = 6·8; p > 0·1; NS

Heterogeneity within subtotals: χ^2_{16} = 13·4; p > 0·1; NS

Heterogeneity between 23 trials: χ^2_{22} = 20·2; p > 0·1; NS

Abbreviations: Taxanes: **D** = docetaxel; **P** = paclitaxel. Anthracyclines: **A** = doxorubicin; **E** = epirubicin. Other: **C** = cyclophosphamide; **Cap** = capecitabine; **F** = fluorouracil; **M** = methotrexate; **Bev** = bevacizumab (mg/kg). **AC=A60C600; EC=E90C600; ADC= A50D75C500; CMF** = classical CMFd1d8. Chemotherapy doses are in mg/m2. **q2** = 2-weekly, **q3** = 3-weekly, **q4** = 4-weekly. ×14=days 1-14 orally. x2=day 1, day 8. 2p=two-sided p value. * For balance, the 75 control patients in the two 3-way trials count twice in subtotal (C) and in final total of events/patients. NSABP B-38 trial assumes a 2:1 dose equivalence ratio for P to D. ‡ Pre-operative chemotherapy: patients in these trials were analysed as having unknown nodal status. † Primary prophylaxis with colony-stimulating growth factors. § 7 trials with no data do not contribute to subtotals or to the overall total. Semicolon [;] indicates treatment sequence. χ2 tests in section headers are for heterogeneity between trials.

P10: All-cause mortality in trials of dose-intense vs standard schedule chemotherapy

Year code and study name	Comparison	Dose-intensity ratio	Deaths/Women: Allocated Dose-intense	Deaths/Women: Allocated Standard	Dose-intense deaths: Logrank O−E	Dose-intense deaths: Variance of O−E	Ratio of annual death rates Dose-intense : Standard
**(A1) Shorter interval between cycles (same drugs, doses and number of cycles in each arm)** (χ^2_6 = 16.7; p = 0.01)							
92E GONO MIG1 Italy	6F$_{600}$E$_{60}$C$_{600}$ [q2† v q3]	E1.5	123/604	141/610	−5·8	58·9	
92J Pisa/Genoa Italy	[3FEC‡;3(F$_{600}$E$_{60}$C$_{600}$; CMF)] [q2† v q3]	E1.5	29/73	31/77	−2·8	12·6	
97D CALGB 9741 USA	[(4A$_{60}$;4P$_{175}$;4C$_{600}$) or (4AC;4P$_{175}$)][q2† v q3]	A1.5 P1.5	278/1001	328/1004	−27·9	139·2	
97X Bayreuth Germany	3E$_{120}$C$_{600}$‡ [q2† v q3]	E1.5	5/71	0/69	2·2	1·2	
03D Royal Marsden UK §	(4AC/4EC)‡ [q2† v q3]	A1.5 / E1.5	(128 patients)		(no data)		
03Q GIM 2 Italy	[4(E$_{90}$C$_{600}$ ± F$_{600}$);4P$_{175}$] [q2† v q3]	E1.5 P1.5	103/1002	149/1001	−24·4	56·1	
04) CAMS China	6E$_{120}$P$_{175}$ [q2† v q3]	E1.5 P1.5	1/50	5/51	−1·5	1·0	
05P TACT2 UK	4E$_{100}$ [q2† v q3];4CMF q4/4Cap$_{2500x14}$ q3	E1.5	301/2170	300/2221	5·7	143·6	
**(A1) subtotal with data §**			**840/ 4971 (16·9%)**	**954/ 5033 (19·0%)**	**−54·6**	**412·6**	**0·88 (0·80 – 0·96) reduction 2p = 0·007**
**(A2) Shorter interval between cycles (but additional drugs in control arm)** (χ^2_2 = 2.0; p > 0.1; NS)							
93# EORTC 10921/MA.10	(6E$_{120}$C$_{830}$q2† v 6F$_{500x2}$E$_{60x2}$C$_{75x14}$q4)‡	E2.0	117/224	111/224	3·3	48·1	
93W Hamburg Germany §	4E$_{120}$C$_{600}$q2† v 4E$_{90}$C$_{600}$q3;3CMFq4	E2.0	(183 patients)		(no data)		
99Y GEPARDUO Germany	[4A$_{50}$D$_{75}$q2† v (4AC;4D$_{100}$)q3]‡	A1.3 D1.1	74/454	78/456	−4·1	34·6	
00Y GASG Germany §	(4E$_{120}$†;4P$_{175}$)q2 v (4EC;4P$_{175}$)q3	E2.0 P1.5	(884 patients)		(no data)		
07B SBG PANTHER Sweden	(4E$_{100}$C$_{825}$;4D$_{80}$)q2† v (3F$_{500}$E$_{100}$C$_{500}$;3D$_{100}$)q3†	E1.5 D1.2	82/1006	104/1008	−9·7	44·3	
**(A2) subtotal with data §**			**273/ 1684 (16·2%)**	**293/ 1688 (17·4%)**	**−10·6**	**127·0**	**0·92 (0·77 – 1·10) reduction 2p > 0·1; NS**
**(A3) Shorter interval between cycles (plus additional treatment in dose−dense arm)** (χ^2_1 = 1.1; 2p > 0.1; NS)							
00F MA.21 Canada	(6E$_{120}$C$_{830}$q2† v 4AC q3); 4P$_{175}$q3	E/A2.0	124/701	159/702	−20·1	66·8	
02C PREPARE/GermanBG 49	[(3E$_{150}$;3P$_{225}$)q2†;3CMF q4 v (4EC;4P$_{175}$)q3†]‡	E2.5 P1.9	76/363	88/370	−3·2	38·2	
**(A3) subtotal**			**200/ 1064 (18·8%)**	**247/ 1072 (23·0%)**	**−23·4**	**105·0**	**0·80 (0·66 – 0·97) reduction 2p = 0·02**
**(B1) Sequential vs concurrent anthracycline/taxane usage (same drugs in each arm)** (χ^2_4 = 6.7; p > 0.1; NS)							
98D BIG 02−98	(3A$_{75}$;3D$_{100}$ v 4A$_{50}$D$_{75}$) q3; 3CMF q4	A1.5 D1.3	115/959	140/960	−15·5	60·2	
99G NSABP B−30 USA	(4AC;4D$_{100}$ v 4ADC†) q3	A1.2 D1.3	245/1783	283/1784	−21·0	124·7	
00H BCIRG 005 USA	(4AC;4D$_{100}$ v 6ADC) q3	A1.2 D1.3	319/1649	332/1649	−7·5	155·7	
01= HORG CT/01.04 Greece	(4E$_{90}$;4D$_{75}$q3 v 6E$_{75}$D$_{75}$†) q3	E1.2 D1.0	11/329	19/329	−3·8	7·3	
03K Fudan TAX 619 China §	(4D$_{100}$;4A$_{60}$/E$_{75}$C$_{600}$ v 6D$_{75}$A$_{50}$/E$_{60}$C$_{500}$) q3	E1.3 D1.3	(603 patients)		(no data)		
06& Sanofi−Aventis USA §	(4AC;4D$_{100}$ v 6ADC)† + Bev$_{15x34}$ q3	A1.2 D1.3	(155 patients)		(no data)		
06T BOOG 2007−02 NL	(4AC;4D$_{100}$ v 6ADC†) q3‡	A1.2 D1.3	17/100	30/102	−7·7	10·7	
**(B1) subtotal with data §**			**707/ 4820 (14·7%)**	**804/ 4824 (16·7%)**	**−55·5**	**358·7**	**0·86 (0·77 – 0·95) reduction 2p = 0·003**
**(B2) Sequential vs concurrent anthracycline/taxane usage (but some difference in drugs between arms)**							
00) Pfizer USA §	[4EC;4(P$_{175}$/D$_{75}$) v 8E$_{75}$(P$_{175}$/D$_{75}$)] q3	E1.2 P1.0 / D1.0	(617 patients)		(no data)		
04F GEICAM 2003−10 Spain	(4EC;4D$_{100}$†) q3 v (4E$_{90}$D$_{75}$†;4Cap$_{2500x14}$) q3	E1.0 D1.3	70/669	83/715	−2·0	36·4	
**(B2) subtotal with data §**			**70/ 669 (10·5%)**	**83/ 715 (11·6%)**	**−2·0**	**36·4**	**0·95 (0·68 – 1·31) reduction 2p > 0·1; NS**
**(C) Shorter interval between cycles AND sequential anthracycline/taxane usage** (χ^2_5 = 12.5; p = 0.03)							
98Q AGO−1 Germany	[(3E$_{150}$;3P$_{250}$)q2† v 4E$_{90}$P$_{175}$ q3]‡; 3CMF q4	E2.5 P2.1	52/333	71/335	−11·4	28·4	
98V AGO IDD−ETC Germany	(3E$_{150}$;3P$_{225}$;3C$_{2500}$)q2† v (4EC;4P$_{175}$) q3	E2.5 P1.9	201/643	245/612	−35·2	104·4	
00X HE 10/00 Greece	[(3E$_{110}$;3P$_{250}$)q2† v 4E$_{83}$P$_{187}$ q3]; 3CMFq2†	E2.0 P2.0	131/564	141/557	−6·6	62·2	
03L AERO B−03 France *	(4EC;4D or 4D$_{100}$;4E$_{100}$C$_{600}$)q2† v 6E$_{75}$D$_{75}$C$_{500}$ q3	E2.0 D2.0	0/65	2(1/35)	−0·8	0·2	
04= SBG 2004−1 Sweden *	[(4EC;4D) or (4E$_{100}$C$_{825}$;4D$_{80}$)] q2† v 6ADCq3†	E/A1.9 D1.5	18/84	2(14/40)	−3·9	6·4	
04D NSABP B−38 USA	(4AC;4P$_{175}$)q2† v 6ADCq3†	A1.8 P/D~1.8	232/1634	229/1630	2·0	110·4	
**(C) subtotal**			**634/ 3323 (19·1%)**	**716/ 3284 (21·8%)**	**−55·9**	**311·9**	**0·84 (0·75 – 0·93) reduction 2p = 0·002**
**(D) Same total dose in fewer cycles** (χ^2_1 = 0.0; 2p > 0.1; NS)							
85A CALGB 8541 USA	(4F$_{600x2}$A$_{60}$C$_{600}$ v 6F$_{400x2}$A$_{40}$C$_{400}$]) q4	A1.5	266/528	290/522	−11·4	127·2	
88T NCC Japan §	(6C$_{130}$M$_{26}$F$_{600}$A$_{26}$ v 12C$_{65}$M$_{13}$F$_{300}$A$_{13}$) q4	A2.0	(202 patients)		(no data)		
94L SWOG 9313 USA	[(4A$_{40x2}$q3;3C$_{2400}$q2) v 6A$_{54}$C$_{1200}$q3]†	A1.5	440/1564	483/1612	−21·6	219·2	
**(D) subtotal with data §**			**706/ 2092 (33·7%)**	**773/ 2134 (36·2%)**	**−32·9**	**346·4**	**0·91 (0·82 – 1·01) reduction 2p = 0·08**
**Total (A...D)**			**3430/ 18623 (18·4%)**	**3870/ 18750 (20·6%)**	**−234·7**	**1698·0**	**0·871 (0·830 – 0·913) reduction 2p < 0·00001**

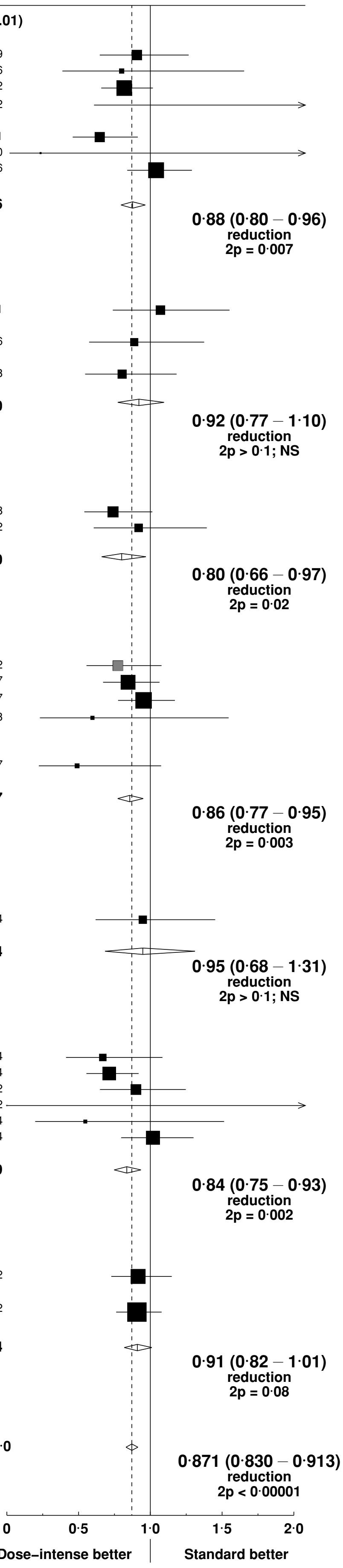

■ 99% or ◇ 95% confidence intervals

Heterogeneity between 7 subtotals: χ^2_6 = 2·7; p > 0·1; NS

Heterogeneity within subtotals: χ^2_{19} = 39·1; p = 0·004

Heterogeneity between 26 trials: χ^2_{25} = 41·8; p = 0·02

Abbreviations: Taxanes: **D** = docetaxel**; P** = paclitaxel. Anthracyclines: **A** = doxorubicin; **E** = epirubicin. Other: **C** = cyclophosphamide; **Cap** = capecitabine; **F** = fluorouracil; **M** = methotrexate; **Bev** = bevacizumab (mg/kg). **AC=A60C600; EC=E90C600; ADC= A50D75C500; CMF** = classical CMFd1d8. Chemotherapy doses are in mg/m2. **q2** = 2-weekly, **q3** = 3-weekly, **q4** = 4-weekly. ×14=days 1-14 orally. x2=day 1, day 8. 2p=two-sided p value. * For balance, the 75 control patients in the two 3-way trials count twice in subtotal (C) and in final total of events/patients. NSABP B-38 trial assumes a 2:1 dose equivalence ratio for P to D. ‡ Pre-operative chemotherapy: patients in these trials were analysed as having unknown nodal status. † Primary prophylaxis with colony-stimulating growth factors. § 7 trials with no data do not contribute to subtotals or to the overall total. Semicolon [;] indicates treatment sequence. χ2 tests in section headers are for heterogeneity between trials.

P11: Trials of dose-dense (2-weekly) chemotherapy vs the same chemotherapy (3-weekly) schedule [A1]: subgroup analyses for recurrence*

Category	Events/Women: Allocated Dose-dense	Events/Women: Allocated Standard	Dose-dense events: Logrank O−E	Dose-dense events: Variance of O−E	Ratio of annual event rates: Ratio (& CI)
**(a) Chemo regimen/no. of dose-dense cycles** (χ^2_2 = 6.8; 2p = 0.03)					
Anth; <=4 cycles	387/2241 (17·3%)	421/2290 (18·4%)	−11·4	190·8	0·94 (0·78 – 1·14)
Anth; 6+ cycles	177/677 (26·1%)	208/687 (30·3%)	−9·5	83·1	0·89 (0·67 – 1·18)
Anth+Tax; 6+ cycles	466/2053 (22·7%)	582/2056 (28·3%)	−70·6	231·2	0·74 (0·62 – 0·87)
**(b) Follow up period, years** (trend χ^2_1 = 1.4; 2p > 0.1; NS)					
0–1	328/4971 (6·6%)	418/5033 (8·3%)	−41·3	164·6	0·78 (0·64 – 0·95)
2–4	452/4490 (10·1%)	522/4458 (11·7%)	−35·2	221·0	0·85 (0·72 – 1·01)
5–9	228/3765 (6·1%)	249/3668 (6·8%)	−15·8	109·8	0·87 (0·68 – 1·11)
10+	22/750 (2·9%)	22/721 (3·1%)	0·9	9·7	
**(c) Site of first recurrence** (χ^2_2 = 1.2; p > 0.1; NS)					
Distant	760/4971 (15·3%)	876/5033 (17·4%)	−60·3	372·6	0·85 (0·74 – 0·97)
Isolated local	200/4971 (4·0%)	233/5033 (4·6%)	−17·5	103·4	0·84 (0·66 – 1·09)
Contralateral	70/4971 (1·4%)	102/5033 (2·0%)	−13·7	40·4	0·71 (0·47 – 1·07)
**(d) Age** (trend χ^2_1 = 0.1; 2p > 0.1: NS)					
Age < 45	300/1270 (23·6%)	357/1264 (28·2%)	−32·0	146·8	0·80 (0·65 – 0·99)
45 – 54	317/1759 (18·0%)	368/1856 (19·8%)	−22·0	156·9	0·87 (0·71 – 1·07)
55 – 69	393/1872 (21·0%)	461/1843 (25·0%)	−36·3	193·9	0·83 (0·69 – 1·00)
70+	20/69 (29·0%)	25/68 (36·8%)	−1·2	7·6	
**(e) Tumour size** (trend χ^2_1 = 1.2; 2p > 0.1: NS)					
1 – 20 mm (T1)	314/2119 (14·8%)	405/2138 (18·9%)	−45·8	170·2	0·76 (0·63 – 0·93)
21 – 50 mm (T2)	578/2338 (24·7%)	633/2359 (26·8%)	−30·6	277·5	0·90 (0·77 – 1·05)
> 50 mm (T3/T4)	87/270 (32·2%)	103/278 (37·1%)	−8·4	37·7	0·80 (0·53 – 1·22)
**(f) Nodal status** (trend χ^2_1 = 0.7; 2p > 0.1: NS)					
N0	169/1240 (13·6%)	184/1277 (14·4%)	−5·7	84·5	0·93 (0·71 – 1·24)
N1–3	412/2349 (17·5%)	487/2337 (20·8%)	−42·3	210·6	0·82 (0·68 – 0·98)
N4+	409/1217 (33·6%)	489/1248 (39·2%)	−38·2	193·0	0·82 (0·68 – 0·99)
**(g) ER/PR status** (χ^2_1 = 0.1; p > 0.1; NS)					
ER− PR any	372/1431 (26·0%)	431/1450 (29·7%)	−34·6	182·3	0·83 (0·68 – 1·00)
ER? PR any	52/263 (19·8%)	73/303 (24·1%)	−6·9	27·7	0·78 (0·48 – 1·27)
ER+ PR+	400/2288 (17·5%)	478/2294 (20·8%)	−41·8	210·5	0·82 (0·69 – 0·98)
ER+ PR−	123/513 (24·0%)	134/509 (26·3%)	−9·3	60·1	0·86 (0·61 – 1·19)
ER+ PR?	83/476 (17·4%)	95/477 (19·9%)	−6·0	41·9	0·87 (0·58 – 1·29)
**(h) HER2 status** (χ^2_1 = 2.7; 2p > 0.1; NS)					
HER2 positive	159/732 (21·7%)	154/720 (21·4%)	3·6	63·5	
HER2 negative	493/2735 (18·0%)	590/2793 (21·1%)	−42·8	247·1	0·84 (0·71 – 0·99)
**(i) Tumour grade** (trend χ^2_1 = 0.5; 2p > 0.1: NS)					
Well-differentiated	16/191 (8·4%)	21/178 (11·8%)	−1·3	8·3	
Moderately	272/1645 (16·5%)	313/1609 (19·5%)	−26·5	138·2	0·83 (0·66 – 1·03)
Poorly	432/1911 (22·6%)	496/2015 (24·6%)	−23·0	216·3	0·90 (0·75 – 1·07)
**(j) Ki67** (trend χ^2_1 = 1.1; p > 0.1: NS)					
Ki67 <10%	68/485 (14·0%)	78/450 (17·3%)	−7·4	29·2	0·78 (0·48 – 1·25)
Ki67 >=10% – <20%	106/645 (16·4%)	137/679 (20·2%)	−12·8	50·1	0·78 (0·54 – 1·12)
Ki67 >=20%	350/1666 (21·0%)	396/1706 (23·2%)	−17·9	162·9	0·90 (0·73 – 1·10)
**(k) Histological type** (χ^2_1 = 0.2; 2p > 0.1; NS)					
Ductal	618/3153 (19·6%)	723/3255 (22·2%)	−41·5	300·6	0·87 (0·75 – 1·01)
Lobular	66/381 (17·3%)	78/366 (21·3%)	−1·4	27·3	
**Total**	**1030/4971 (20·7%)**	**1211/5033 (24·1%)**	**−91·6**	**505·2**	**0·834 (0·765 – 0·910)** 2p = 0·00005

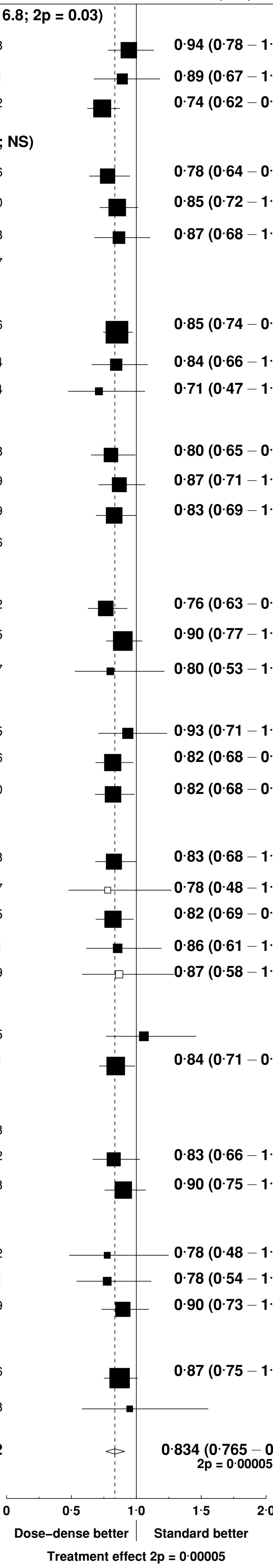

99% or 95% confidence intervals

* Unknowns omitted

P12: 5-year risk of recurrence by ER status in trials of dose-dense (2-weekly) chemotherapy vs the same chemotherapy (3-weekly) schedule [A1]

ER−negative

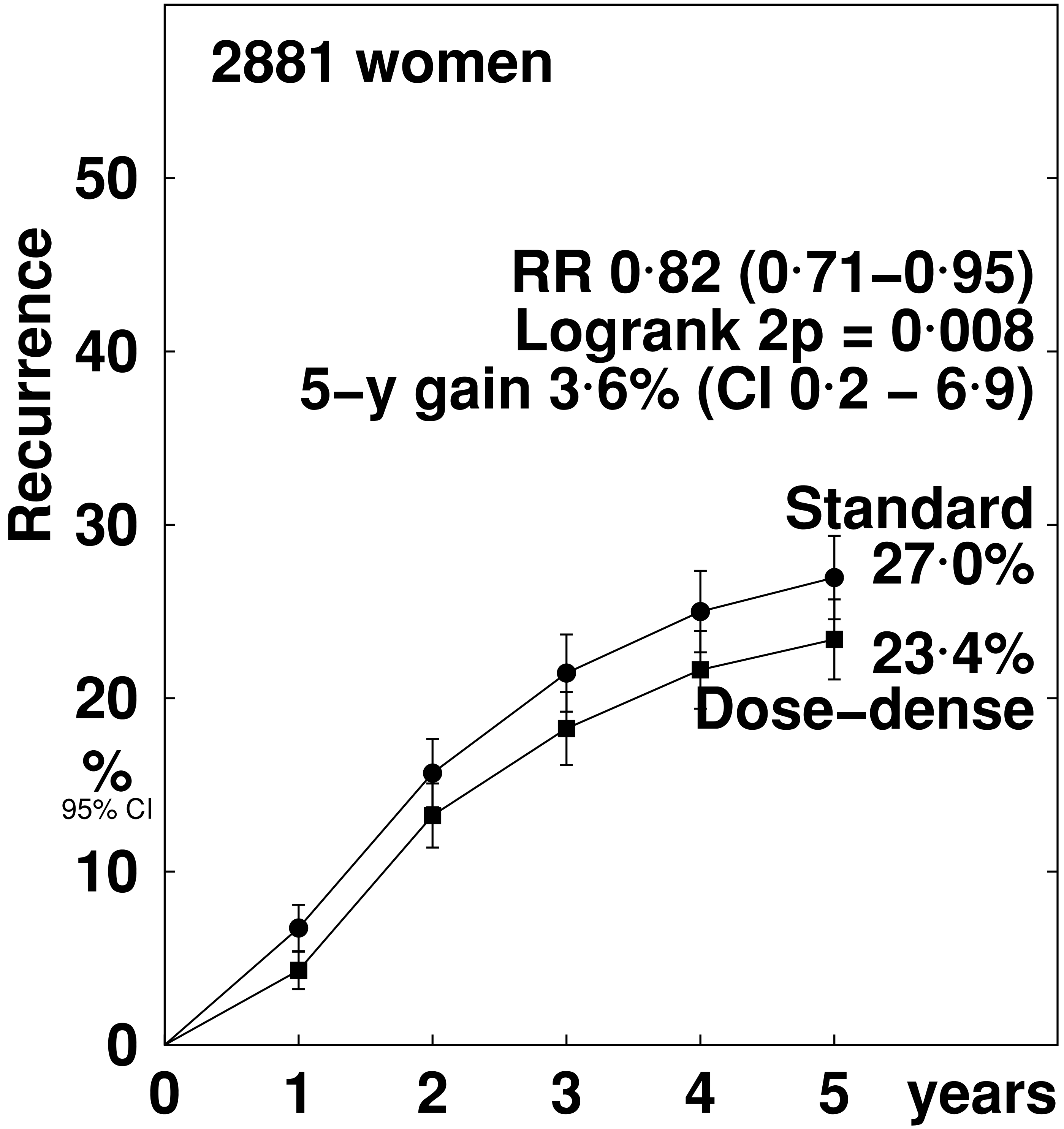

Recurrence rates (% / year) and logrank analyses

Allocation	Years 0 – 1	Years 2 – 4	Year 5+
Dose−dense	7·03 (189 / 2688)	4·27 (140 / 3280)	1·13 (43 / 3819)
Standard	8·40 (223 / 2656)	4·87 (153 / 3139)	1·51 (55 / 3649)
Rate ratio, from	0·82 CI 0·63 − 1·00	0·86 CI 0·64 − 1·08	0·74 CI 0·39 − 1·08
(O−E) / V	−18·8 / 92·5	−10·0 / 68·3	−7·2 / 23·5

ER−positive

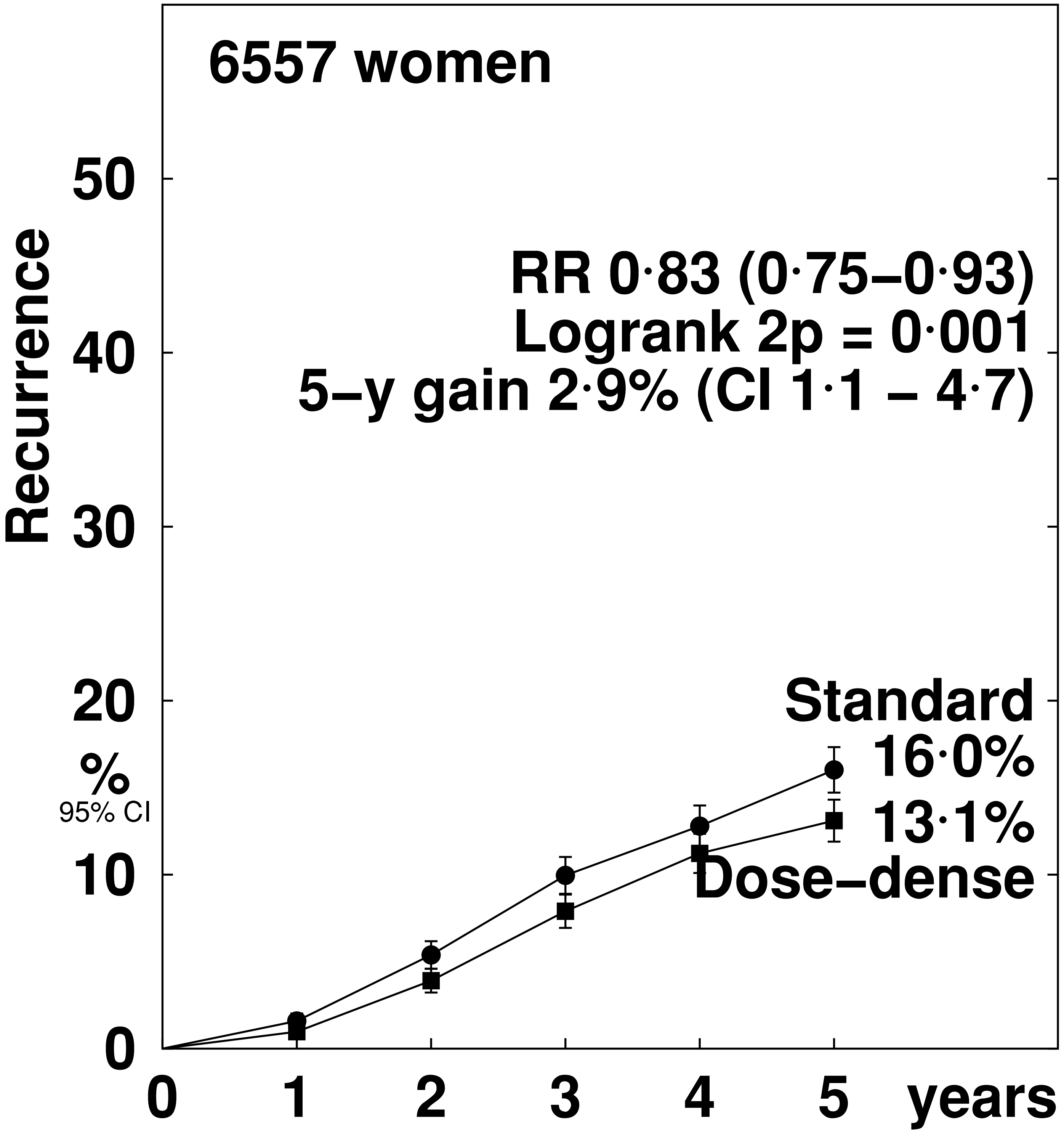

Recurrence rates (% / year) and logrank analyses

Allocation	Years 0 – 1	Years 2 – 4	Year 5+
Dose−dense	2·00 (127 / 6357)	3·41 (286 / 8399)	2·22 (193 / 8710)
Standard	2·73 (172 / 6309)	3·99 (329 / 8246)	2·44 (206 / 8458)
Rate ratio, from	0·72 CI 0·52 − 0·91	0·85 CI 0·70 − 0·99	0·91 CI 0·72 − 1·10
(O−E) / V	−23·9 / 71·9	−24·6 / 146·9	−9·5 / 96·8

P13: All trials of dose-dense (2-weekly) vs standard schedule (3 or 4 weekly) chemotherapy [A1-A3]: subgroup analyses for recurrence*

Category	Events/Women Allocated Dose–dense	Events/Women Allocated Standard	Dose–dense events Logrank O–E	Dose–dense events Variance of O–E	Ratio (& CI)
**(a) Chemo regimen/no. of dose–dense cycles** (χ^2_3 = 4.1; p > 0.1; NS)					
**Anth; <=4 cycles**	387/2241 (17·3%)	421/2290 (18·4%)	−11·4	190·8	0·94 (0·78 – 1·14)
**Anth+Tax; <=4 cycles**	91/454 (20·0%)	104/456 (22·8%)	−7·8	44·8	0·84 (0·57 – 1·24)
**Anth; 6+ cycles**	484/1602 (30·2%)	561/1613 (34·8%)	−41·7	226·6	0·83 (0·70 – 0·99)
**Anth+Tax; 6+ cycles**	706/3422 (20·6%)	867/3434 (25·2%)	−85·3	352·0	0·78 (0·68 – 0·90)
**(b) Follow up period, years** (trend χ^2_1 = 2.9; 2p = 0.09)					
**0–1**	608/7719 (7·9%)	762/7793 (9·8%)	−73·5	298·3	0·78 (0·67 – 0·91)
**2–4**	698/6859 (10·2%)	807/6792 (11·9%)	−55·7	344·6	0·85 (0·74 – 0·98)
**5–9**	333/5423 (6·1%)	355/5268 (6·7%)	−18·2	158·6	0·89 (0·73 – 1·09)
**10+**	29/992 (2·9%)	29/971 (3·0%)	1·5	12·8	
**(c) Site of first recurrence** (χ^2_2 = 5.5; p = 0.06)					
**Distant**	1225/7719 (15·9%)	1370/7793 (17·6%)	−73·0	594·8	0·88 (0·80 – 0·98)
**Isolated local**	347/7719 (4·5%)	454/7793 (5·8%)	−57·3	189·8	0·74 (0·61 – 0·89)
**Contralateral**	96/7719 (1·2%)	129/7793 (1·7%)	−15·9	53·0	0·74 (0·52 – 1·06)
**(d) Age** (trend χ^2_1 = 0.8; 2p > 0.1: NS)					
**Age < 45**	509/2089 (24·4%)	631/2123 (29·7%)	−63·8	255·6	0·78 (0·66 – 0·92)
**45 – 54**	563/2847 (19·8%)	621/2904 (21·4%)	−31·6	269·2	0·89 (0·76 – 1·04)
**55 – 69**	566/2695 (21·0%)	671/2682 (25·0%)	−51·5	278·7	0·83 (0·71 – 0·97)
**70+**	30/87 (34·5%)	30/82 (36·6%)	0·7	10·8	
**(e) Tumour size** (trend χ^2_1 = 0.4; 2p > 0.1: NS)					
**1 – 20 mm (T1)**	380/2774 (13·7%)	503/2808 (17·9%)	−61·3	209·6	0·75 (0·62 – 0·89)
**21 – 50 mm (T2)**	755/3222 (23·4%)	858/3266 (26·3%)	−55·0	372·2	0·86 (0·75 – 0·99)
**> 50 mm (T3/T4)**	130/428 (30·4%)	157/410 (38·3%)	−19·5	57·6	0·71 (0·51 – 1·00)
**(f) Nodal status** (trend χ^2_1 = 0.1; 2p > 0.1: NS)					
**N0**	203/1468 (13·8%)	239/1503 (15·9%)	−16·9	105·8	0·85 (0·66 – 1·09)
**N1–3**	578/3394 (17·0%)	693/3354 (20·7%)	−67·2	298·4	0·80 (0·69 – 0·93)
**N4+**	499/1651 (30·2%)	606/1715 (35·3%)	−49·5	239·6	0·81 (0·69 – 0·96)
**(g) ER/PR status** (χ^2_2 = 0.4; p > 0.1; NS)					
**ER– PR any**	608/2239 (27·2%)	948/2268 (41·8%)	−61·1	298·3	0·81 (0·70 – 0·95)
**ER? PR any**	139/501 (27·7%)	163/528 (30·9%)	−11·5	65·0	0·84 (0·61 – 1·15)
**ER+ PR+**	648/3694 (17·5%)	739/3663 (20·2%)	−54·4	330·6	0·85 (0·74 – 0·98)
**ER+ PR–**	188/795 (23·6%)	232/838 (27·7%)	−21·5	97·1	0·80 (0·62 – 1·04)
**ER+ PR?**	85/490 (17·3%)	100/496 (20·2%)	−6·2	43·1	0·87 (0·59 – 1·28)
**(h) HER2 status** (χ^2_1 = 1.8; 2p > 0.1; NS)					
**HER2 positive**	210/1002 (21·0%)	223/1021 (21·8%)	−3·5	87·9	0·96 (0·73 – 1·26)
**HER2 negative**	810/4359 (18·6%)	976/4386 (22·3%)	−80·2	409·1	0·82 (0·72 – 0·93)
**(i) Tumour grade** (trend χ^2_1 = 0.0; 2p > 0.1: NS)					
**Well–differentiated**	27/284 (9·5%)	26/261 (10·0%)	1·3	12·1	
**Moderately**	339/2245 (15·1%)	401/2231 (18·0%)	−37·0	175·2	0·81 (0·67 – 0·98)
**Poorly**	552/2539 (21·7%)	642/2599 (24·7%)	−40·9	278·0	0·86 (0·74 – 1·01)
**(j) Ki67** (trend χ^2_1 = 0.9; 2p > 0.1: NS)					
**Ki67 <10%**	73/562 (13·0%)	86/516 (16·7%)	−8·6	31·7	0·76 (0·48 – 1·20)
**Ki67 >=10% – <20%**	115/790 (14·6%)	148/820 (18·0%)	−13·9	54·9	0·78 (0·55 – 1·10)
**Ki67 >=20%**	408/2058 (19·8%)	477/2130 (22·4%)	−25·6	194·8	0·88 (0·73 – 1·05)
**(k) Histological type** (χ^2_1 = 1.4; 2p > 0.1; NS)					
**Ductal**	835/3845 (21·7%)	956/3946 (24·2%)	−51·2	398·4	0·88 (0·77 – 1·00)
**Lobular**	106/503 (21·1%)	113/496 (22·8%)	2·7	41·0	
**Total**	**1668/7719 (21·6%)**	**1953/7793 (25·1%)**	**−146·1**	**814·3**	**0·836 (0·780 – 0·895)** 2p < 0·00001

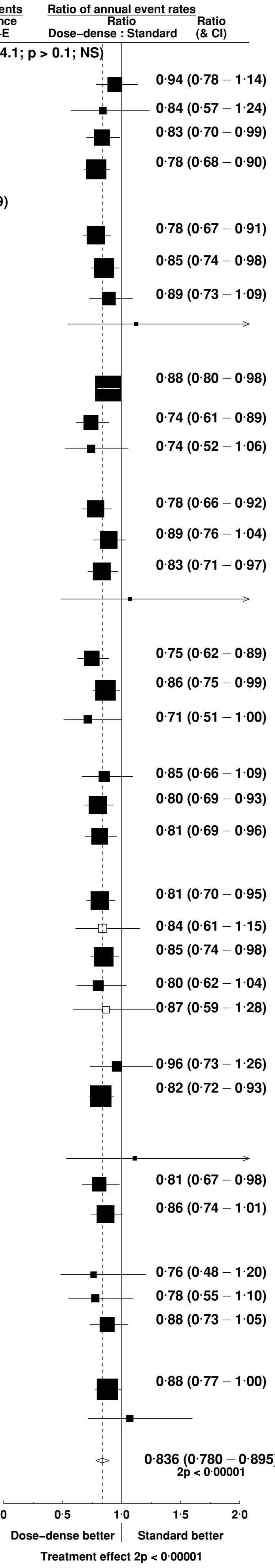

■ 99% or ◇ 95% confidence intervals

* Unknowns omitted

P14: Recurrence for trials with and without taxane, and 4 or fewer or 6 or more dose-dense cycles (all dose-dense trials)

Year code and study name	Comparison	Dose–intensity ratio	Events/Women: Allocated Dose–dense	Events/Women: Allocated Standard	Dose–dense events: Logrank O–E	Dose–dense events: Variance of O–E	Ratio of annual event rates Dose–dense : Standard
**(a) Anthracycline; 4 or fewer dose–dense cycles**							
93W Hamburg Germany §	4E120C600q2† v 4E90C600q3;3CMFq4	E2.0	*(183 patients)*		*(no data)*		
97X Bayreuth Germany	3E120C600‡ [q2† v q3]	E1.5	4/71	9/69	−1·6	2·5	
03D Royal Marsden London §	(4AC/4EC)‡ [q2† v q3]	A1.5 / E1.5	*(128 patients)*		*(no data)*		
05P TACT2 UK	4E100 [q2† v q3];4CMF q4/4Cap2500x14 q3	E1.5	383/2170	412/2221	−9·8	188·4	
**(a) subtotal with data §**			**387/ 2241 (17·3%)**	**421/ 2290 (18·4%)**	**−11·4**	**190·8**	**0·94 (0·82 – 1·09) reduction 2p > 0·1; NS**
**Difference between treatment effects in 2 trials: $\chi^2_1 = 0·9$; 2p > 0·1; NS**							
**(b) Taxane and anthracycline; 4 or fewer dose–dense cycles**							
99Y GEPARDUO Germany	[4A50D75q2† v (4AC;4D100)q3]‡	A1.3 D1.1	91/454	104/456	−7·8	44·8	**0·84 (0·63 – 1·13) reduction 2p > 0·1; NS**
**(c) Anthracycline; 6 or more dose–dense cycles**							
92E GONO MIG1 Italy	6F600E60C600 [q2† v q3]	E1.5	149/604	173/610	−6·7	70·7	
92J Pisa/Genoa Italy	[3FEC‡;3(F600E60C600; CMF)] [q2† v q3]	E1.5	28/73	35/77	−2·8	12·4	
93# EORTC 10921/MA.10	(6E120C830q2† v 6F500x2E60x2C75x14q4)‡	E2.0	139/224	132/224	1·8	53·2	
00F MA.21 Canada	(6E120C830q2† v 4AC q3); 4P175q3	E/A2.0	168/701	221/702	−33·9	90·3	
**(c) subtotal**			**484/ 1602 (30·2%)**	**561/ 1613 (34·8%)**	**−41·7**	**226·6**	**0·83 (0·73 – 0·95) reduction 2p = 0·006**
**Heterogeneity between 4 trials: $\chi^2_3 = 6·4$; p = 0·09**							
**(d) Taxane and anthracycline; 6 or more dose–dense cycles**							
97D CALGB 9741 USA	[(4A60;4P175;4C600) or (4AC;4P175)][q2† v q3]	A1.5 P1.5	256/1001	315/1004	−36·5	129·8	
00Y GASG Germany §	(4E120†;4P175)q2 v (4EC;4P175)q3	E2.0 P1.5	*(884 patients)*		*(no data)*		
02C PREPARE/GermanBG 49	[(3E150;3P225)q2†;3CMF q4 v (4EC;4P175)q3†]‡	E2.5 P1.9	118/363	128/370	−1·2	55·5	
03Q GIM 2 Italy	[4(E90C600 ± F600);4P175] [q2† v q3]	E1.5 P1.5	201/1002	257/1001	−33·1	97·9	
04) CAMS China	6E120P175 [q2† v q3]	E1.5 P1.5	9/50	10/51	−1·0	3·6	
07B SBG PANTHER Sweden	(4E100C825;4D80)q2† v (3F500E100C500;3D100)q3†	E1.5 D1.2	122/1006	157/1008	−13·5	65·3	
**(d) subtotal with data §**			**706/ 3422 (20·6%)**	**867/ 3434 (25·2%)**	**−85·3**	**352·0**	**0·78 (0·71 – 0·87) reduction 2p < 0·00001**
**Heterogeneity between 5 trials: $\chi^2_4 = 3·9$; p > 0·1; NS**							
**Total (a + b + c + d)**			**1668/ 7719 (21·6%)**	**1953/ 7793 (25·1%)**	**−146·1**	**814·3**	**0·836 (0·780 – 0·895) reduction 2p < 0·00001**

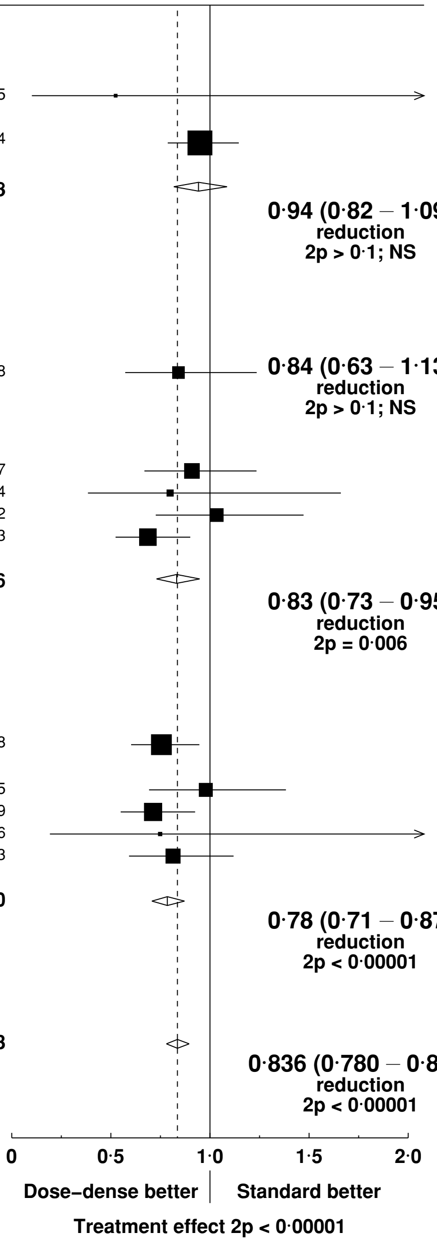

99% or 95% confidence intervals

Heterogeneity between 4 subtotals: $\chi^2_3 = 4·1$; p > 0·1; NS

Heterogeneity within subtotals: $\chi^2_8 = 11·2$; p > 0·1; NS

Heterogeneity between 12 trials: $\chi^2_{11} = 15·3$; p > 0·1; NS

§ 3 trials with no data do not contribute to subtotals or to the overall total. ‡ Pre-operative chemotherapy: patients in these trials were analysed as unknown nodal status. † Primary prophylaxis with colony-stimulating growth factors. **Abbreviations**: Taxanes: **D** = docetaxel; **P** = paclitaxel. Anthracyclines: **A** = doxorubicin; **E** = epirubicin.
Other: **C** = cyclophosphamide; **Cap** = capecitabine; **F** = fluorouracil; **M** = methotrexate; **Bev** = bevacizumab (mg/kg). **AC=A60C600; EC=E90C600; ADC= A50D75C500; CMF** = classical CMFd1d8. Chemotherapy doses are in mg/m2.**q2** = 2-weekly, **q3** = 3-weekly, **q4** = 4-weekly. ×14 means d1-14 po; ×2 means d1, d8. Semicolon [;] indicates treatment sequence.

P15: 10-year risk of recurrence and breast cancer mortality in all dose-dense trials with 6 or more dose-dense cycles (A) anthracycline only, and (B) anthracycline and taxane

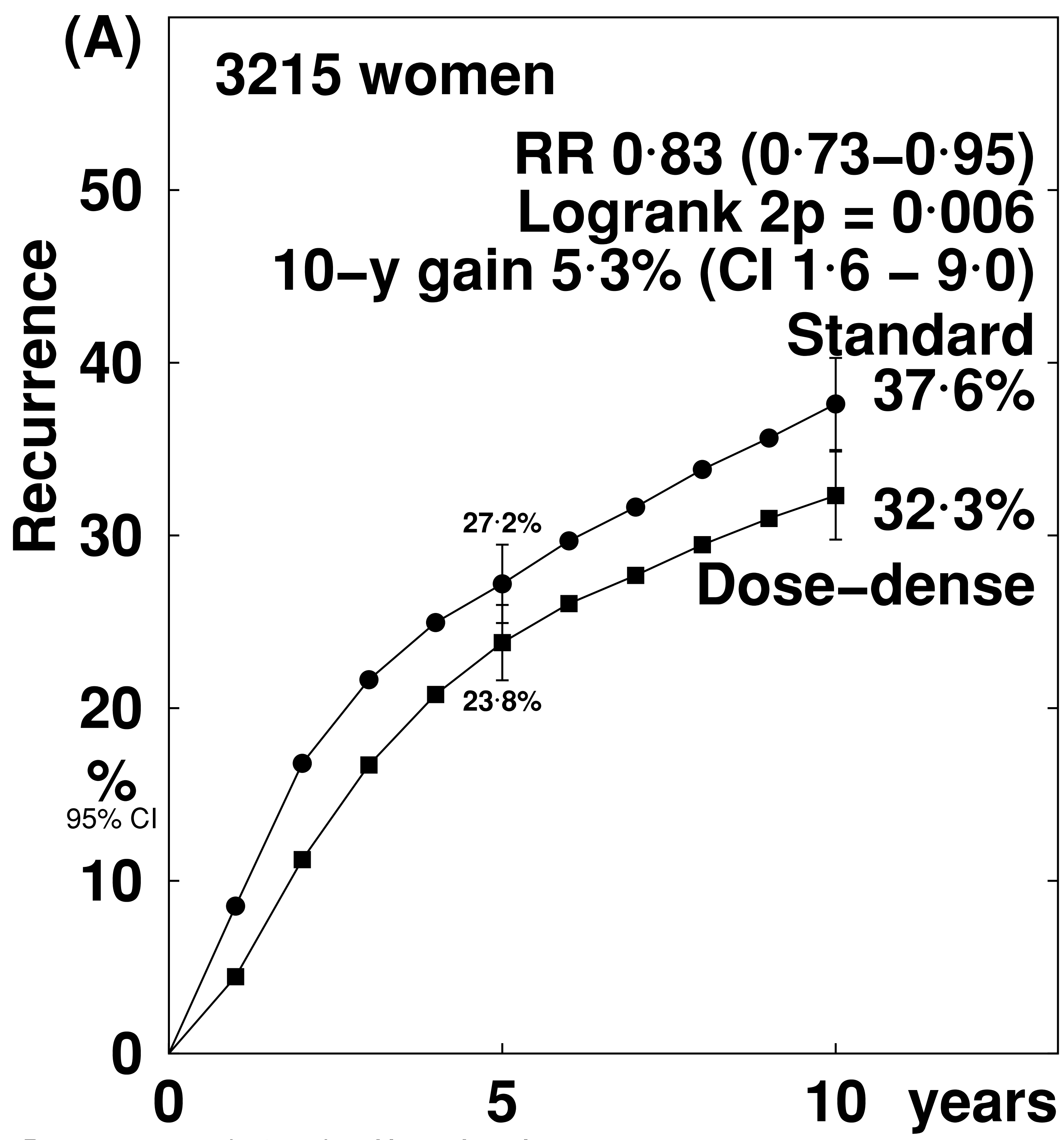

Recurrence rates (% / year) and logrank analyses

Allocation	Years 0 – 4	Years 5 – 9	Year 10+
Dose–dense	5·53 (375 / 6781)	2·44 (99 / 4063)	0·90 (10 / 1111)
Standard	6·56 (428 / 6525)	3·14 (124 / 3954)	0·76 (9 / 1185)
Rate ratio, from (O–E) / V	0·83 CI 0·70 – 0·97<br>–31·1 / 171·5	0·79 CI 0·54 – 1·03<br>–12·3 / 51·0	1·57 CI 0·35 – 2·80<br>1·8 / 4·1

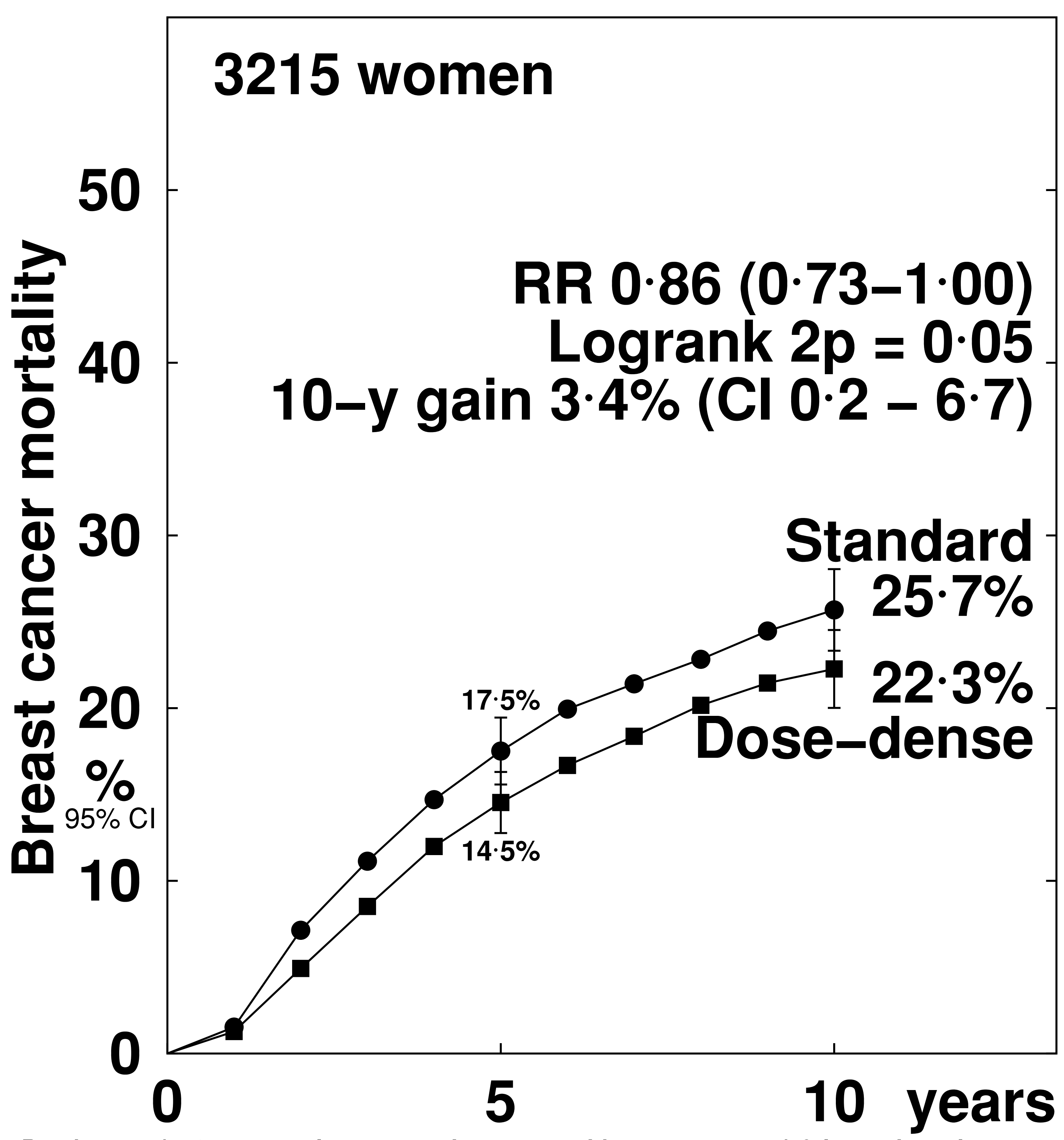

Death rates (% / year: total rate – rate in women without recurrence) & logrank analyses

Allocation	Years 0 – 4	Years 5 – 9	Year 10+
Dose–dense	3·08 CI 2·68 – 3·49	2·07 CI 1·66 – 2·49	0·88 CI 0·36 – 1·40
Standard	3·87 CI 3·41 – 4·32	2·12 CI 1·70 – 2·54	0·82 CI 0·34 – 1·31
Rate ratio, from (O–E) / V	0·81 CI 0·64 – 0·98<br>–23·2 / 111·1	0·94 CI 0·65 – 1·22<br>–2·9 / 43·8	1·26 CI 0·29 – 2·23<br>1·2 / 5·2

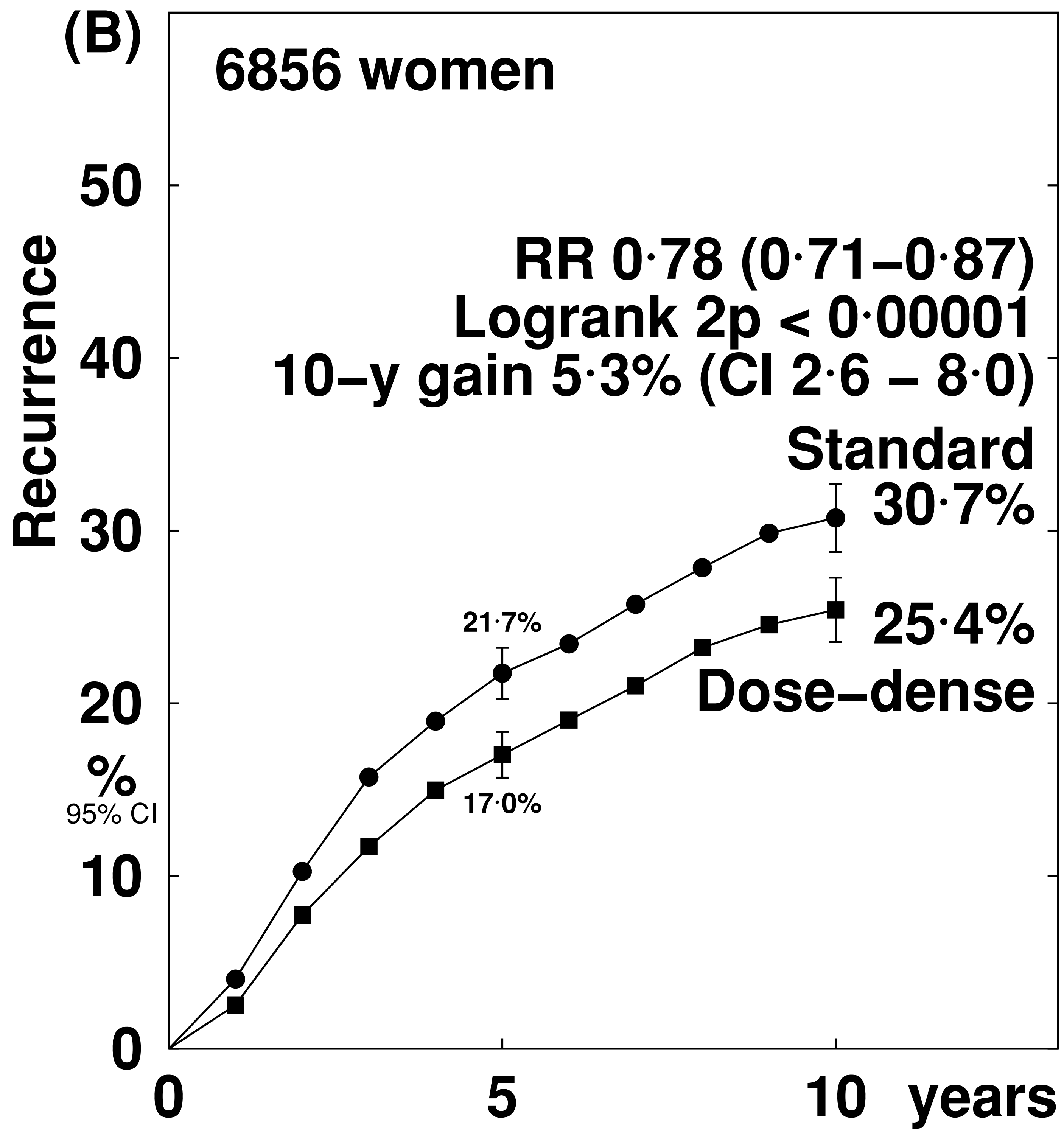

Recurrence rates (% / year) and logrank analyses

Allocation	Years 0 – 4	Years 5 – 9	Year 10+
Dose–dense	3·76 (546 / 14520)	2·32 (141 / 6065)	1·27 (19 / 1493)
Standard	5·01 (705 / 14060)	2·48 (142 / 5720)	1·39 (20 / 1442)
Rate ratio, from (O–E) / V	0·75 CI 0·65 – 0·86<br>–78·9 / 278·9	0·91 CI 0·68 – 1·15<br>–6·0 / 64·4	0·96 CI 0·31 – 1·61<br>–0·4 / 8·7

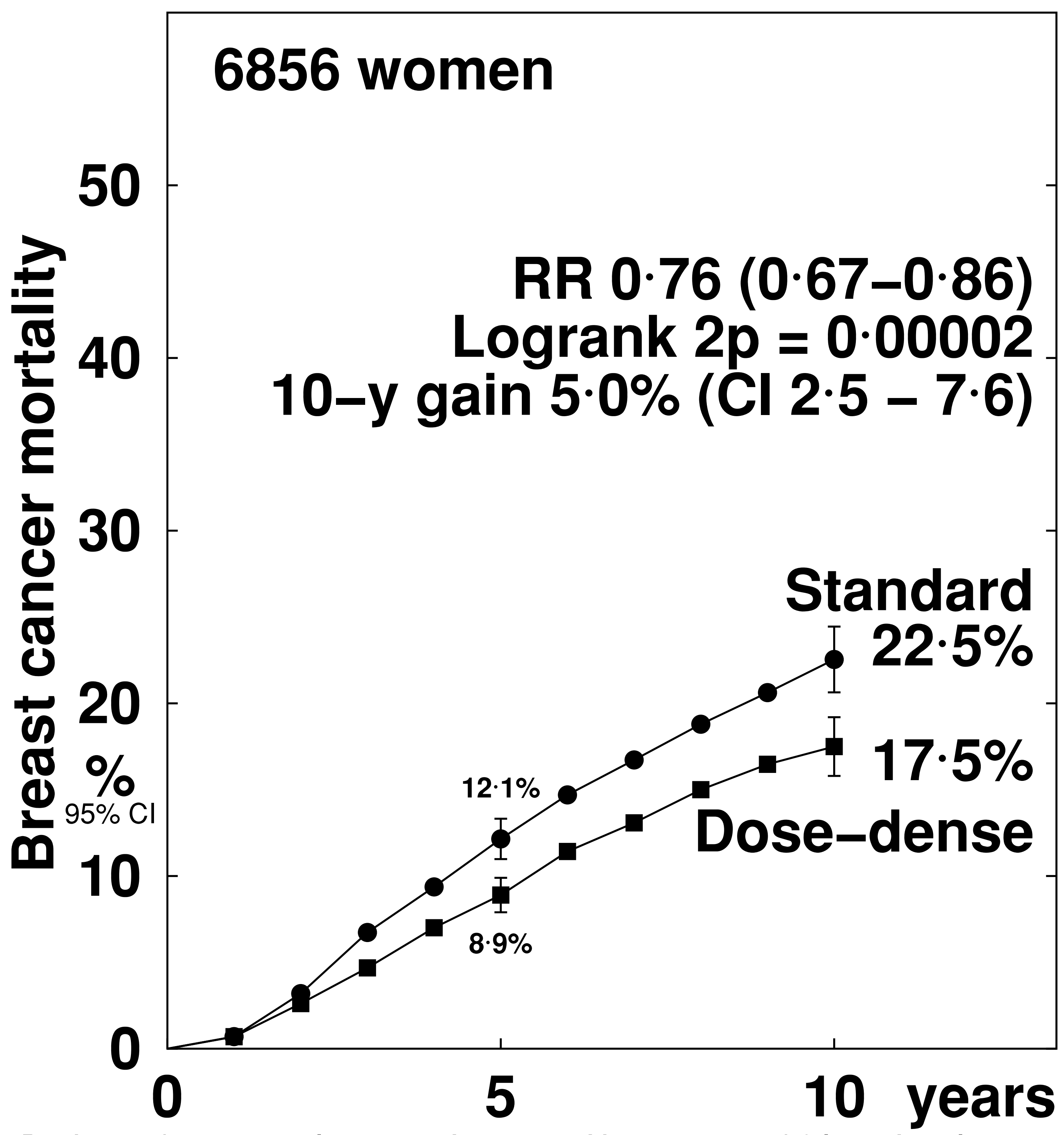

Death rates (% / year: total rate – rate in women without recurrence) & logrank analyses

Allocation	Years 0 – 4	Years 5 – 9	Year 10+
Dose–dense	1·80 CI 1·59 – 2·02	2·18 CI 1·83 – 2·53	1·79 CI 1·14 – 2·44
Standard	2·57 CI 2·31 – 2·82	2·56 CI 2·17 – 2·95	2·12 CI 1·41 – 2·83
Rate ratio, from (O–E) / V	0·72 CI 0·59 – 0·86<br>–49·8 / 152·2	0·83 CI 0·62 – 1·04<br>–13·1 / 71·8	0·85 CI 0·38 – 1·32<br>–2·4 / 14·6

P16: Subgroup analyses for breast cancer mortality (all trials)* calculated by logrank subtraction

Category	Events/Women: Allocated Dose-intense	Events/Women: Allocated Standard	Dose-intense events: Logrank O–E	Dose-intense events: Variance of O–E	Ratio of annual event rates: Ratio (& CI)
**(a) Dose-intense trial groupings (A to D)** (χ^2_6 = 5.4; p > 0.1; NS)					
**Trial Group A1 (P7)**	685/4971 (13·8%)	797/5033 (15·8%)	−51·4	339·9	**0·86 (0·75 – 0·99)**
**Trial group A2**	239/1684 (14·2%)	255/1688 (15·1%)	−5·4	110·4	**0·95 (0·75 – 1·22)**
**Trial Group A3**	176/1064 (16·5%)	231/1072 (21·5%)	−26·4	95·7	**0·76 (0·58 – 0·99)**
**Trial Group B1**	651/4820 (13·5%)	719/4824 (14·9%)	−40·8	324·1	**0·88 (0·76 – 1·02)**
**Trial Group B2**	61/669 (9·1%)	71/715 (9·9%)	−0·3	31·2	
**Trial Group C**	558/3323 (16·8%)	641/3284 (19·5%)	−55·8	275·3	**0·82 (0·70 – 0·95)**
**Trial Group D**	520/2092 (24·9%)	572/2134 (26·8%)	−20·0	254·9	**0·92 (0·79 – 1·09)**
**(b) Follow up period, years** (trend χ^2_1 = 0.2; 2p > 0.1; NS)					
**0–1**	545/18623 (2·9%)	630/18750 (3·4%)	−33·2	267·3	**0·88 (0·75 – 1·03)**
**2–4**	1298/17498 (7·4%)	1478/17522 (8·4%)	−89·5	643·8	**0·87 (0·79 – 0·96)**
**5–9**	872/14175 (6·2%)	973/14002 (6·9%)	−60·2	431·9	**0·87 (0·77 – 0·98)**
**10+**	175/3923 (4·5%)	205/3823 (5·4%)	−17·1	88·2	**0·82 (0·63 – 1·08)**
**(c) Age** (trend χ^2_1 = 0.9; 2p > 0.1: NS)					
**Age < 45**	968/5319 (18·2%)	1083/5283 (20·5%)	−67·2	475·6	**0·87 (0·77 – 0·98)**
**45 – 54**	956/6884 (13·9%)	1018/7007 (14·5%)	−29·7	463·7	**0·94 (0·83 – 1·06)**
**55 – 69**	912/6109 (14·9%)	1124/6145 (18·3%)	−103·5	470·9	**0·80 (0·71 – 0·90)**
**70+**	54/310 (17·4%)	61/312 (19·6%)	0·3	21·3	
**(d) Tumour size** (trend χ^2_1 = 0.4; 2p > 0.1: NS)					
**1 – 20 mm (T1)**	670/6932 (9·7%)	841/7137 (11·8%)	−73·4	360·1	**0·82 (0·71 – 0·93)**
**21 – 50 mm (T2)**	1569/8659 (18·1%)	1731/8595 (20·1%)	−105·2	773·5	**0·87 (0·80 – 0·96)**
**> 50 mm (T3/T4)**	310/1197 (25·9%)	328/1164 (28·2%)	−23·9	131·9	**0·83 (0·67 – 1·04)**
**(e) Nodal status** (trend χ^2_1 = 2.5; 2p > 0.1: NS)					
**N0**	253/2552 (9·9%)	253/2623 (9·6%)	5·5	121·9	
**N1–3**	1012/8738 (11·6%)	1178/8746 (13·5%)	−83·0	521·4	**0·85 (0·76 – 0·95)**
**N4+**	1286/5617 (22·9%)	1484/5661 (26·2%)	−104·5	631·6	**0·85 (0·76 – 0·94)**
**(f) ER/PR status** (χ^2_2 = 0.2; p > 0.1; NS)					
**ER− PR any**	1189/5406 (22·0%)	1369/5520 (24·8%)	−84·0	591·4	**0·87 (0·78 – 0·96)**
**ER? PR any**	116/678 (17·1%)	154/691 (22·3%)	−19·7	60·8	**0·72 (0·52 – 1·01)**
**ER+ PR+**	1168/9941 (11·7%)	1286/9895 (13·0%)	−75·2	590·0	**0·88 (0·79 – 0·98)**
**ER+ PR−**	354/2033 (17·4%)	406/2074 (19·6%)	−29·3	178·1	**0·85 (0·70 – 1·03)**
**ER+ PR?**	63/565 (11·2%)	71/570 (12·5%)	−3·2	32·0	
**(g) HER2 status** (χ^2_1 = 0.0; 2p > 0.1; NS)					
**HER2 positive**	232/1503 (15·4%)	261/1491 (17·5%)	−19·2	103·5	**0·83 (0·64 – 1·07)**
**HER2 negative**	1050/7811 (13·4%)	1248/7868 (15·9%)	−98·6	530·9	**0·83 (0·74 – 0·93)**
**(h) Tumour grade** (trend χ^2_1 = 0.8; 2p > 0.1: NS)					
**Well-differentiated**	34/896 (3·8%)	38/848 (4·5%)	−3·3	17·0	
**Moderately**	536/5190 (10·3%)	626/5188 (12·1%)	−55·4	278·3	**0·82 (0·70 – 0·96)**
**Poorly**	961/5317 (18·1%)	1083/5401 (20·1%)	−62·3	482·4	**0·88 (0·78 – 0·99)**
**(i) Ki67** (trend χ^2_1 = 0.1; 2p > 0.1: NS)					
**Ki67 <10%**	37/564 (6·6%)	44/518 (8·5%)	−4·0	15·4	
**Ki67 >=10% – <20%**	69/803 (8·6%)	88/826 (10·7%)	−4·9	31·8	
**Ki67 >=20%**	256/2084 (12·3%)	315/2168 (14·5%)	−19·1	126·8	**0·86 (0·68 – 1·08)**
**(j) Histological type** (χ^2_1 = 0.5; 2p > 0.1; NS)					
**Ductal**	1264/8733 (14·5%)	1418/8831 (16·1%)	−76·0	618·5	**0·88 (0·80 – 0·98)**
**Lobular**	185/1250 (14·8%)	223/1271 (17·5%)	−17·2	82·9	**0·81 (0·61 – 1·08)**
**Total**	**2890/18623 (15·5%)**	**3286/18750 (17·5%)**	**−200·1**	**1431·5**	**0·870 (0·826 – 0·916)** 2p < 0·00001

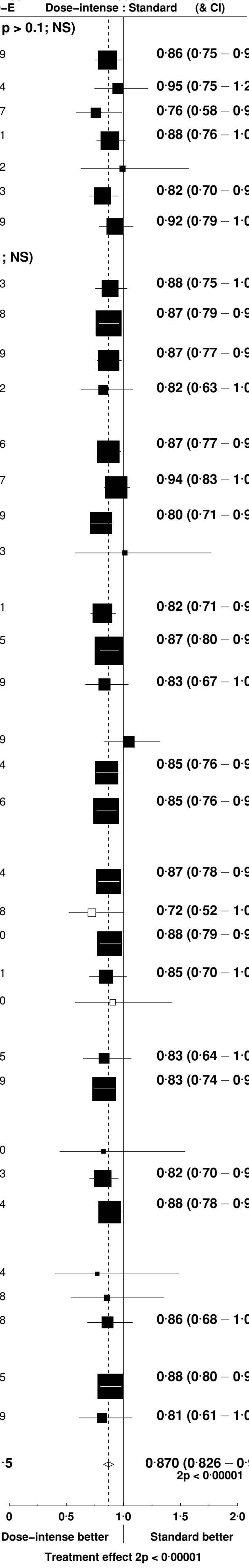

99% or 95% confidence intervals

* Unknowns omitted

P17: 10-year risk of (A) distant recurrence at any time, (B) distant recurrence as first event, (C) local recurrence as first event and (D) contralateral breast cancer as first event (all trials)

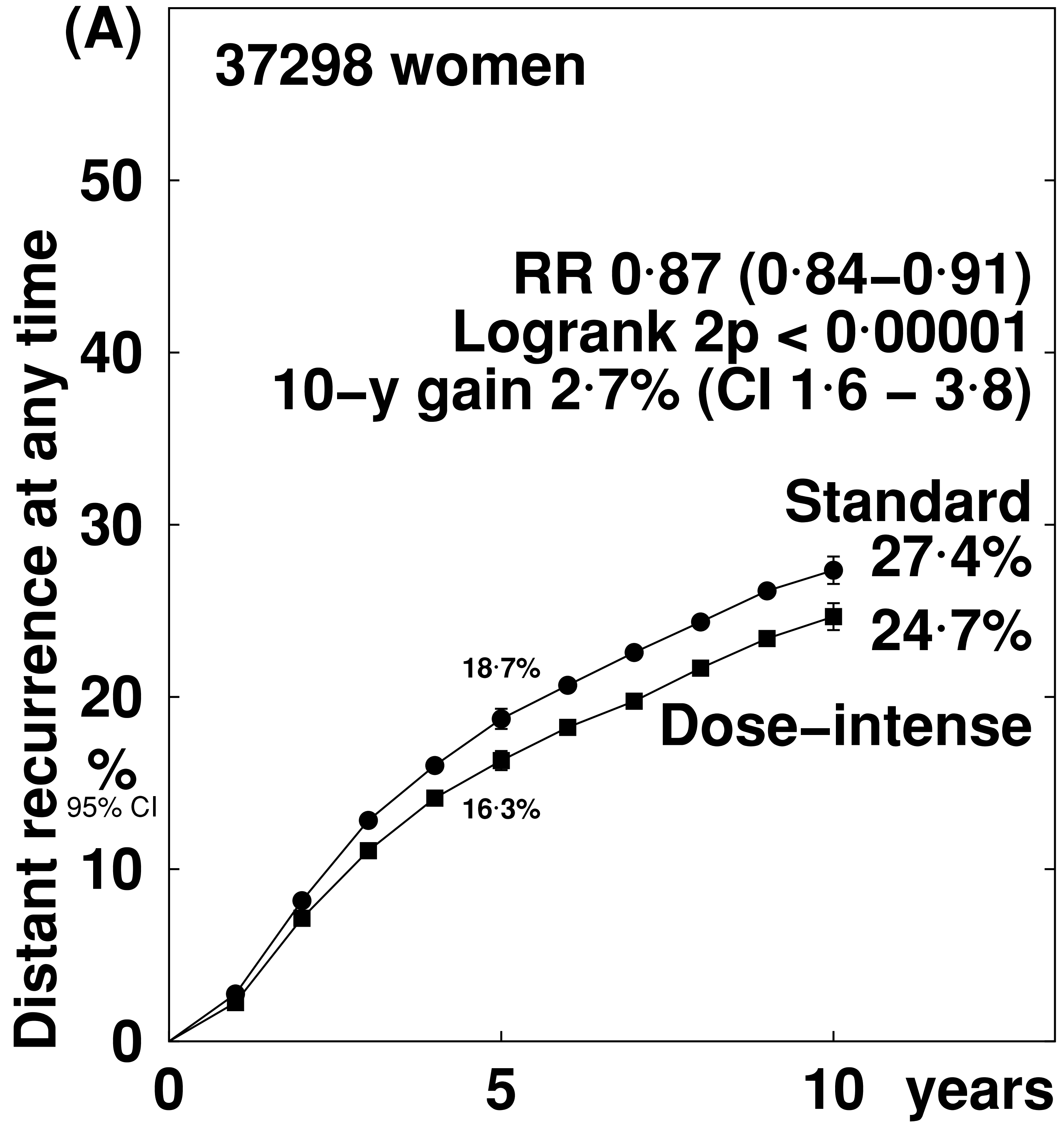

Distant recurrence rates (% / year) and logrank analyses

Allocation	Years 0 – 4	Years 5 – 9	Year 10+
Dose–intense	3·58 (2913 / 81259)	2·16 (818 / 37830)	1·39 (154 / 11107)
Standard	4·16 (3340 / 80337)	2·31 (845 / 36543)	1·40 (152 / 10894)
Rate ratio, from (O–E) / V	0·86 CI 0·81 – 0·90<br>–221·7 / 1429·4	0·92 CI 0·83 – 1·02<br>–31·4 / 393·3	0·98 CI 0·75 – 1·21<br>–1·6 / 72·5

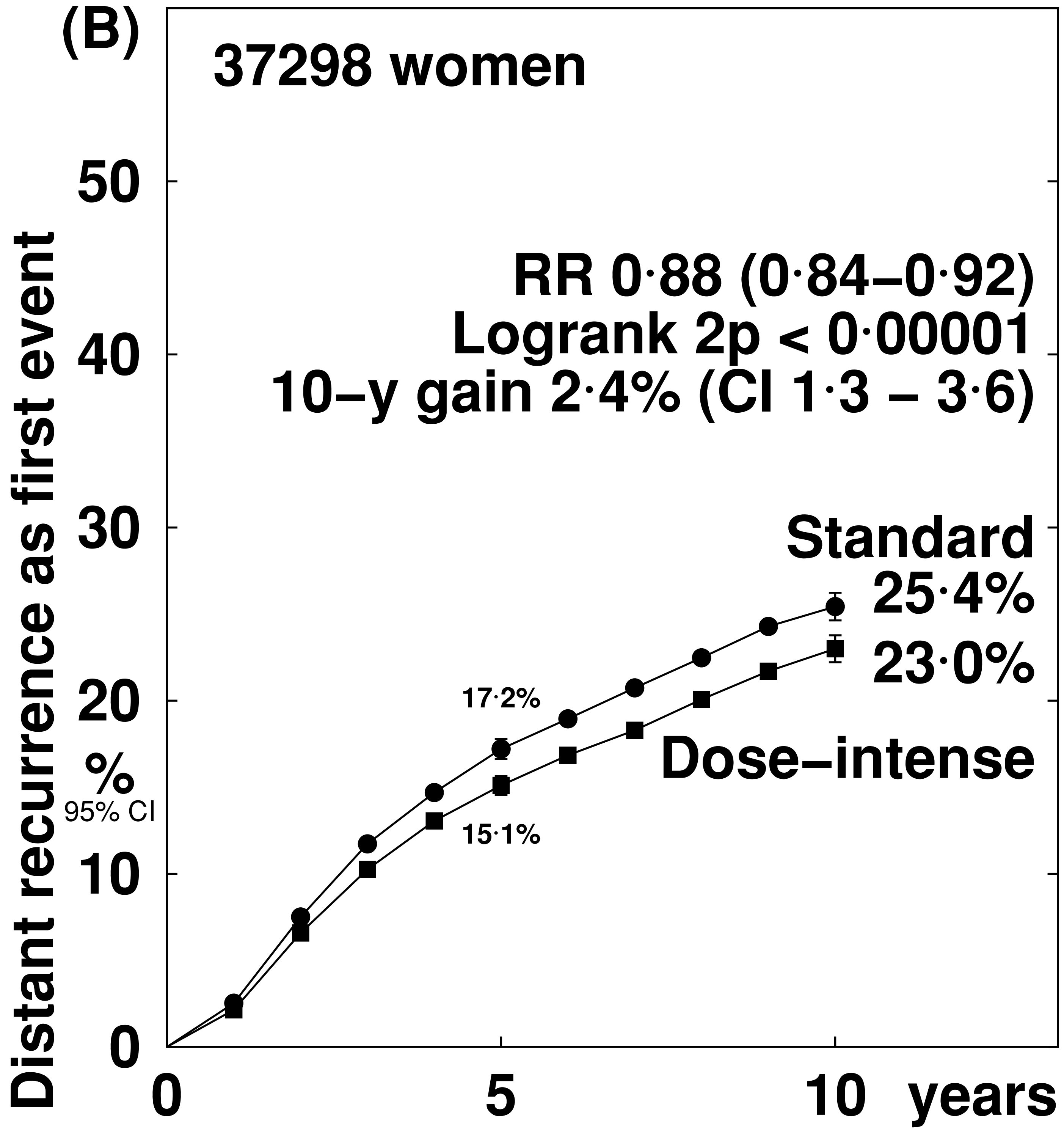

Distant recurrence as first event rates (% / year) and logrank analyses

Allocation	Years 0 – 4	Years 5 – 9	Year 10+
Dose–intense	3·29 (2643 / 80232)	1·99 (730 / 36772)	1·35 (144 / 10657)
Standard	3·79 (2992 / 78935)	2·13 (750 / 35232)	1·34 (139 / 10373)
Rate ratio, from (O–E) / V	0·87 CI 0·81 – 0·92<br>–187·2 / 1296·7	0·92 CI 0·82 – 1·02<br>–28·8 / 351·0	1·00 CI 0·76 – 1·24<br>0·2 / 67·5

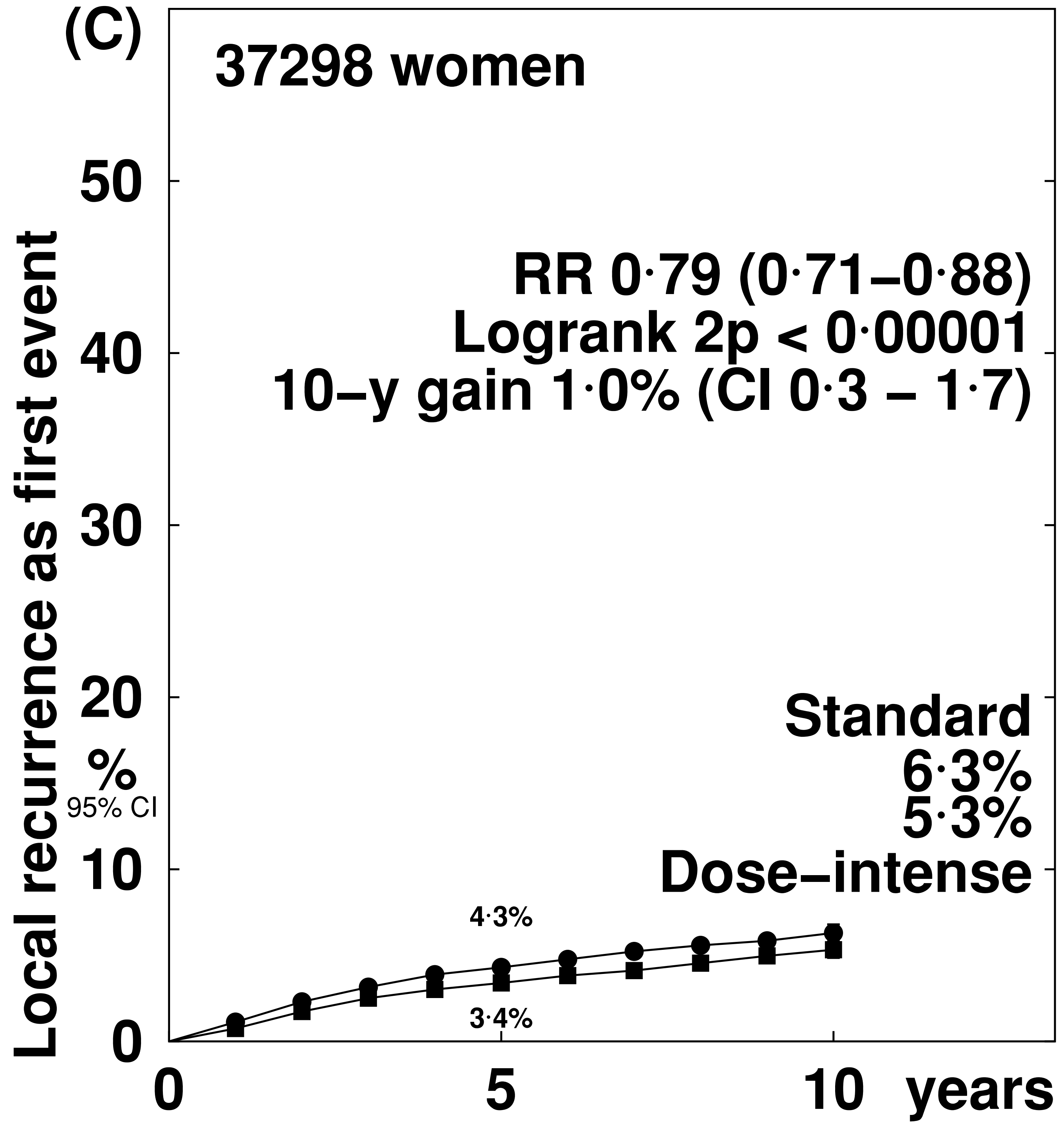

Local recurrence rates (% / year) and logrank analyses

Allocation	Years 0 – 4	Years 5 – 9	Year 10+
Dose–intense	0·71 (520 / 73269)	0·41 (127 / 31107)	0·23 (12 / 5137)
Standard	0·91 (652 / 71831)	0·44 (129 / 29619)	0·54 (27 / 4958)
Rate ratio, from (O–E) / V	0·78 CI 0·68 – 0·89<br>–68·8 / 280·8	0·91 CI 0·67 – 1·15<br>–5·7 / 61·2	0·39 CI –0·04 – 0·82<br>–8·2 / 8·6

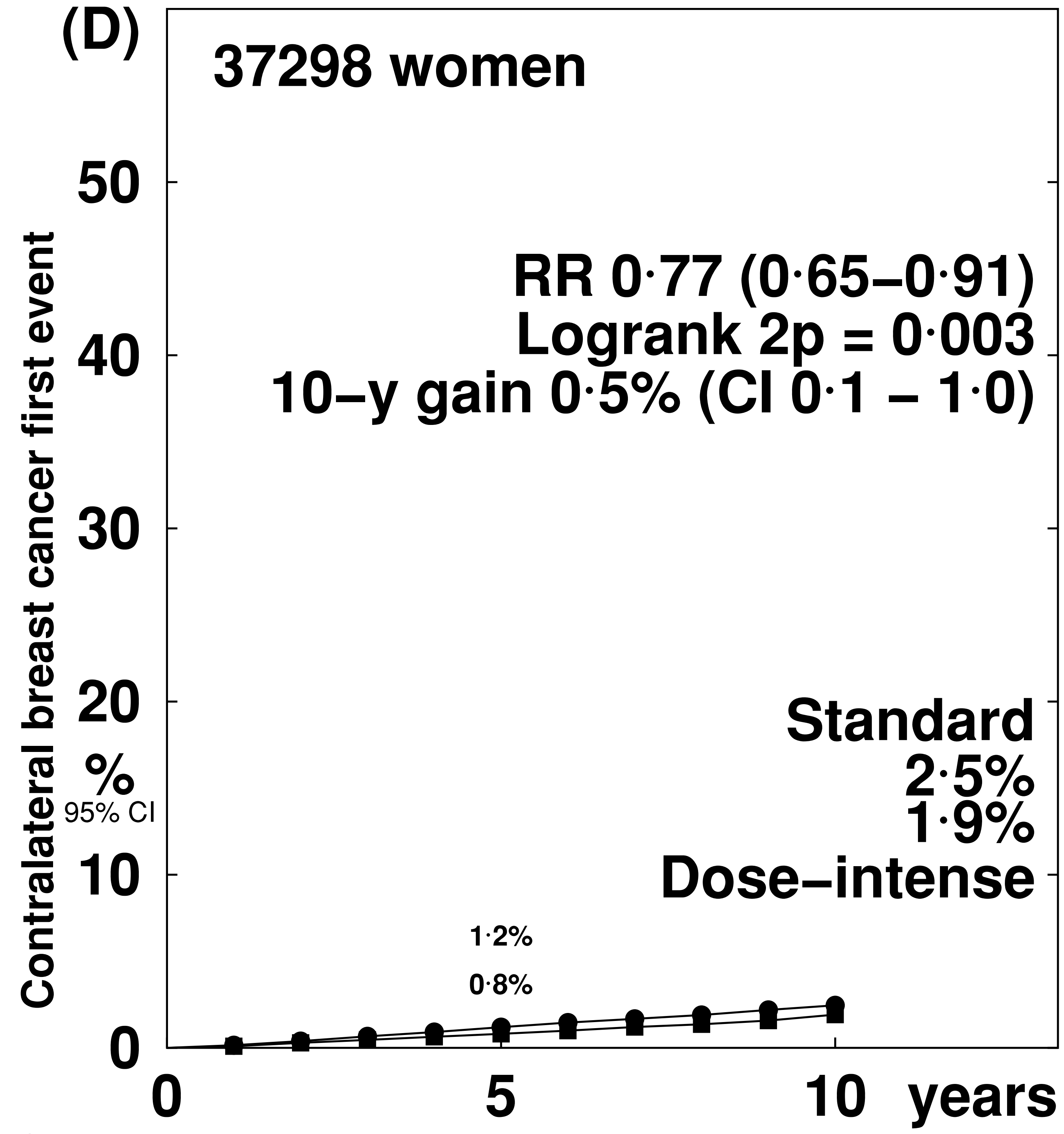

Contralateral breast cancer as first event rates (% / year) and logrank analyses

Allocation	Years 0 – 4	Years 5 – 9	Year 10+
Dose–intense	0·16 (129 / 80227)	0·21 (78 / 36767)	0·29 (31 / 10657)
Standard	0·24 (187 / 78930)	0·25 (88 / 35231)	0·23 (24 / 10373)
Rate ratio, from (O–E) / V	0·68 CI 0·50 – 0·87<br>–29·6 / 77·7	0·83 CI 0·54 – 1·12<br>–7·3 / 38·6	1·25 CI 0·64 – 1·85<br>2·9 / 13·1

P 18: 5-year recurrence split by tumour size in all trials of dose-intense vs standard schedule chemotherapy

1–20 mm (T1)

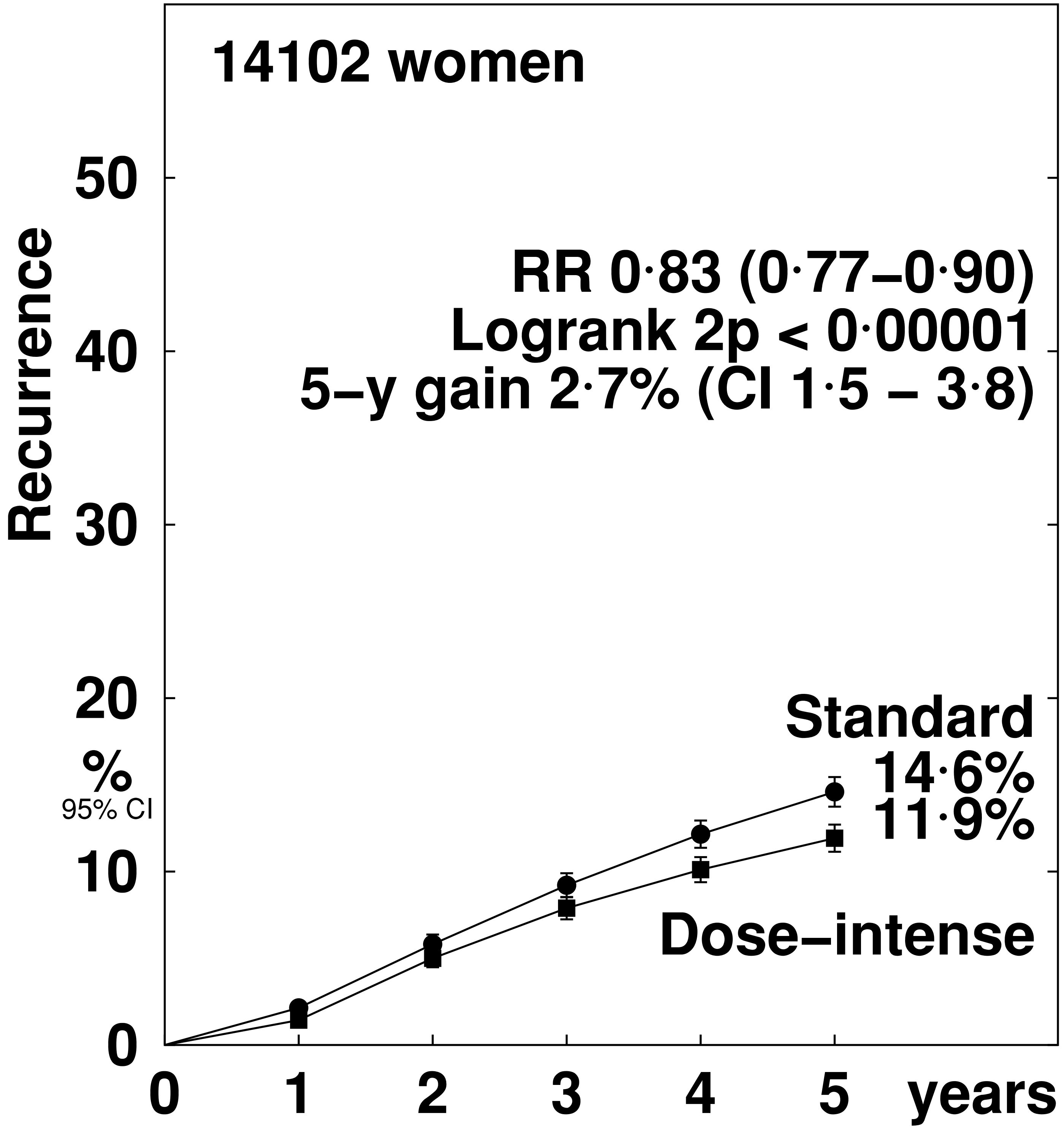

Recurrence rates (% / year) and logrank analyses

Allocation	Years 0 – 1	Years 2 – 4	Year 5+
Dose–intense	2·51 (338 / 13487)	2·53 (457 / 18049)	1·89 (369 / 19477)
Standard	3·01 (413 / 13719)	3·29 (594 / 18046)	2·12 (405 / 19103)
Rate ratio, from (O–E) / V	0·85 CI 0·72 – 0·99<br>–28·3 / 176·8	0·77 CI 0·66 – 0·88<br>–64·4 / 250·3	0·89 CI 0·75 – 1·02<br>–21·9 / 185·6

21–50 mm (T2)

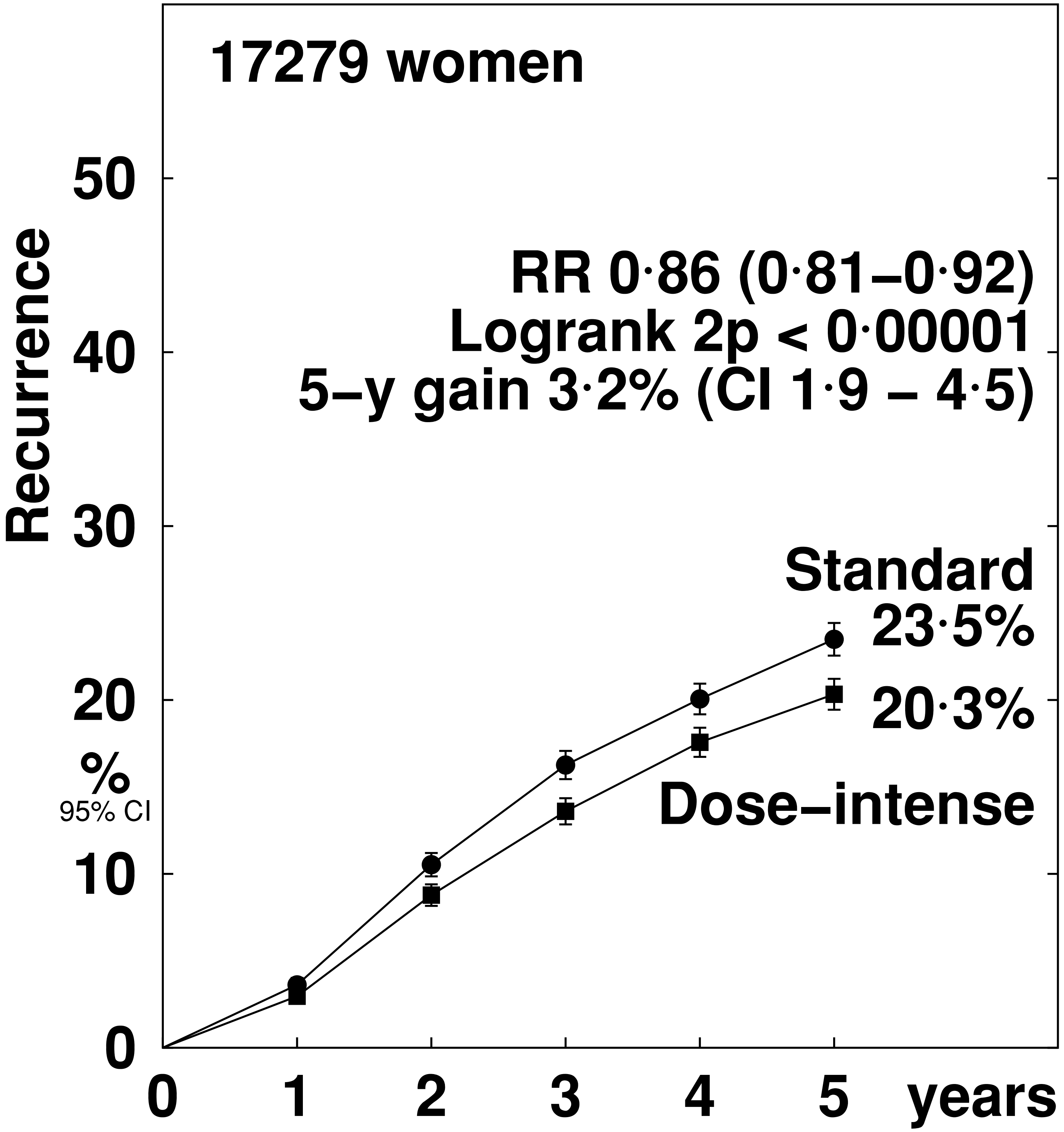

Recurrence rates (% / year) and logrank analyses

Allocation	Years 0 – 1	Years 2 – 4	Year 5+
Dose–intense	4·60 (760 / 16526)	4·60 (958 / 20827)	2·61 (606 / 23236)
Standard	5·45 (884 / 16211)	5·25 (1051 / 20012)	2·76 (610 / 22102)
Rate ratio, from (O–E) / V	0·82 CI 0·73 – 0·91<br>–72·8 / 370·5	0·86 CI 0·78 – 0·95<br>–68·8 / 466·7	0·92 CI 0·81 – 1·03<br>–23·5 / 289·4

P 19: 5-year risk of recurrence split by nodal status in all trials of dose-intense vs standard schedule chemotherapy

N0

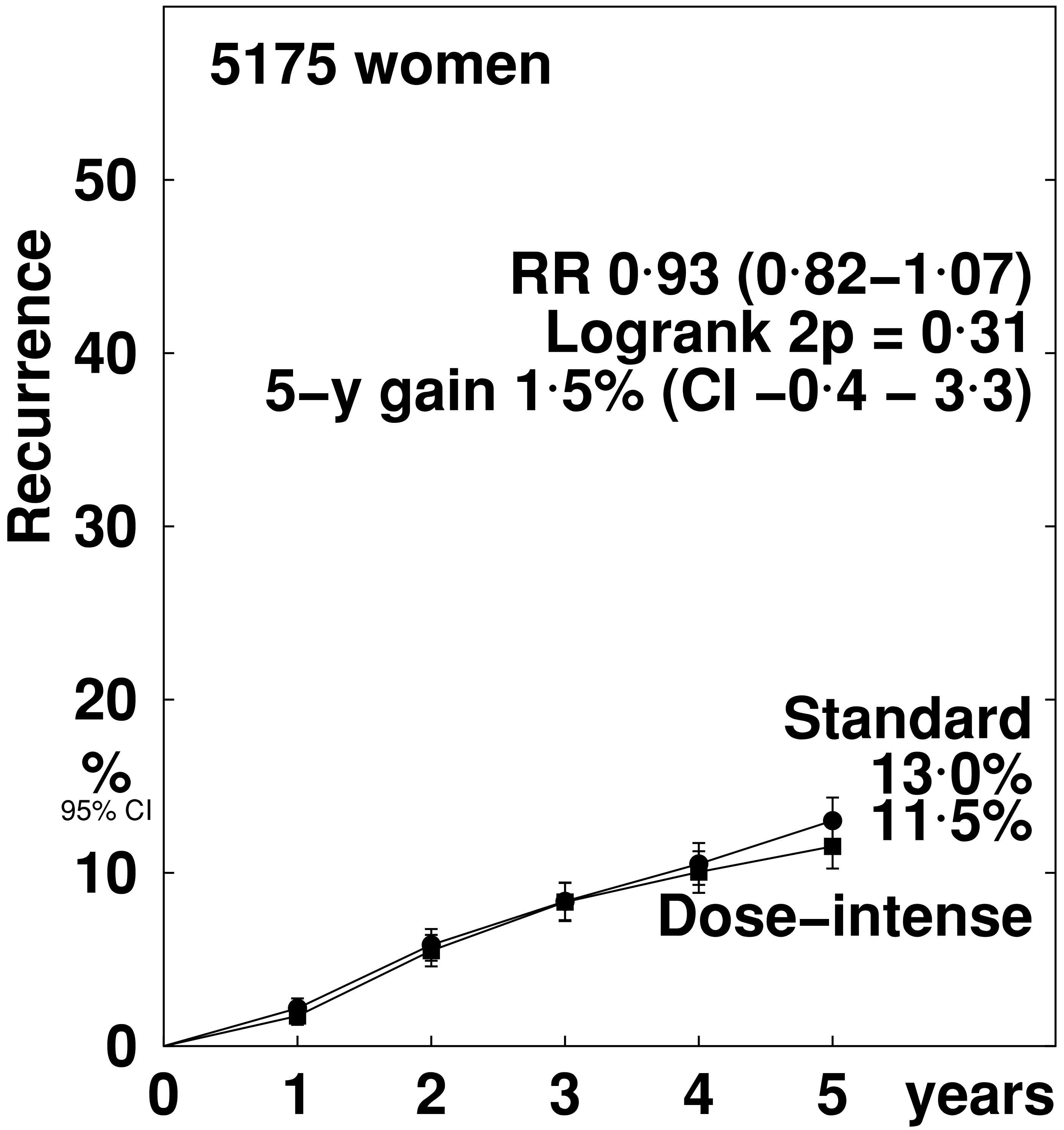

Recurrence rates (% / year) and logrank analyses

Allocation	Years 0 – 1	Years 2 – 4	Year 5+
Dose–intense	2·83 (140 / 4942)	2·20 (145 / 6581)	1·57 (154 / 9827)
Standard	2·97 (150 / 5049)	2·65 (177 / 6688)	1·55 (154 / 9944)
Rate ratio, from	0·94 CI 0·71 – 1·17	0·84 CI 0·64 – 1·04	1·04 CI 0·81 – 1·27
(O–E) / V	–4·4 / 68·6	–13·6 / 77·6	2·9 / 74·0

N1–3

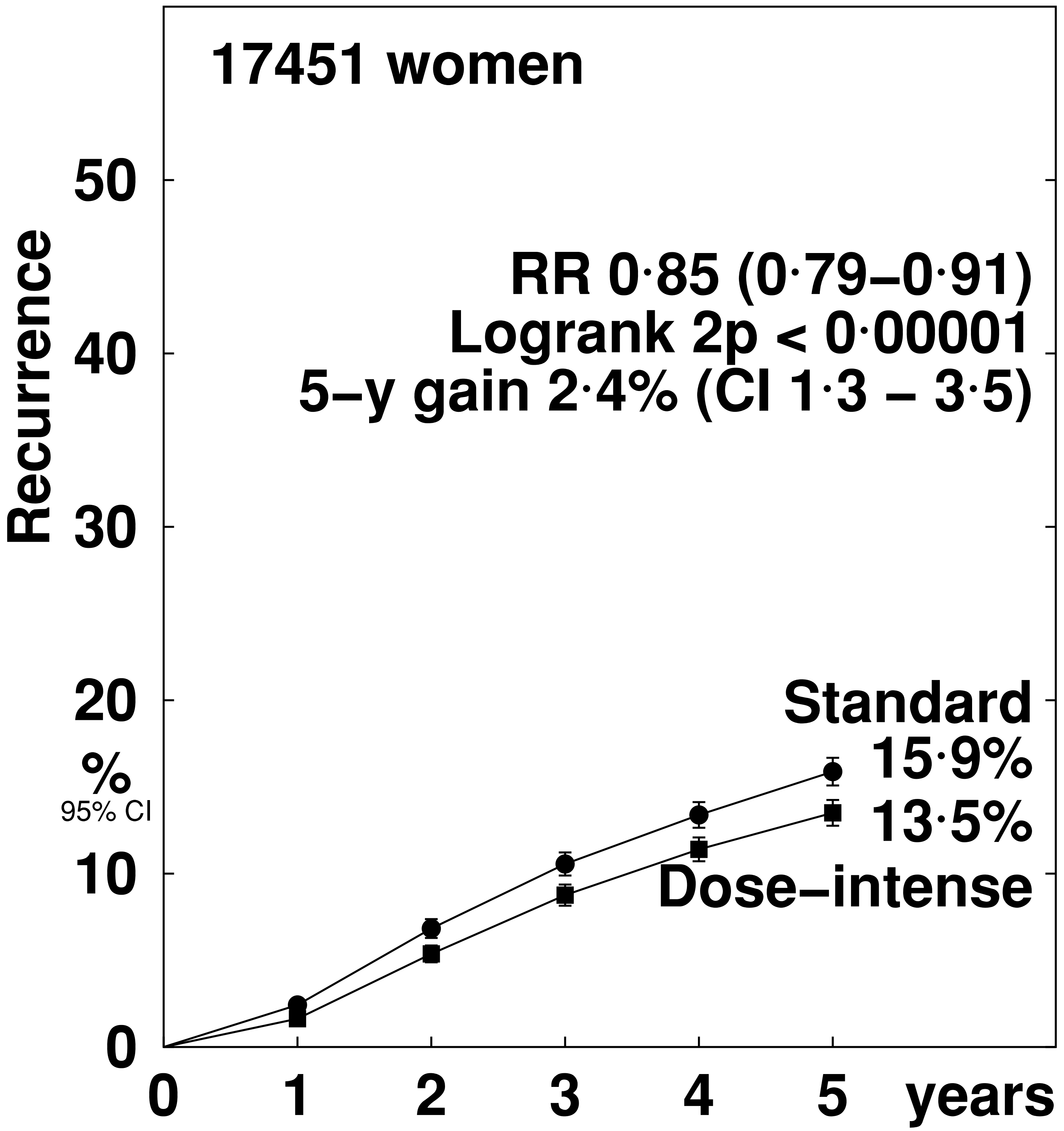

Recurrence rates (% / year) and logrank analyses

Allocation	Years 0 – 1	Years 2 – 4	Year 5+
Dose–intense	2·75 (463 / 16858)	3·02 (673 / 22312)	2·01 (499 / 24825)
Standard	3·51 (585 / 16672)	3·44 (749 / 21779)	2·29 (546 / 23860)
Rate ratio, from	0·78 CI 0·66 – 0·89	0·88 CI 0·78 – 0·98	0·87 CI 0·76 – 0·99
(O–E) / V	–62·0 / 243·4	–43·4 / 337·8	–34·1 / 251·3

N4+

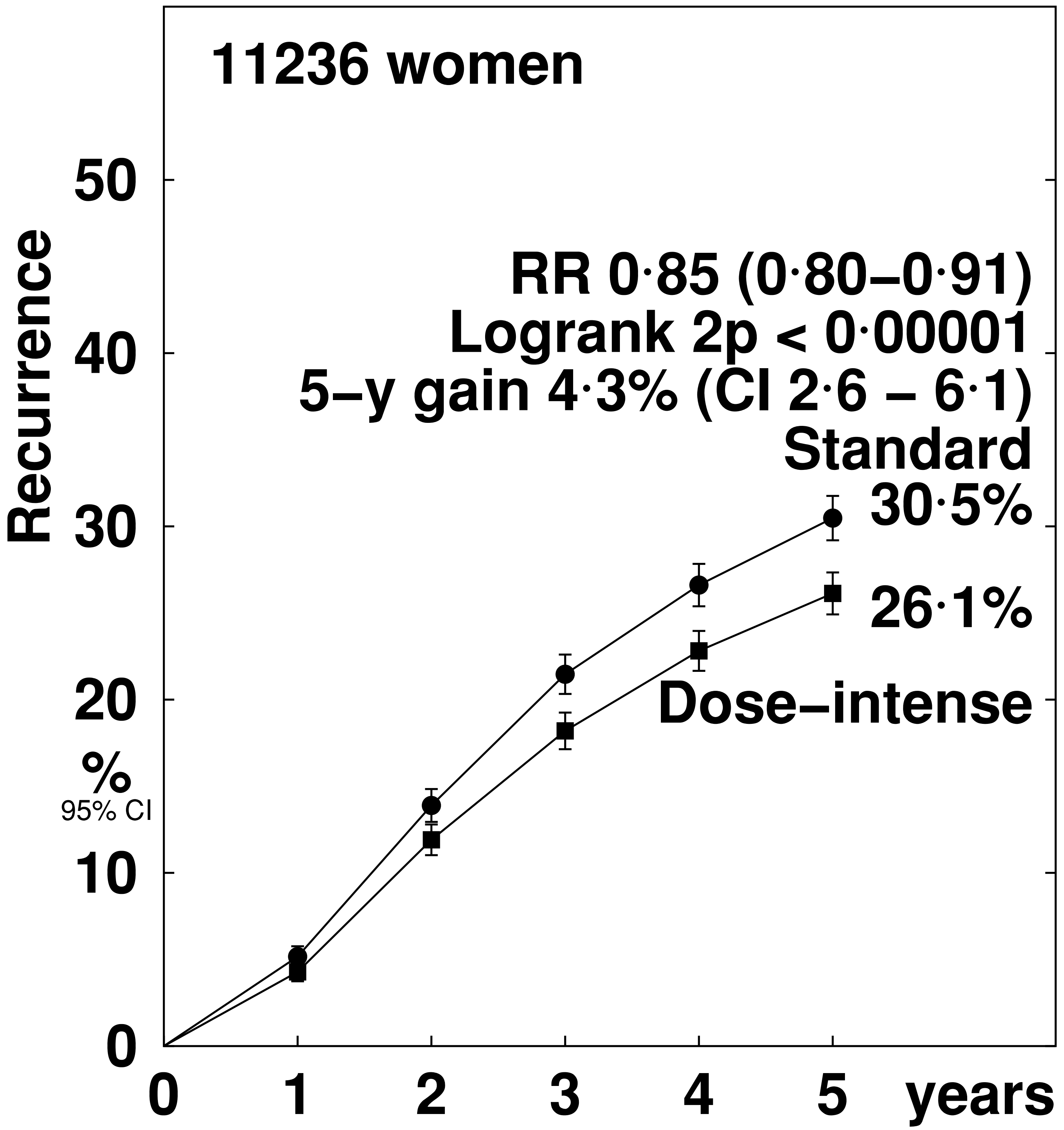

Recurrence rates (% / year) and logrank analyses

Allocation	Years 0 – 1	Years 2 – 4	Year 5+
Dose–intense	6·28 (662 / 10541)	5·95 (756 / 12706)	3·73 (414 / 11102)
Standard	7·37 (769 / 10440)	7·31 (886 / 12120)	3·95 (398 / 10073)
Rate ratio, from	0·84 CI 0·74 – 0·94	0·82 CI 0·73 – 0·91	0·94 CI 0·80 – 1·07
(O–E) / V	–53·1 / 308·8	–75·0 / 372·1	–12·5 / 185·8

P20: 10-year recurrence risk split by ER status and numbers of involved nodes (1-3 or 4+) in all trials of dose-intense vs standard schedule chemotherapy

ER−positive N1−3

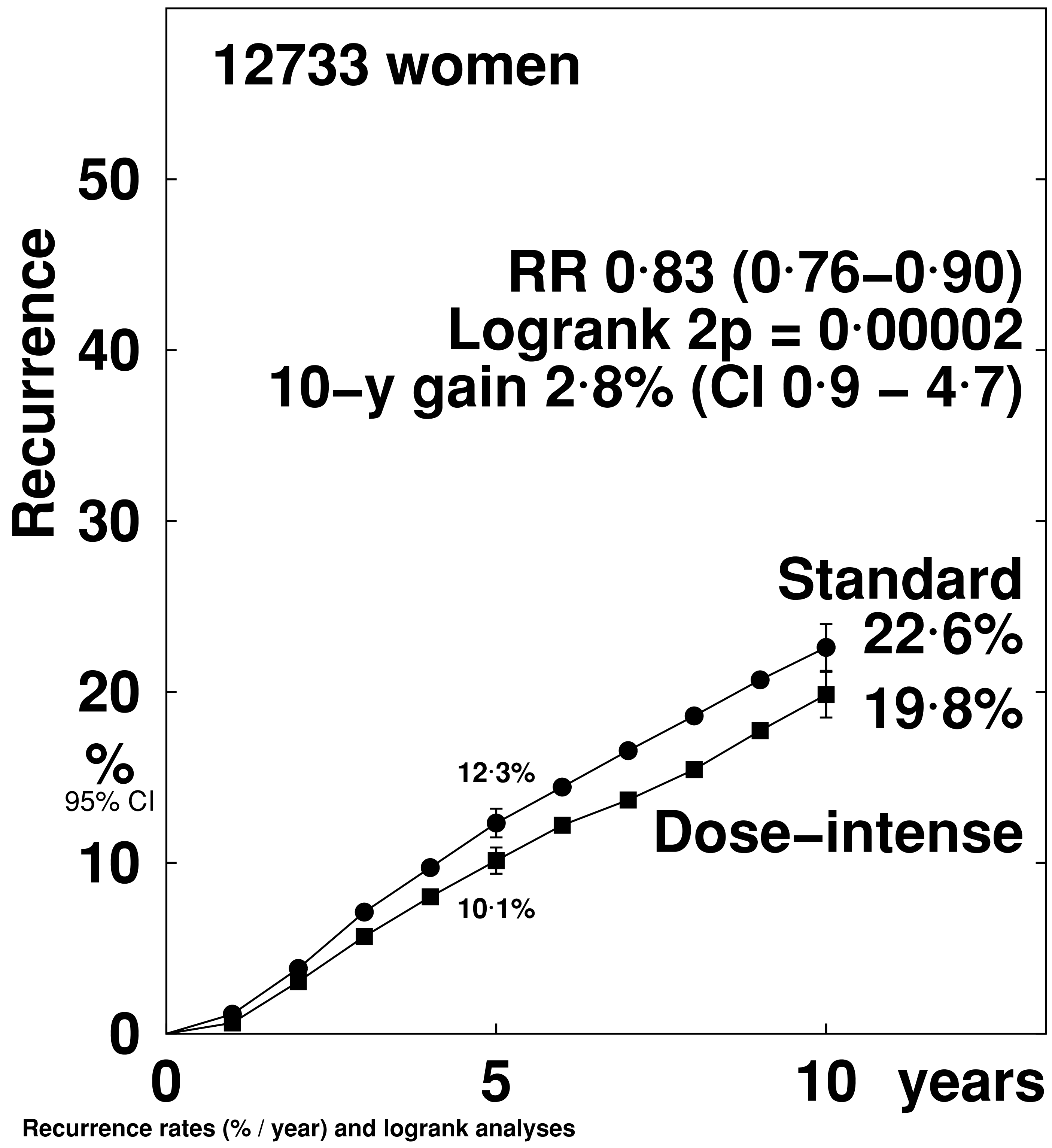

Recurrence rates (% / year) and logrank analyses

Allocation	Years 0 – 4	Years 5 – 9	Year 10+
Dose−intense	2·12 (622 / 29368)	2·20 (311 / 14120)	1·68 (64 / 3820)
Standard	2·60 (747 / 28726)	2·48 (336 / 13550)	2·16 (76 / 3526)
Rate ratio, from	0·81 CI 0·71 – 0·91	0·88 CI 0·73 – 1·03	0·78 CI 0·48 – 1·08
(O−E) / V	−69·6 / 328·4	−19·8 / 155·9	−8·4 / 34·1

ER−positive N4+

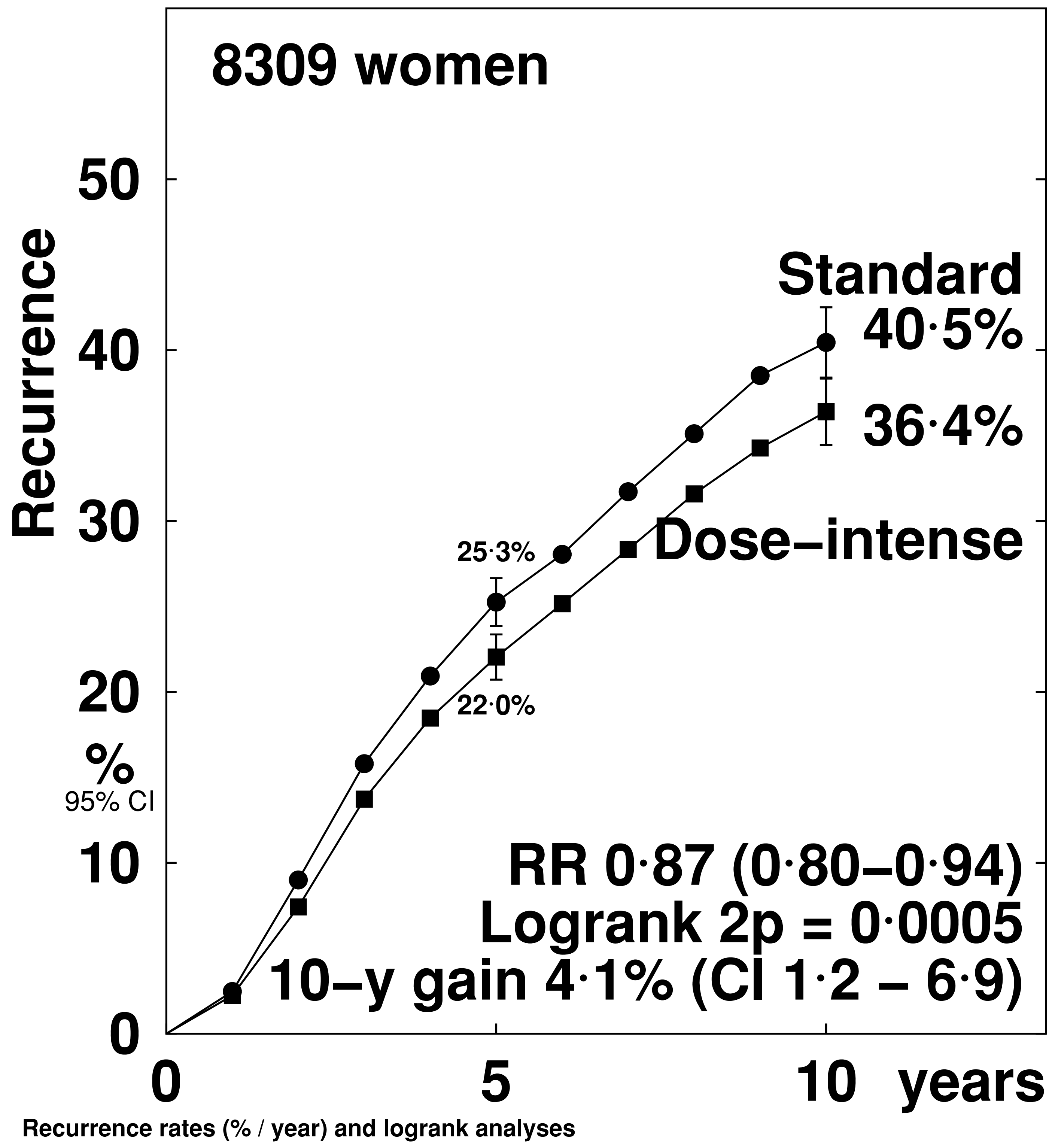

Recurrence rates (% / year) and logrank analyses

Allocation	Years 0 – 4	Years 5 – 9	Year 10+
Dose−intense	4·92 (884 / 17953)	4·18 (309 / 7393)	2·83 (29 / 1024)
Standard	5·75 (1002 / 17428)	4·56 (305 / 6686)	3·31 (31 / 937)
Rate ratio, from	0·85 CI 0·77 – 0·94	0·91 CI 0·76 – 1·07	0·86 CI 0·37 – 1·35
(O−E) / V	−69·2 / 433·7	−12·9 / 142·5	−2·0 / 13·8

ER−negative N1−3

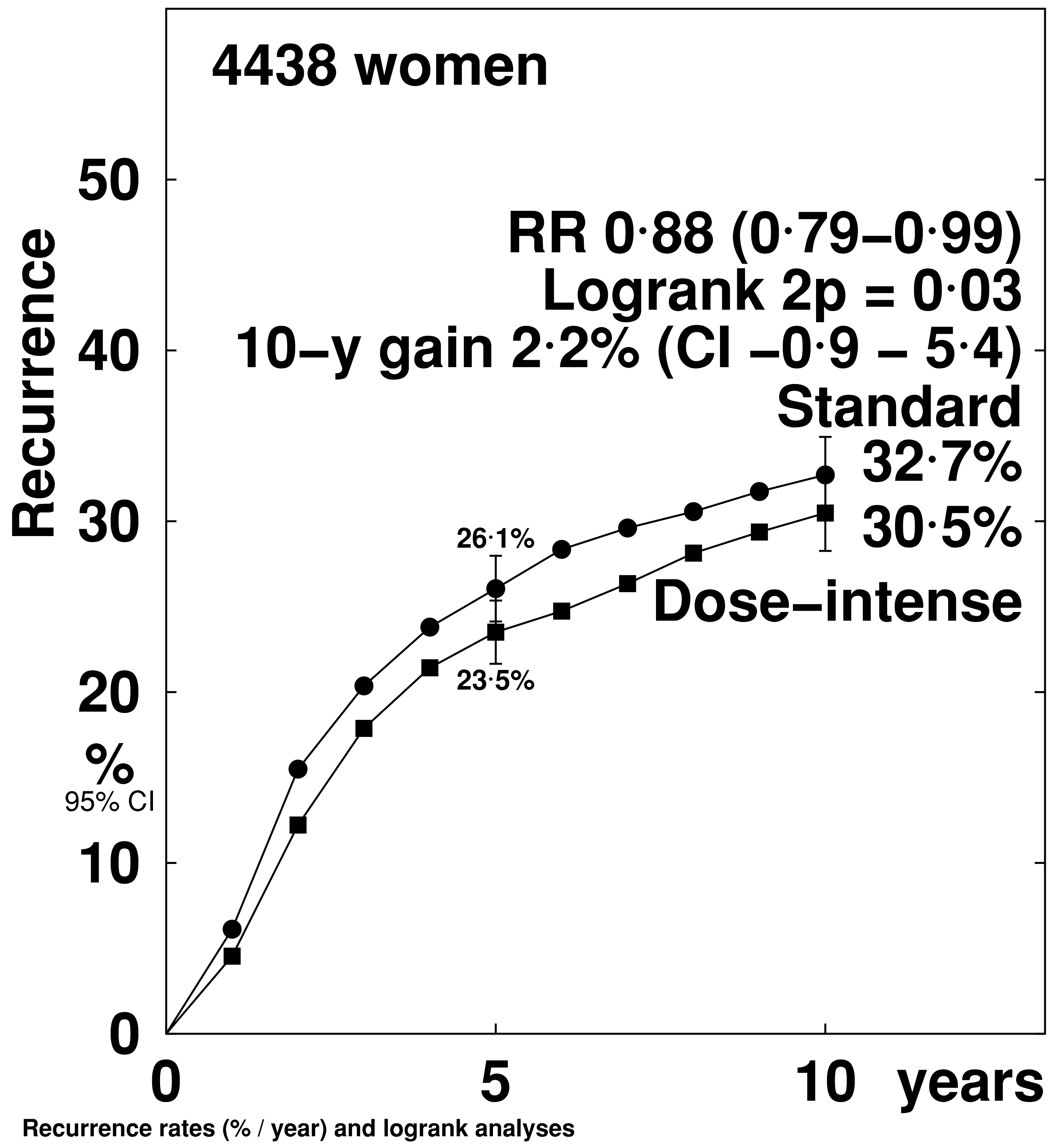

Recurrence rates (% / year) and logrank analyses

Allocation	Years 0 – 4	Years 5 – 9	Year 10+
Dose−intense	5·44 (501 / 9202)	1·92 (90 / 4690)	1·40 (26 / 1856)
Standard	6·27 (568 / 9066)	2·06 (94 / 4567)	1·76 (33 / 1872)
Rate ratio, from	0·87 CI 0·76 – 0·99	0·94 CI 0·66 – 1·23	0·80 CI 0·34 – 1·26
(O−E) / V	−32·8 / 245·0	−2·5 / 43·7	−3·2 / 14·4

ER−negative N4+

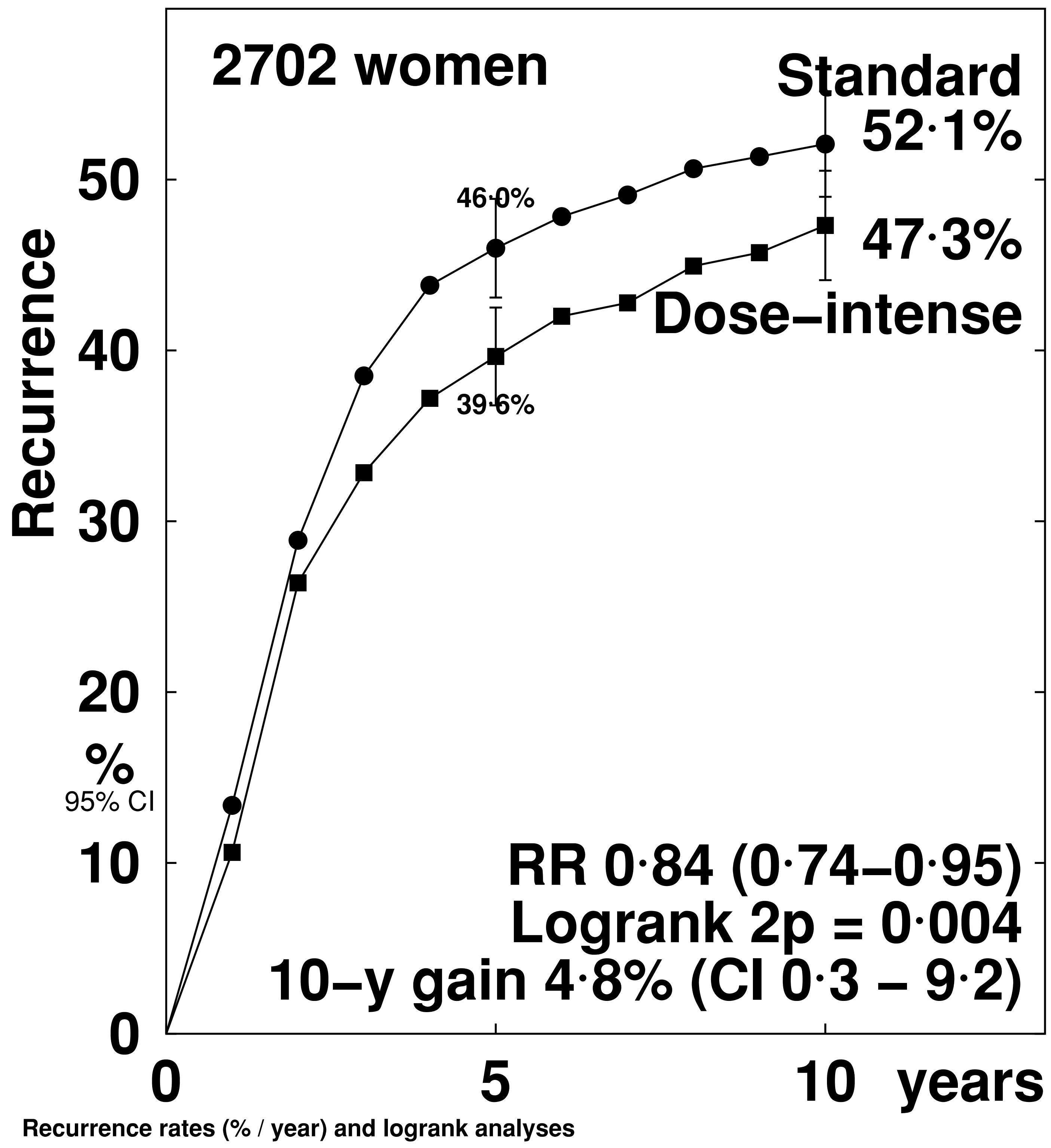

Recurrence rates (% / year) and logrank analyses

Allocation	Years 0 – 4	Years 5 – 9	Year 10+
Dose−intense	10·78 (516 / 4788)	2·87 (58 / 2021)	2·37 (12 / 506)
Standard	13·19 (616 / 4671)	2·57 (47 / 1830)	2·07 (10 / 483)
Rate ratio, from	0·81 CI 0·70 – 0·93	1·09 CI 0·66 – 1·52	1·18 CI 0·17 – 2·19
(O−E) / V	−49·0 / 233·3	2·0 / 22·9	0·8 / 4·5

P 21: 5-year recurrence split by tumour grade in all trials of dose-intense vs standard schedule chemotherapy

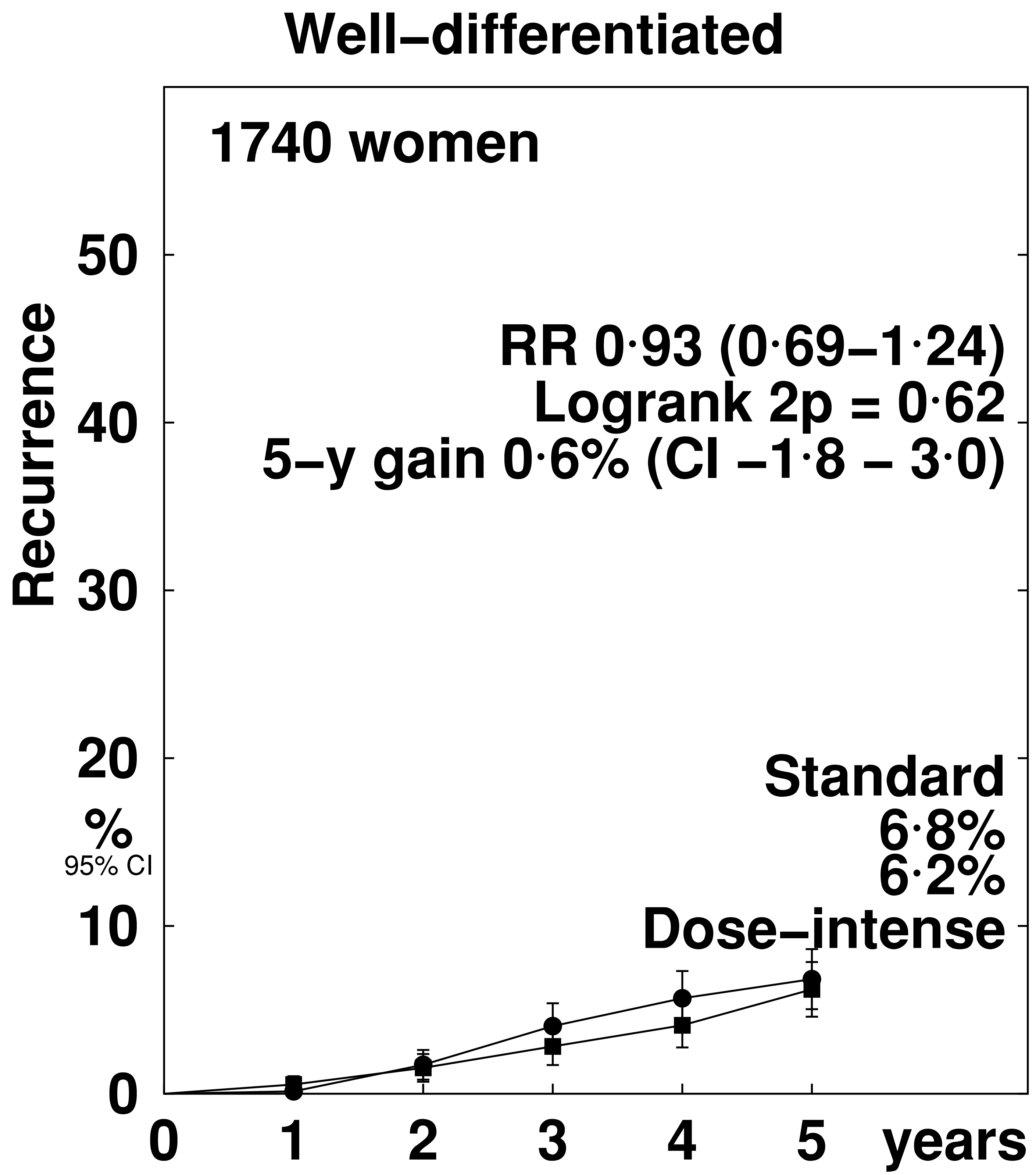

Recurrence rates (% / year) and logrank analyses

Allocation	Years 0 – 1	Years 2 – 4	Year 5+
Dose–intense	0·79 (14 / 1762)	1·54 (37 / 2405)	2·39 (44 / 1839)
Standard	0·84 (14 / 1658)	1·87 (42 / 2241)	2·35 (41 / 1744)
Rate ratio, from	0·86 CI 0·15 – 1·56	0·89 CI 0·46 – 1·32	1·00 CI 0·56 – 1·43
(O–E) / V	–1·0 / 6·6	–2·2 / 18·6	–0·1 / 20·0

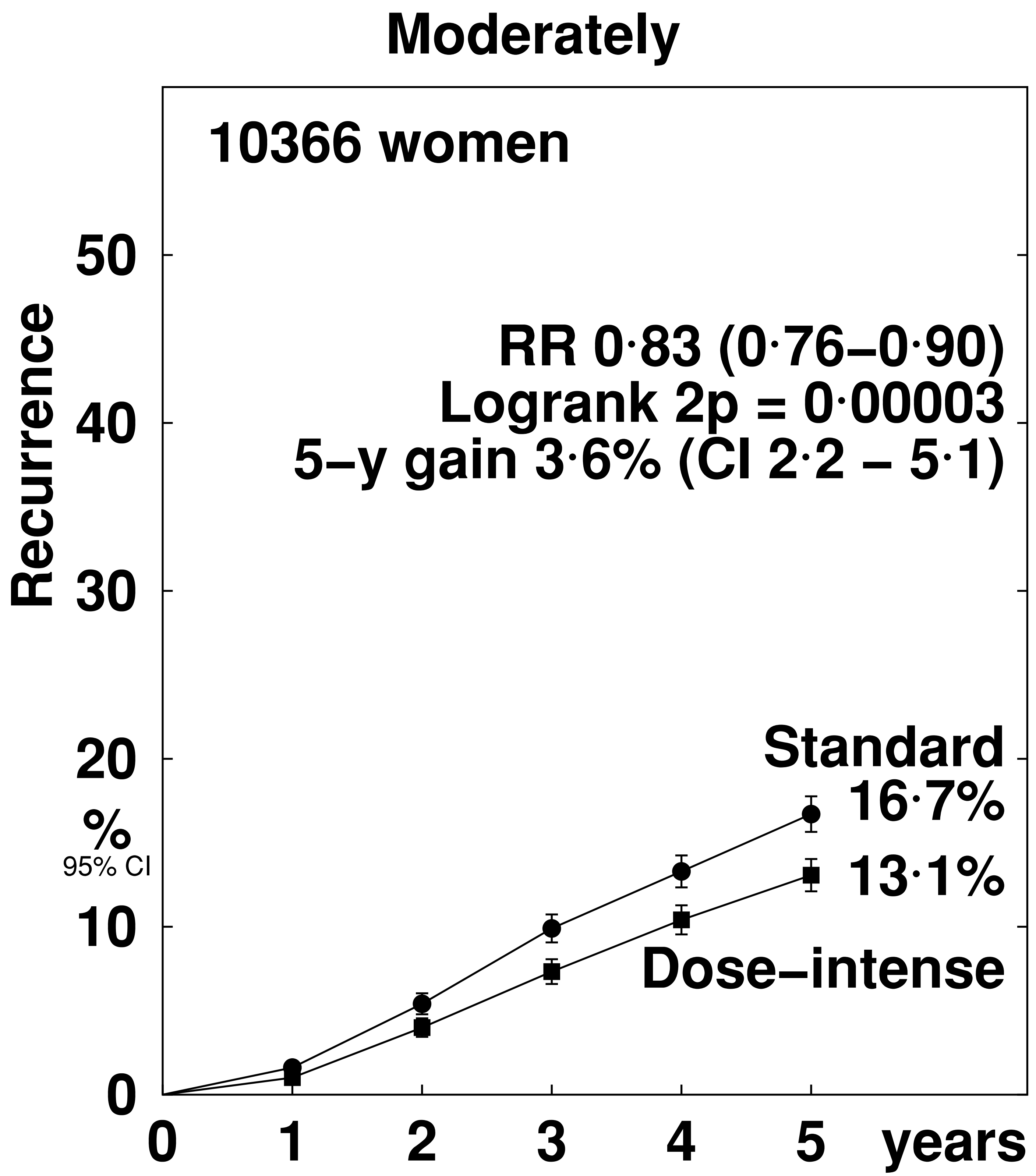

Recurrence rates (% / year) and logrank analyses

Allocation	Years 0 – 1	Years 2 – 4	Year 5+
Dose–intense	2·07 (209 / 10075)	3·35 (447 / 13327)	2·92 (314 / 10760)
Standard	2·71 (271 / 9993)	4·24 (548 / 12926)	2·86 (290 / 10144)
Rate ratio, from	0·73 CI 0·57 – 0·89	0·78 CI 0·66 – 0·89	1·01 CI 0·85 – 1·18
(O–E) / V	–36·0 / 114·1	–59·3 / 235·5	2·0 / 144·3

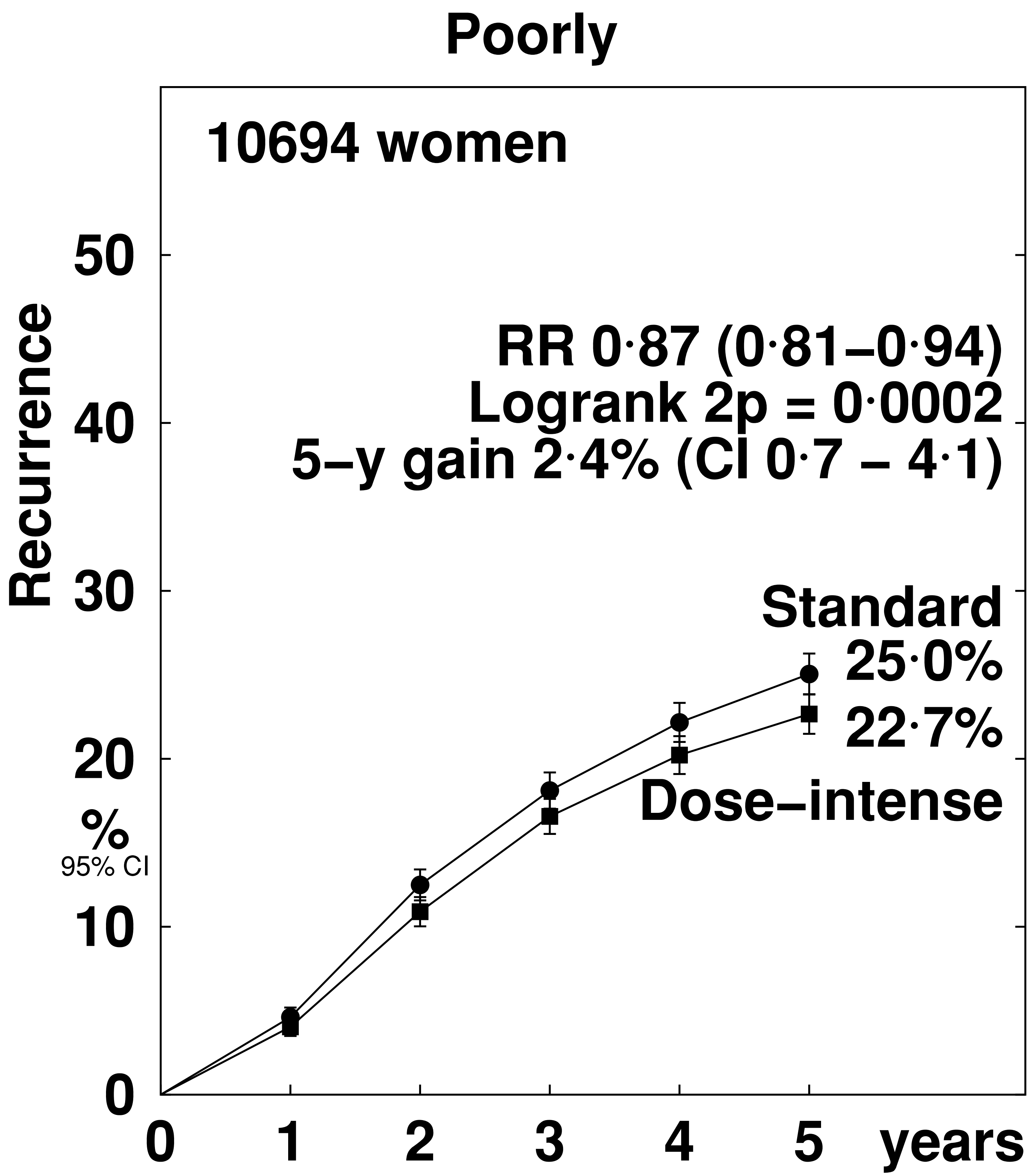

Recurrence rates (% / year) and logrank analyses

Allocation	Years 0 – 1	Years 2 – 4	Year 5+
Dose–intense	5·74 (576 / 10041)	4·82 (595 / 12353)	2·39 (224 / 9375)
Standard	6·57 (660 / 10052)	5·26 (639 / 12148)	3·06 (277 / 9053)
Rate ratio, from	0·86 CI 0·75 – 0·97	0·92 CI 0·81 – 1·03	0·78 CI 0·62 – 0·94
(O–E) / V	–42·3 / 282·4	–25·0 / 287·3	–29·3 / 119·5

P22: Mortality by cause and incidence of second cancers in all trials of dose-intense vs standard schedule chemotherapy

	Events		(O-E)	Variance	Rate Ratio & SE		p
	Dose intense (n=18623)	Standard (n=18750)					
Death without recurrence	540	584	-34.6	266.5	0.88	0.06	0.03
Death with recurrence	2890	3286	-199.4	1431.3	0.87	0.02	<0.00001
Any death	3430	3870	-234.0	1697.8	0.87	0.02	<0.00001
**Death without recurrence (selected groups of causes)**							
**Vascular disease:**							
Stroke	10	8	0.6	4.4	1.15	0.51	0.78
Pulmonary embolus	0	2	-1.1	0.5	0.11	0.57	0.12
Heart & other vascular	17	18	-0.3	8.6	0.97	0.33	0.92
**Other primary cancer:**							
Acute myeloid leukaemia	33	27	2.4	14.8	1.18	0.28	0.53
Other cancer	56	66	-6.0	30.0	0.82	0.17	0.27
Other specified cause	163	185	-15.1	82.5	0.83	0.10	0.10
Unknown cause	264	278	-15.0	126.3	0.89	0.08	0.18
**Death without recurrence**							
By entry age (years) %/woman-years							
<55	0.27 (227/84901)	0.30 (253/83152)	-18.8	116.7	0.85	0.09	0.08
55 – 69	0.68 (274/40589)	0.73 (289/39666)	-13.5	134.1	0.90	0.08	0.24
≥70	2.01 (38/1888)	2.44 (42/1718)	-2.8	15.7	0.84	0.23	0.48
All ages	0.42 (540/127379)	0.47 (584/124536)	-34.6	266.8	0.88	0.06	0.03
**Death without recurrence**							
During the first year							
By entry age (years) %/woman-years							
<55	0.13 (15/11696)	0.26 (29/11686)	-7.4	10.8	0.50	0.22	0.02
55 – 69	0.57 (33/5825)	0.61 (36/5857)	-2.0	17.0	0.89	0.23	0.63
≥70	1.72 (4/233)	2.54 (6/236)	-1.7	2.1	0.45	0.47	0.24
All ages	0.30 (53/17755)	0.40 (71/17779)	-10.6	30.1	0.70	0.15	0.05
**Second cancer incidence**							
Contralateral breast							
By entry age (years) %/woman-years							
<55	0.19 (158/84890)	0.25 (204/83152)	-26.6	87.5	0.74	0.09	0.004
55 – 69	0.19 (78/40589)	0.22 (89/39659)	-5.8	40.1	0.87	0.15	0.36
≥70	0.11 (2/1887)	0.41 (7/1718)	-1.5	1.9	0.45	0.50	0.28
All ages	0.19 (238/127366)	0.24 (300/124529)	-33.9	129.4	0.77	0.08	0.003
**Second primary cancer as first event**							
By entry age (years) %/woman-years							
<55	0.29 (245/83974)	0.30 (243/82320)	-1.8	120.2	0.99	0.09	0.87
55 – 69	0.58 (230/39973)	0.57 (222/39099)	-1.7	109.5	0.99	0.10	0.87
≥70	1.14 (21/1838)	0.77 (13/1693)	2.9	7.4	1.48	0.45	0.29
All ages	0.35 (496/125785)	0.35 (478/123112)	-0.6	237.1	1.00	0.06	0.97

P23: Sensitivity analyses for death without recurrence (all trials), by tumour size and nodal status

Category	Events/Women: Allocated Dose-intense	Events/Women: Allocated Standard	Dose-intense events: Logrank O−E	Dose-intense events: Variance of O−E	Ratio (& CI)
**(a) Tumour size (trend χ^2_1 = 0.4; 2p > 0.1; NS)**					
**1 – 20 mm (T1)**	178/6967 (2·6%)	203/7169 (2·8%)	−14·3	92·3	**0·86 (0·66 – 1·12)**
**21 – 50 mm (T2)**	277/8686 (3·2%)	293/8631 (3·4%)	−13·9	139·1	**0·91 (0·73 – 1·13)**
**> 50 mm (T3/T4)**	33/1200 (2·8%)	31/1166 (2·7%)	0·5	13·9	
**Other / unknown**	52/1770 (2·9%)	57/1784 (3·2%)	−3·7	24·6	
**(b) Nodal status (trend χ^2_1 = 0.3; 2p > 0.1; NS)**					
**N0**	105/2552 (4·1%)	111/2623 (4·2%)	−4·2	51·8	
**N1−3**	247/8738 (2·8%)	262/8746 (3·0%)	−11·9	123·1	**0·91 (0·72 – 1·14)**
**N4+**	136/5617 (2·4%)	154/5661 (2·7%)	−12·5	68·0	**0·83 (0·61 – 1·14)**
**N unknown**	52/1716 (3·0%)	57/1720 (3·3%)	−6·0	23·5	
**Total**	**540/ 18623 (2·9%)**	**584/ 18750 (3·1%)**	**−34·6**	**266·5**	**0·878 (0·779 – 0·990)** 2p = 0·03

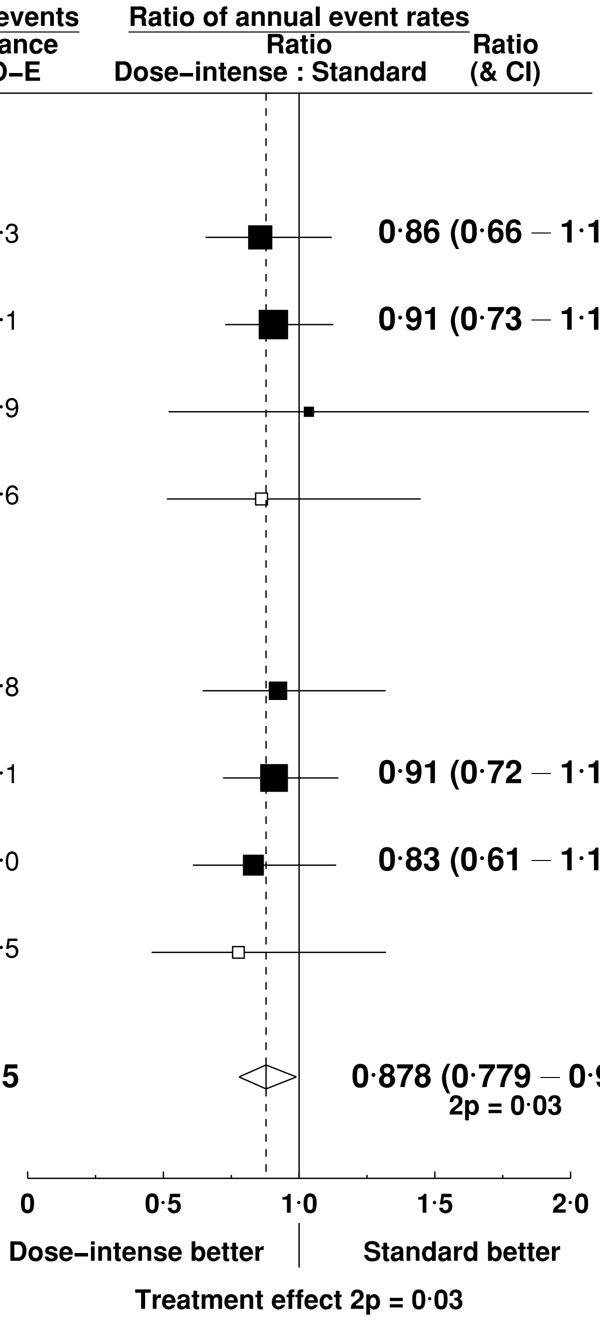

99% or 95% confidence intervals

P24-30: Published data — toxicity of chemotherapy

Note: Data in the following tables are reported as % of patients unless otherwise specified. **Dash** indicates not reported. Significance test for the difference between the comparison groups was not reported unless otherwise specified in **bold** indicating a statistically significant difference or in *Italic* indicating no significant difference.
Abbreviations: Con = control. Conc = concurrent. DD = dose dense. FN = febrile neutropenia. NS = no statistically significant difference. Seq = sequential. TCP = thrombocytopenia.

(A1) Shorter interval between cycles (same drugs, doses and number of cycles in each arm) — Toxicity of grade 3 or above ¥

Year code	Study name	Anaemia		Leukopenia *		TCP		FN‡		Stomatitis		Diarrhoea		Vomiting**		Asthenia		Transaminase ƚ		Neuropathy (sensory)≠		Death due to toxicity	
		DD	Con	DD	Con	DD	Con	DD	Con	DD	Con	DD	Con	DD	Con	DD	Con	DD	Con	DD	Con	DD	Con
92E	GONO Italy	3	<2	3	9	1	<1	<1	<1	3	1	<1	<1	12	11	1	<1	1	<1	-	-	-	-
92J	PisaGenoa	4.4	0	5.4	5.4	<1	<1	-	-	1.4	0	-	-	7.4	10.8	-	-	-	-	-	-	-	-
97D	CALGB 9741 (Seq)	<1	<1	<1	1	<1	<1	2	3	1	1	3	1	4	3	-	-	-	-	4	4	0.20	0.62
97D	CALGB 9741 (Conc)	<1	<1	6	12	<1	<1	2	6	3	3	1	2	6	8	-	-	-	-	4	5	0	0.40
97X	Bayreuth	-	-	-	-	-	-	-	-	-	-	-	-	-	-	-	-	-	-	-	-	0	0
03D	RM NHST London 4AC(q2v3w)	9	0	0	2	2	0	-	-	*10*	*7*	0	5	5	5	17	7	-	-	-	-	0	0
	RM NHST London 4EC(q2v3w)	0	0	0	5	0	0	-	-	0	0	0	5	0	0	9	0	-	-	-	-	0	0
$03Q_{1-4}$	GIM 2 Italy (EC-P)	1	0	10	37	<1	<1	-	-	1	0	<1	<1	1	1	3	1	2	1	4	3	0	0
$03Q_{1-4}$	GIM 2 Italy (FEC-P)	2	<1	20	48	1	<1	-	-	1	1	1	<1	4	2	3	2	2	<1	3	2	0	0
$03Q_{1-4}$	GIM2 Italy (overall)	**1**	**0**	**15**	**44**	*1*	*<1*	-	-	*1*	*<1*	*<1*	*<1*	*3*	*2*	*3*	*2*	**2**	**<1**	*3*	*2*	0	0
04)	CAMs Beijing	0	0	**16**	**55**	0	0	-	-	0	0	0	0	*16*	*9.8*	-	-	*4*	*0*	0	0	0	0
05P	TACT2	*2.3*	*1.2*	*10.5*	*10.9*	*0.9*	*1.1*	8.6	7.7	*4.1*	*3.6*	*7.1*	*5.8*	*3.5*	*4.2*	*11.1*	*11.1*	*0.3*	*0.6*	*0.3*	*0.5*	-	-

¥ For CALGB 9741 it was based on 1962 (of the total of 2005) patients with available data. For TACT2 it was based on 2115 QOL sub-study patients (of the total of 4391) during cycle 1 to 8.
* For GIM2 Italy and CAMS Beijing values were for neutropenia as leukopenia not reported separately.
‡ For CALGB 9741 data were based on 421 patients with data available and were reported as febrile neutropenia resulting in hospitalization.
** Reported as nausea/vomiting for GONO Italy, PisaGenoa, and RM NHST London. Reported as gastrointestinal reaction for CAMs Beijing.
ƚ Reported as transaminase and/or bilirubin for the CAMs Beijing trial.
≠ Reported as neuropathy overall (not specifically for sensory) for the GIM2 Italy trial.

(A1) Shorter interval between cycles (same drugs, doses and number of cycles in each arm) — Compliance

Year code	Trial name	Completed all cycles		Dose reduction		Dose delay	
		DD	Con	DD	Con	DD	Con
92E	GONO Italy	93.4	93.8	2.5	2.0	19	27
92J	PisaGenoa	93 (no modification or delay)	95 (no modification or delay)	-	-	-	-
97D	CALGB 9741* (sequence)	-	-	3 –7	1 – 7	Patients %: 45<br>Cycles %: 7	Patients %: 39<br>Cycles %: 7
97D	CALGB 9741 * (concurrent)	100	100	3 – 5	1 – 5	Patients %: 31<br>Cycles %: 6	Patients %: 39<br>Cycles %: 8
97X	Bayreuth	-	-	-	-	-	-
03D	RM NHST London (4AC(q2v3w))	98	98	2	5	7	7
	RM NHST London (4EC(q2v3w))	96	100	0	0	17	5
$03Q_{1-4}$	GIM 2 Italy (EC-P)	90	87	3.6	2.9	1.4	0.8
$03Q_{1-4}$	GIM 2 Italy (FEC-P)	88	89	3.8	2.9	0.6	0.4
04)	CAMs Beijing	98	96	-	-	**2.4** (% of cycles)	**6** (% of cycles)
05P	TACT2	85.1	85	-	-	-	-

* CALGB 9741: for data on completed all cycles it was based on 1962 patients with available data; for data on dose reduction it is unclear whether it was reported as % of patients or % cycles.

(A2) Shorter interval between cycles (but additional drugs in control arm) — Toxicity of grade 3 or above

Year code	Study name	Anaemia		Leukopenia *		TCP		FN		Stomatitis		Diarrhoea		Vomiting		Asthenia		Transaminase		Neuropathy (sensory)		Death due to toxicity	
		DD	Con	DD	Con	DD	Con	DD	Con	DD	Con	DD	Con	DD	Con	DD	Con	DD	Con	DD	Con	DD	Con
93#	EORTC MA.10	50.9	16.3	78.1	85.5	33.1	20.8	8.4	14	17	14.9	-	-	18.8	10.9	7.1	6.3	-	-	-	-	0	0.89
93W	Hamburg ł	-	-	-	-	-	-	-	-	-	-	-	-	-	-	-	-	-	-	-	-	-	-
99Y	GEPARDUO	-	-	74.2	53.7	1.8	0	4.6	3.1	8.0	3.8	4.0	7.6	14.6	10.0	22.2	28.3	-	-	-	-	-	-
00Y	GASD Germany	-	-	-	-	-	-	<1	<1	8	3	-	-	<1	<1	-	-	-	-	-	-	-	-
07B	SBG 2004-1 Panther	4.6	1.1	90.7	81.0	4.6	1.9	11.5	15.4	7.7	3.3	4.4	4.0	3.6	2.6	21.6	10.6	-	-	3.3	1.3	Overall 0.09	

* Reported as neutropenia for EORTC MA.10.

ł The Hamburg trial: it was stated that toxicity measured with standardised methods were similar in both arms.

(A2) Shorter interval between cycles (but additional drugs in control arm) — Compliance

Year code	Trial name	Completed all cycles		Dose reduction		Dose delay	
		DD	Con	DD	Con	DD	Con
93#	EORTC MA.10	85.3	81.4	-	-	-	-
93W	Hamburg	-	-	-	-	-	-
99Y	GEPARDUO*	76.8	92.4	-	-	-	-
00Y	GASD Germany	-	-	-	-	-	-
07B	SBG 2004-1 Panther	83.1	94.8	-	-	-	-

* GEPARDUO: dose reduction was not permitted.

(A3) Shorter interval between cycles (plus additional treatment in dose-dense arm) — Toxicity of grade 3 or above

Year code	Study name	Anaemia		Leukopenia *		TCP		FN		Stomatitis		Diarrhoea		Vomiting		Asthenia		Transaminase		Neuropathy (sensory)		Death due to toxicity	
		DD	Con	DD	Con	DD	Con	DD	Con	DD	Con	DD	Con	DD	Con	DD	Con	DD	Con	DD	Con	DD	Con
00F≠	MA21	29.0	1	55.3	42.6	24.1	1.5	16.1	4.8	9.9	0.7	3.6	1.2	15.0	6.2	-	-	-	-	6.0	5.5	-	-
02C	PREPARE	*0.6*	*0*	*0.6*	*0.3*	*0.3*	*0.3*	-	-	**4.0**	**0.8**	*0.3*	*0.3*	*4.8*	*4.4*	-	-	-	-	**8.9**	**2.5**	0	0

* Reported as granulocytes for MA 21. ≠ Reported as incidence among patients with toxicity data rather than among all patients in the trial.

(A3) Shorter interval between cycles (plus additional treatment in dose-dense arm) — Compliance

Year code	Trial name	Completed all cycles		Dose reduction		Dose delay	
		DD	Con	DD	Con	DD	Con
00F	MA21	83	90	-	-	-	-
02C	PREPARE	**81.8**	**83.8**	-	-	-	-

(B1) Sequential vs concurrent anthracycline/taxane usage (same drugs in each arm) — Toxicity of grade 3 or above

Year code	Trial name	Anaemia		Leukopenia *		TCP		FN		Stomatitis		Diarrhoea		Vomiting		Asthenia		Transaminase		Neuropathy (sensory)		Death due to toxicity	
		Seq	Conc	Seq	Conc	Seq	Conc	Seq	Conc	Seq	Conc	Seq	Conc	Seq	Conc	Seq	Conc	Seq	Conc	Seq	Conc	Seq	Conc
$98D_{1+2}$	BIG 02-98	3	3	-	-	2	2	**8**	**12**	7	4	3	3	-	-	7	6	-	-	0.6	0.2	0.14	0.14
99G	NSABP B-30	-	-	-	-	-	-	22	16	5	2	4	6	8	7	12	10	-	-	-	-	<1	<1
00H	BCIRG 005	*2.0*	*2.9*	*57.8*	*59.9*	*1.3*	*2.5*	**7.7**	**17.4**	2.9	2.6	*3.1*	*2.9*	*5.2*	*4.1*	*6.3*	*5.1*	-	-	**1.5**	**0.3**	0.12	0.24
01=	CT/01.04	-	-	-	-	-	-	-	-	-	-	-	-	-	-	-	-	-	-	-	-	0	0
03K	Fudan CHN TAX619ǂ	*3.7*	*1.6*	*47.7*	*55.6*	-	-	*15.0*	*16.1*	-	-	*3.7*	*2.4*	*10.3*	*4.0*	*6.5*	*13.7*	-	-	-	-	-	-
06&	Sanofi Aventis ≠	-	-	25.6	37.3	5.1	14.7	7.7	18.7	6.4	2.7	5.1	6.7	-	-	15.4	8	-	-	-	-	-	-
06T	BOOG 2007-02	1	1	20	0	5	0	23	9	3	1	4	0	1	0	10	4	-	-	5	0	0	0

* Reported as neutropenia for BCIRG 005, Fudan CHN TAX619 and BOOG 2007-02. Neutropenia is also been reported for some of the rest of trials.

ǂ The data reported here are from an interim analysis based on a total of 231 women (107 women in the Seq arm and 124 women in the Conc arm) who completed their study treatment, among all 470 patients who were enrolled up to May 2005.

≠ Sanofi Aventis: grade ≥3 treatment emergent adverse events, that occurred regardless of relationship to study drug, and reported by ≥5 % of patients in any arm.

(B1) Sequential vs concurrent anthracycline/taxane usage (same drugs in each arm) — Compliance

Year code	Trial name	Completed all cycles		Dose reduction		Dose delay	
		Seq	Conc	Seq	Conc	Seq	Conc
$98D_{1+2}$	BIG 02-98	91	94	25	20	-	-
99G	NSABP B-30	86	97	-	-	53	24
00H	BCIRG 005	90	93	6	8	21	24
01=	CT/01.04	-	-	-	-	-	-
03K	Fudan CHN TAX619	-	-	-	-	-	-
06&	Sanofi Aventis	48.7	48	-	-	-	-
06T	BOOG 2007-02 *	-	-	-	-	-	-

*BOOG 2007-02: stated that dose reductions/delays were infrequent. Most patients received planned number of chemo cycles.

(B2) Sequential vs concurrent anthracycline/taxane usage (but some difference in drugs between arms) — Toxicity of grade 3 or above

Year code	Trial name	Anaemia		Leukopenia *		TCP		FN		Stomatitis		Diarrhoea		Vomiting		Asthenia		Transaminase		Neuropathy (sensory)		Death due to toxicity	
		Seq	Conc	Seq	Conc	Seq	Conc	Seq	Conc	Seq	Conc	Seq	Conc	Seq	Conc	Seq	Conc	Seq	Conc	Seq	Conc	Seq	Conc
00)	Pfizer NCT00140075	-	-	-	-	-	-	-	-	-	-	-	-	-	-	-	-	-	-	-	-	-	-
04F	GEICAM 2003-10	-	-	**18.9**	**9.8**	-	-	*6.6*	*6.8*	*6.0*	*5.1*	**2.8**	**11.0**	*5.2*	*4.5*	*12.7*	*10.7*	-	-	-	-	0.1	0.1

* Reported as neutropenia for GEICAM 2003-10.

(B2) Sequential vs concurrent anthracycline/taxane usage (but some difference in drugs between arms) — Compliance

Year code	Trial name	Completed all cycles		Dose reduction		Dose delay	
		Seq	Conc	Seq	Conc	Seq	Conc
00)	Pfizer NCT00140075	-	-	-	-	-	-
04F	GEICAM 2003-10	94.8	87.4	-	-	-	-

(C) Shorter interval between cycles AND sequential anthracycline/taxane usage — Toxicity of grade 3 or above

Year code	Trial name	Anaemia		Leukopenia *		TCP		FN		Stomatitis		Diarrhoea		Vomiting **		Asthenia		Transaminase		Neuropathy (sensory)		Death due to toxicity ᵻ	
		DD	Con	DD	Con	DD	Con	DD	Con	DD	Con	DD	Con	DD	Con	DD	Con	DD	Con	DD	Con	DD	Con
98Q	AGO Germany	<1	0	*54*	*59*	**5**	**1**			**11**	**2**	*2*	*1*	**<9**	**<4**	-	-	-	-	**<7**	**<1**	**-**	**-**
$98V_1$	ETC Germany	9	1	70	48	9	1	7	2	9	2	3	1	6	4	-	-	-	-	7	4	0	0
00X	HE10/00	*1.7*	*0.6*	*11.3*	10.7	*1.1*	*?*	*4.6*	*5.9*	2.4	2.3	-	-	2.8	3.1	?	?	-	-	**9.5**	**2.1**	0.18	0
03L	AERO B-03 (EC;T v TEC)	3	0	-	-	0	3	10	11	0	0	-	-	10	9	-	-	-	-	*10*	*0*	0	0
03L	AERO B-03 (T;EC v TEC)	3	0	-	-	3	3	3	11	0	0	-	-	6	9	-	-	-	-	*9*	*0*	0	0
04=	SBG 2004-1 feasibility (escalating) ǂ	-	-	-	-	-	-	9.5	7.5	4.8	0	4.8	12.5	4.8	0	28.6	12.5	-	-	4.8	2.5	0	0
	SBG 2004-1 feasibility (fixed) ǂ	-	-	-	-	-	-	0	7.5	0	0	2.4	12.5	0	0	11.9	12.5	-	-	2.4	2.5	0	0
04D	NSABP-B38	2	<1	-	-	-	-	3	9	<1	<1	2	7	3	3	8	9	-	-	7	<1	0.74	0.80

* Reported as neutropenia for AGO Germany and AERO B-03.

? It is unclear for those without a given value whether it refers to zero or not reported.

** Reported as nausea/vomiting for AGO Germany and HE10/00.

ᵻ For NSABP-B38: death due to toxicity for the sequential group was 0.31% in the AC;P sub-group and 0.43% in the AC;P plus Gemcitabine sub-group.

ǂ For the SBG 2004-1 feasibility trial, there were no cases of clinically diagnosed cardiotoxicity or hematologic malignancies at long-term follow-up (a median of 10.3 years).

(C) Shorter interval between cycles AND sequential anthracycline/taxane usage — Compliance

Year code	Trial name	Completed all cycles		Dose reduction ‡		Dose delay *	
		DD	Con	DD	Con	DD	Con
98Q	AGO Germany ≠	-	-	5	1	17	10
$98V_1$	ETC Germany	84	91	-	-	16	12
00X	HE10/00	-	-	-	-	**21**	**17**
03L	AERO B-03 (EC;T v TEC)	80.6	94.3	37	17	53	11
03L	AERO B-03 (T;EC v TEC)	91.2	94.3	32	17	38	11
04=	SBG 2004-1 feasibility (escalating)	86	90	-	-	24	3
	SBG 2004-1 feasibility (fixed)	90	90	-	-	21	3
04D	NSABP-B38	88	91	-	-	-	-

≠ AGO Germany: stated that 86% patients in the dose-dense group and 95% patients in the control group completed the planed number of preoperative chemotherapy cycles.

‡ Reported as patients had at least one dose reduction for the AERO B-03 trial.

* Reported as 'patients had at least one cycle delayed by >7 days' for the AERO B-03 trial, 'delayed due to side-effects' for the SBG 2004-1 feasibility trial, and '% of cycles' for the ETC Germany trial. For the ETC Germany trial: more than 50% of delays were reported to be caused by logistical problems (eg, bank holidays) or patient preference rather than required by toxicity.

(D) Same total dose in fewer cycles — Toxicity of grade 3 or above

Year code	Trial name	Anaemia		Leukopenia		TCP		FN		Stomatitis		Diarrhoea		Vomiting		Asthenia		Transaminase		Neuropathy (sensory)		Death due to toxicity	
		DD	Con	DD	Con	DD	Con	DD	Con	DD	Con	DD	Con	DD	Con	DD	Con	DD	Con	DD	Con	DD	Con
85A	CALGB CLB-8541	-	-	-	-	-	-	-	-	-	-	-	-	-	-	-	-	-	-	-	-	-	-
$88T_1$	NCC Tokyo *	0	2	1	1	-	-	-	-	2	2	2	0	30	25	-	-	-	-	-	-	0	0
94L	SWOG 9313 **	-	-	29	50	4	10	-	-	2	5	<1	1	13	10	4	6	-	-	-	-	NS	NS

* NCC Tokyo data: grade 2 anaemia; grade 3-4 leukopenia; non-specified grade of stomatitis; non-specified grade of diarrhoea; grade 3-4 nausea/vomiting. Stated that both treatment regimens were well tolerated by the patients enrolled in this study.

** For the 94L SWOG 9313 trial: data reported for the Leukopenia and TCP were grade 4, and for the rest were grad 3 and 4. For asthenia it was reported as malaise/fatigue/lethargy. No significant difference between the two treatments in deaths of different causes.

(D) Same total dose in fewer cycles — Compliance

Year code	Trial name	Completed all cycles		Dose reduction		Dose delay	
		DD	Con	DD	Con	DD	Con
85A	CALGB CLB-8541	-	-	-	-	-	-
$88T_1$	NCC Tokyo	*3.2**	*8.2**	-	-	-	-
94L	SWOG 9313	>80 received at least 95% of planed dose-intensity	>80 and 76% received at least 95% of planed dose-intensity of anthracycline and cyclophosphamide respectively	-	-	-	-

* NCC Tokyo: reported as % of patients refused to continue to receive chemotherapy (p = 0.12).

P31-32: Published data – cardiotoxicity

<table>
<tr><th>Year code</th><th>Trial name</th><th>Cardiotoxicity data reported in publications</th></tr>
<tr><td colspan="3">(A1) Shorter interval between cycles (same drugs, doses and number of cycles in each arm)</td></tr>
<tr><td>92E</td><td>GONO MIG1 Italy</td><td>Grade 2 cardiotoxicity occurred in one patient (0.2%) in each arm. No grade 0, 1, 3 and 4 cardiotoxicity in both arms.</td></tr>
<tr><td>92J</td><td>Pisa/Genoa Italy</td><td>No specific cardiac toxicities were mentioned. The authors stated that chemotherapy-related toxicities were mild and superimposable in the two arms; no patient was admitted for treatment-related toxicity; accelerated chemotherapy reduced the duration of the combined-modality program (6.1 versus 4.6 months) with no additional toxicities.</td></tr>
<tr><td>97D</td><td>CALGB 9741 USA</td><td>One death due to doxorubicin-related cardiomyopathy and heart failure occurred in the treatment group arm of 3-weekly sequential doxorubicin then paclitaxel then cyclophosphamide regimen, 30 months after the beginning of treatment. Less than 2% of patients reported late significant cardiac toxicity requiring treatment. Patients receiving the 3-weekly regimens had a slightly higher incidence of late cardiotoxicity than those receiving the 2-weekly regimens (2% v 1%; p=0.11)</td></tr>
<tr><td>97X</td><td>Bayreuth Germany</td><td>-</td></tr>
<tr><td>03D</td><td>Royal Marsden London</td><td>No patient developed clinical cardiac failure. One patient in the standard 3-weekly AC arm had a fall in LVEF ≥10% to below 50%, which subsequently recovered to 53%. No significant difference in LVEF falls of ≥10% between the 3-weekly and 2-weekly arms was observed on completing treatment (p=0.7), one (p=0.8) and two (p=0.8) years post therapy.</td></tr>
<tr><td>03Q</td><td>GIM2 Italy</td><td>Not reported.</td></tr>
<tr><td>04)</td><td>CAMs Beijing</td><td>In the standard 3-weekly arm cardiac insufficiency occurred in one patient 3 weeks after the treatment had started and the patient refused to carry on the treatment. Cardiotoxicity was one of the main adverse events occurring, however there was no cardiotoxicity of grade 3 or above in either arm.</td></tr>
<tr><td>05P</td><td>TACT2 UK</td><td>Cardiotoxicity was not mentioned. One death due to infective endocarditis after distant disease recurrence in the standard 3-weekly epirubicin followed by CMF group.</td></tr>
<tr><td colspan="3">(A2) Shorter interval between cycles (but additional drugs in control arm)</td></tr>
<tr><td>93#</td><td>EORTC MA.10</td><td>Forty-six patients experienced a significant drop of the LVEF (≥15%), 27 (12%) and 19 (8.6%) in the 2-weekly and the 3-weekly EC arm, respectively. Four of these patients further developed symptomatic congestive heart failure (two patients in each arm). One toxic death in the 4-weekly FEC arm was due to congestive heart failure.</td></tr>
<tr><td>93W</td><td>Hamburg Germany</td><td>Not reported.</td></tr>
<tr><td>99Y</td><td>GEPARDUO Germany</td><td>Cardiac arrhythmia occurred in fewer than 5% of patients in each arm.</td></tr>
<tr><td>00Y</td><td>GASD Germany</td><td>Not reported.</td></tr>
<tr><td>07B</td><td>SBG 2004-1 PANTHER</td><td>Cardiotoxicity was not mentioned. There were no toxicity related deaths in the study. There was no pleural or pericardial adverse event.</td></tr>
<tr><td colspan="3">(A3) Shorter interval between cycles (plus additional treatment in dose-dense arm)</td></tr>
<tr><td>00F</td><td>MA.21 Canada</td><td>Incidence of grade 3 and 4 cardiac toxicity among incidence of worst ever toxicity:
<table>
<tr><th></th><th>4-weekly arm (FEC)</th><th>2-weekly arm (EC;P)</th><th>3-weekly arm (AC;P)</th><th>p value (Fisher's exact test)</th></tr>
<tr><td>Decreased LVEF (acute), grade 3/4</td><td>0.4% (3/680)</td><td>0.3% (2/688)</td><td>0.3% (2/674)</td><td>0.02</td></tr>
<tr><td>Decreased LVEF (delayed), grade 3/4</td><td>2.1% (14/680)</td><td>0.7% (5/688)</td><td>0.3% (2/674)</td><td><0.001</td></tr>
</table></td></tr>
<tr><td>02C</td><td>PREPARE/GermanBG 49</td><td>No treatment-related death or congestive heart failure occurred during therapy. Cardiovascular events were rare.</td></tr>
</table>

**(B1) Sequential vs concurrent anthracycline/taxane usage (same drugs in each arm)**		
$98D_{1+2}$	BIG 02-98	Grade 3–4 cardiac function toxicity was observed in 0% of both the sequential docetaxel arm and the concurrent docetaxel arm.
99G	NSABP B-30 USA	The percent of patients with grade 3 and 4 left ventricular dysfunction was 1% in both the sequential ACD arm and the doxorubicin-docetaxel arm, and <1% in the concurrent ACD arm (p=0.89 for comparing the three arms).
00H	BCIRG 005 USA	There were no statistical significant differences in the incidence of CHF between the comparison arms, in terms of either CHF in association with an absolute decrease of LVEF >15% from baseline and below LNL, CHF in association with an absolute decrease of 15% ≥LVEF >10% from baseline and below LNL, or CHF with signs/symptoms from a clinical standpoint, regardless of LVEF decline (<1% in both comparison arms).
01=	HORG CT/01.04 Greece	-
06&	Sanofi Aventis USA	Grade ≥3 clinical occurred in 1.3% (95% CI 0.0 to 6.9) in the sequential administration arm and 4.0% (95% CI 0.8 to 11.2) in the concurrent administration arm. Overall, 32.1% patients in the sequential administration arm and 28.0% patients in the concurrent administration arm experienced at least one cardiac failure event. No cardiac death in either arm.
06T	BOOG 2007-02 NL	No LVEF was done routinely in the study. No toxic deaths. The toxicity of both regimens was manageable.
**(B2) Sequential vs concurrent anthracycline/taxane usage (difference in drugs between arms)**		
00)	Pfizer NCT00140075	-
04F	GEICAM 2003-10 Spain	There was one treatment related death due to cardiorespiratory arrest in the sequential anthracycline and taxane arm. Three patients experienced cardiac events in the concurrent anthracycline and taxane arm while receiving capecitabine (two had angina and one had myocardial infarction).
**(C) Shorter interval between cycles AND sequential anthracycline/taxane usage**		
98Q	AGO-1 Germany	Grade 3 CHF was observed in one patient treated with intensive dose-dense chemotherapy (<1%) and in two patients treated with conventionally scheduled chemotherapy (=1%), p=0.372. There was no statistically significant difference between the two arms in terms overall CHF of all grades (p=0.675).
$98V_1$	AGO IDD-ETC Germany	No severe CHF was reported. No grade 3 CHF was observed in both study arms.
00X	HE10/00 Greece	There were no statistically significant differences in the incidence of severe cardiotoxicity: 0.2% with grade 3 and 0% with grade 4 cardiotoxicity in both arms.
03L	AERO B-03 France	The incidence of grade 3-4 cardiac toxicity was 3% and 0% respectively in the 2-weekly sequential arm (EC;D, or D;EC) and the 3-weekly concurrent DEC arm.
04=	SBG 2004-1 feasibility Sweden	There were no toxicity related deaths, pleural or pericardial adverse events. After a median follow-up of 10 years, there were no documented cases of clinically diagnosed cardiotoxicity.
04D	NSABP-B38 USA	One cardiac ischemia/infarction death during treatment was recorded in the 3-weekly concurrent AC and docetaxel with no deaths in the 2-weekly AC then paclitaxel arm.
**(D) Same total dose in fewer cycles**		
85A	CALGB 8541 USA	-
$88T_1$	NCC Tokyo	No cardiotoxicity was observed with either treatment arm, no toxic deaths occurred, and the side effects in the high dose, six-cycle group were manageable.
94L	SWOG 9313 USA	Incidence of grade 3 and 4 cardiac toxicity was <1 and 1, and grade 3 or 4 congestive heart failure was 0.4% and 1.1% in the current AC group and the A then C group, respectively. Cardiac toxicity was significantly worse in the sequential arm.

AC = doxorubicin and cyclophosphamide. ACD = doxorubicin, cyclophosphamide, and docetaxel. EC = 4-epirubicin and cyclophosphamide. CHF = congestive heart failure. CMF = cyclophosphamide, methotrexate, and fluorouracil. FEC = fluorouracil (5FU), epirubicin and cyclophosphamide. LVEF = left ventricular ejection fraction. LNL = lower normal limit.

P33: Compliance (as reported in study reports if patient level data not provided)

Category	Events/Women: Allocated Dose−intense	Events/Women: Allocated Standard	Dose−intense events: Logrank O−E	Dose−intense events: Variance of O−E	Ratio of annual event rates: Ratio (& CI)
**(A1) Shorter interval between cycles (same drugs, doses and number of cycles in each arm)**					
92E GONO MIG1 Italy	40/604 (6·6%)	38/610 (6·2%)	1·2	18·3	
92J Pisa/Genoa Italy	5/73 (6·8%)	3/77 (3·9%)	1·1	1·9	
97D CALGB 9741 USA			*(no data)*		
97X Bayreuth Germany			*(no data)*		
03D Royal Marsden London			*(no data)*		
03Q GIM 2 Italy	110/1002 (11·0%)	130/1001 (13·0%)	−10·1	52·8	0·83 (0·63 − 1·08)
04) CAMS Beijing	1/50 (2·0%)	2/51 (3·9%)	−0·5	0·7	
05P TACT2 UK	323/2170 (14·9%)	333/2221 (15·0%)	−1·2	140·0	0·99 (0·84 − 1·17)
**(A1) subtotal with data §**	**479/ 3899 (12·3%)**	**506/ 3960 (12·8%)**	**−9·5**	**213·7**	**0·96 (0·84 − 1·09)** 2p > 0·1; NS
**(A2) Shorter interval between cycles (but additional drugs in control arm)**					
93# EORTC 10921/MA.10	33/224 (14·7%)	41/224 (18·3%)	−4·0	15·5	0·77 (0·47 − 1·27)
93W Hamburg Germany			*(no data)*		
99Y GEPARDUO Germany	34/454 (7·5%)	105/456 (23·0%)	−35·3	29·5	0·30 (0·21 − 0·43)
00Y GASD Germany			*(no data)*		
07B SBG 2004−1 PANTHER	170/1006 (16·9%)	52/1008 (5·2%)	59·1	49·4	
**(A2) subtotal with data §**	**237/ 1684 (14·1%)**	**198/ 1688 (11·7%)**	**19·8**	**94·4**	**1·23 (1·01 − 1·51)** 2p = 0·04 adverse
**(A3) Shorter interval between cycles (plus additional treatment in dose−dense arm)**					
00F MA.21 Canada	119/701 (17·0%)	70/702 (10·0%)	24·6	40·9	
02C PREPARE/GermanBG 49	66/363 (18·2%)	59/370 (15·9%)	4·1	26·0	
**(A3) subtotal**	**185/ 1064 (17·4%)**	**129/ 1072 (12·0%)**	**28·7**	**66·9**	**1·54 (1·21 − 1·95)** 2p = 0·0004 adverse
**(B1) Sequential vs concurrent anthracycline/taxane (same drugs in each arm)**					
98D BIG 02−98	86/959 (9·0%)	57/960 (5·9%)	14·5	33·1	
99G NSABP B−30 USA	249/1783 (14·0%)	53/1784 (3·0%)	98·0	69·1	
00H BCIRG 005 USA	164/1649 (9·9%)	115/1649 (7·0%)	24·5	63·9	
01= HORG CT/01.04 Greece	11/329 (3·3%)	12/329 (3·6%)	−0·5	5·6	
03K Fudan CHN TAX 619			*(no data)*		
06& Sanofi−Aventis USA			*(no data)*		
06T BOOG 2007−02 NL	9/100 (9·0%)	3/102 (2·9%)	3·1	2·8	
**(B1) subtotal with data §**	**519/ 4820 (10·8%)**	**240/ 4824 (5·0%)**	**139·6**	**174·5**	**2·22 (1·92 − 2·58)** 2p < 0·00001 adverse
**(B2) Sequential vs concurrent anthracycline/taxane (but some difference in drugs between arms)**					
00) Pfizer			*(no data)*		
04F GEICAM 2003−10 Spain	33/669 (4·9%)	90/715 (12·6%)	−26·5	28·0	0·39 (0·27 − 0·56)
**(B2) subtotal with data §**	**33/ 669 (4·9%)**	**90/ 715 (12·6%)**	**−26·5**	**28·0**	**0·39 (0·27 − 0·56)** 2p < 0·00001
**(C) Shorter interval between cycles AND sequential anthracycline/taxane**					
98Q AGO−1 Germany			*(no data)*		
98V1 AGO IDD−ETC Germany	102/643 (15·9%)	55/612 (9·0%)	21·6	34·3	
00X HE 10/00 Greece	35/564 (6·2%)	14/557 (2·5%)	10·3	11·7	
03L AERO B−03 France	8/65 (12·3%)	2/35 (5·7%)	1·5	2·1	
04= SBG 2004−1 Sweden	10/84 (11·9%)	4/40 (10·0%)	0·5	2·7	
04D NSABP B−38 USA	196/1634 (12·0%)	146/1630 (9·0%)	24·8	76·6	
**(C) subtotal with data §**	**351/ 2990 (11·7%)**	**221/ 2874 (7·7%)**	**58·7**	**127·4**	**1·59 (1·33 − 1·89)** 2p < 0·00001 adverse
**(D) Same total dose in fewer cycles**					
85A CALGB 8541 USA	17/528 (3·2%)	7/522 (1·3%)	4·9	5·9	
88T1 NCC Tokyo	3/101 (3·0%)	8/101 (7·9%)	−2·5	2·6	0·38 (0·11 − 1·29)
94L SWOG 9313 USA	244/1564 (15·6%)	191/1612 (11·8%)	29·8	93·9	
**(D) subtotal**	**264/ 2193 (12·0%)**	**206/ 2235 (9·2%)**	**32·2**	**102·4**	**1·37 (1·13 − 1·66)** 2p = 0·001 adverse
**Total known**	**2068/ 17319 (11·9%)**	**1590/ 17368 (9·2%)**	**243·0**	**807·4**	**1·351 (1·261 − 1·448)** 2p < 0·00001 adverse
**Total unknown §**	**−/0**	**−/0**			

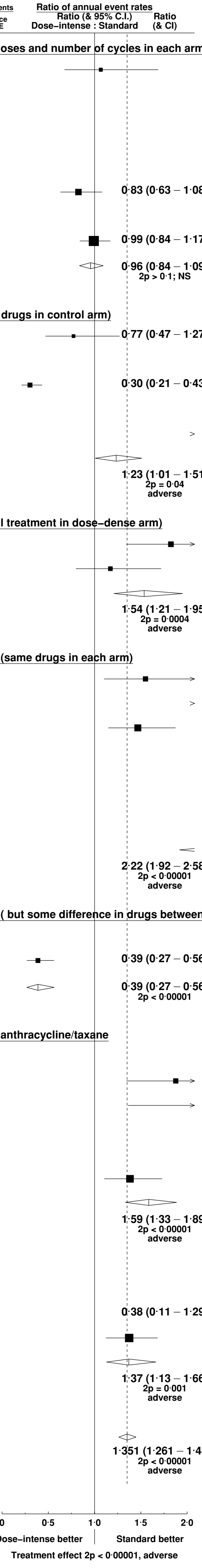

Heterogeneity between 7 subtotals: $\chi^2_6 = 117{\cdot}6$; $p < 0{\cdot}00001$

Heterogeneity within subtotals: $\chi^2_{17} = 172{\cdot}7$; $p < 0{\cdot}00001$

Heterogeneity between 24 categories: $\chi^2_{23} = 290{\cdot}3$; $p < 0{\cdot}00001$

§ 0 trials with no data do not contribute to subtotals or to the overall total.

P34 - 43: Statistical analysis plan

Statistical Analysis Plan for EBCTCG meta-analysis of dose intense trials
January 2017, Version 1

Cytokinetic modelling suggests that increasing the dose intensity of cytotoxic therapy by shortening the intervals between courses, or by using sequential rather than concurrent treatment schedules may enhance efficacy. This EBCTCG meta-analysis aims to bring together the evidence from relevant trials to clarify the balance of risks and benefits of dose intensification of anthracycline and taxane chemotherapy.

<u>Cohort definitions</u>

Cohort 1 – Dose-dense trials (comparing shorter vs longer interval between courses)
Trials comparing dose-dense chemotherapy versus standard chemotherapy regimens subdivided by:

a) Unconfounded trials (ie trials with same agents at same doses)
b) Confounded trials (trials with additional agents or higher doses in the control group)
c) Confounded trials (trials with additional agents or higher doses in the dose-dense group)

Cohort 2 – Scheduling trials (comparing sequential vs concurrent taxane/ anthracycline chemotherapy)

a) Unconfounded trials (ie trials with same agents in both groups)
b) Confounded trials (trials with additional agents or higher doses in one of the treatment groups)

Cohort 3 – Dose-dense scheduling trials (comparing shorter vs longer interval between courses and sequential vs concurrent administration)

Cohort 4 – Trials comparing higher vs lower dose
Trials of dose escalation, which compares higher versus lower doses of anthracyclines, taxanes and cyclophosphamide maintaining the same interval between courses subdivided by:

a) Increase in dose of anthracycline (cumulative dose difference <100mg Adriamycin or 150mg epirubicin)
b) Increase in dose of anthracycline (cumulative dose difference ≥100mg Adriamycin or 150mg epirubicin)
c) Increase in dose of anthracycline and of other drugs
d) Increase in dose of cyclophosphamide

Data will be sought from all relevant randomised trials, irrespective of size or primary outcome measure. Full details of search strategies, study selection and data checking procedures are provided in the EBCTCG's PRISMA-IPD statement, available at https://www.ctsu.ox.ac.uk/files/research/prisma-ipd-statement-for-ebctcg.pdf
Cohort 4 will be analysed and reported separately.

Primary outcomes

The main endpoint definitions and methods are those used in previous EBCTCG reports, but with some amendments that reflect the potential impact of dose intense chemotherapy.

Breast cancer recurrence: includes distant recurrence, local recurrence and new second primary breast cancer (ipsilateral or contralateral), and the definition of these will be as in each trial.

Breast cancer mortality: Information about mortality rates without recurrence will be subtracted from information about overall mortality rates (as in previous EBCTCG reports). The same statistical methods will be used to construct Kaplan-Meier graphs that estimate breast cancer mortality (i.e. the pattern of mortality that would have been seen if it had been possible to avoid all deaths before or after recurrence from causes other than breast cancer).

Death without recurrence: i.e. without the secretariat having record of recurrence.

All-cause mortality

Exploratory endpoints

First distant recurrence: includes distant recurrence and ignores any prior loco-regional recurrence or contralateral breast cancer.

Loco-regional recurrence as first event: includes ipsilateral breast, chest wall and loco-regional lymph nodes (axilla and SCF).

Time to contralateral new primary breast cancer as first event.

Second primary cancer at sites other than breast

Non-fatal cardiovascular events: The incidence of non-fatal cardiovascular events (e.g. MI, stroke, congestive heart failure) requiring hospitalisation will be compared.

Data checking

The usual EBCTCG quality assurance checks for range, consistency and balance between randomisation arms will be undertaken prior to analysis. In addition, to validate the death without distant recurrence analyses (see below), the quality of second recurrence and cause of death information for each trial will be explored and, if appropriate for particular trials, death without distant recurrence will be estimated as overall mortality rates minus mortality rates without any recurrence rather than without distant recurrence. The dependence of deaths without recorded recurrence on TN status will also be investigated with any association used to estimate what

proportion might actually have been from breast cancer. Checks will be undertaken to compare the incidence of death without recurrence and of second cancers (overall and by site) by age group in each trial to characterise whether these might include miscoded breast cancer events.

<u>Analyses</u>
Primary analyses will be by Intention-to-Treat (ITT), including all randomised patients irrespective of treatment compliance.

<u>Subgroup analyses of recurrence</u>
Explanatory subgroup analyses will be undertaken but, given the well-known hazards of subgroup analysis, will be interpreted appropriately cautiously. Investigation of potential interactions between tumour or patient characteristics and treatment efficacy will be undertaken with breast cancer recurrence as primary outcome. Unless the treatment efficacy is shown by the above analyses to vary by dose dense schedule, dose escalation or scheduling, the analyses below will include all trials in cohorts 1, 2, 3 and 4.

Subgroup analyses of recurrence Forest plots for subgroup analyses by:

- Site of recurrence (distant metastasis, local recurrence or contralateral breast cancer)
- Age (<45, 45-54, 55-69, ≥70, unknown)
- ER status (1=ER-poor, 2=ER+, ER unknown)
- ER/PR status (1=ER+/PR-poor, 2=ER+PR+)
- Nodal status (negative, N1-3, N4+, N unknown)
- T-stage (T1, T2, T3/T4, T stage unknown);
- Histological grade (1, 2, 3, unknown)
- Tumour histology (ductal, lobular, other, unknown)
- HER2 status (1=HER2-negative, 2=HER2-positive, 3=unknown)
- Proliferation index (%Ki-67 0-9, 10-19, 20-49, 50+)
- Recurrence rates in years 0-1, 2-4, 5-9, and 10+ after randomisation will be reported

Where adequate numbers exist, Kaplan-Meier curves for recurrence (to year 10, with ancillary tabulations of the outcome by allocated treatment in each separate follow-up period (0-1, 2-4, 5-0, 10+ yrs.) will be undertaken.

A current list of relevant trials and publications can be found in appendix 1.

The data variables requested is detailed in appendix 2.

Appendix 1. Dose intense trial list

Outline of dose-intensity trials

YEAR CODE	TRIAL NAME	COMPARISON	DOSE-INTENSITY RATIO	SIZE	DATA
**(A1) Shorter interval between cycles (same drugs, doses and number of cycles in each arm)**					
92E	GONO MIG1 Italy	6**F**$_{600}$**E**$_{60}$**C**$_{600}$ **[q2†v q3]**	E 1.5	1214	✓
92J	Pisa/Genoa Italy	[3**FEC**‡; 3(**F**$_{600}$**E**$_{60}$**C**$_{600}$; **CMF**)] **[q2†v q3]**	E 1.5	150	✓
97D	CALGB 9741 USA	[(4**A**$_{60}$;4**P**$_{175}$;4**C**$_{600}$) or (4**AC**;4**P**$_{175}$)] **[q2†v q3]**	A 1.5 P 1.5	2005	✓
97X	Bayreuth Germany	3**E**$_{120}$**C**$_{600}$‡ **[q2†v q3]**	E 1.5	140	✓
03D	Royal Marsden London	(4**AC**/4**EC**)‡ **[q2†v q3]**	A 1.5 E 1.5	128	×
03Q$_{1-4}$	GIM 2 Italy	[4(**E**$_{90}$**C**$_{600}$ ± **F**$_{600}$);4**P**$_{175}$] **[q2†v q3]**	E 1.5 / P 1.5	2003	✓
04)	CAMS Beijing	6**E**$_{120}$**P**$_{175}$ **[q2†v q3]**	E 1.5 P 1.5	101	✓
05P	TACT2 UK	4**E**$_{100}$**[q2†v q3]**;4**CMF q4**/4**Cap**$_{2500x14}$ **q3**	E 1.5	4391	✓
**(A2) Shorter interval between cycles (but additional drugs in control arm)**					
93#	EORTC 10921/ MA.10	(6**E**$_{120}$**C**$_{830}$**q2**† v 6**F**$_{500x2}$**E**$_{60x2}$**C**$_{75x14}$**q4)** ‡	E 2.0	448	✓
93W	Hamburg Germany	4**E**$_{120}$**C**$_{600}$**q2**† v 4**E**$_{90}$**C**$_{600}$**q3**;3**CMFq4**	E 2.0	183	×
99Y	GEPARDUO Germany	[4**A**$_{50}$**D**$_{75}$**q2**† v (4**AC**; 4**D**$_{100}$)**q3**]‡	A 1.3 D 1.1	910	✓
00Y	GASG Germany	(4**E**$_{120}$†; 4**P**$_{175}$)**q2** v (4**EC**; 4**P**$_{175}$)**q3**	E 2.0 P 1.5	884	×
07B	SBG 2004-1 PANTHER	(4**E**$_{100}$**C**$_{825}$; 4**D**$_{80}$) **q2**† v (3**F**$_{500}$**E**$_{100}$**C**$_{500}$;3**D**$_{100}$) **q3**†	E 1.5 D 1.2	2014	✓
**(A3) Shorter interval between cycles (plus additional treatment in dose-dense arm)**					
00F	MA.21 Canada	(6**E**$_{120}$**C**$_{830}$**q2**† v 4**ACq3**);4**P**$_{175}$**q3**	E/A 2.0	1403	✓
02C	PREPARE/GermanBG 49	[(3**E**$_{150}$;3**P**$_{225}$)**q2**†; 3**CMF q4** v (4**EC**;4**P**$_{175}$)**q3**†]‡	E 2.5 P 1.9	733	✓
**(B1) Sequential vs concurrent anthracycline/taxane usage (same drugs in each arm)**					
98D$_{1+2}$	BIG 02-98	(3**A**$_{75}$; 3**D**$_{100}$ v 4**A**$_{50}$**D**$_{75}$)**q3**; 3**CMF q4**	A 1.5 D 1.3	1919	(✓)
99G	NSABP B-30 USA	(4**AC**; 4**D**$_{100}$ v 4**ADC**†) **q3**	A 1.2 D 1.3	3567	✓
00H	BCIRG 005 USA	(4**AC**; 4**D**$_{100}$ v 6**ADC**) **q3**	A 1.2 D 1.3	3298	✓
01=	HORG CT/01.04 Greece	(4**E**$_{90}$; 4**D**$_{75}$**q3** v 6**E**$_{75}$**D**$_{75}$†) **q3**	E 1.2 D 1.0	658	✓
03K	Fudan CHN TAX 619	(4**D**$_{100}$;4**A**$_{60}$/**E**$_{75}$**C**$_{600}$ v 6**D**$_{75}$**A**$_{50}$/**E**$_{60}$**C**$_{500}$) **q3**	E1.3 D 1.3	603	×
06&	Sanofi-Aventis USA	(4**AC**; 4**D**$_{100}$ v 6**ADC**) † + **Bev**$_{15x34}$ **q3**	A 1.2 D 1.3	155	×
06T	BOOG 2007-02 NL	(4**AC**; 4**D**$_{100}$ v 6**ACD**†) **q3**‡	A 1.2 D 1.3	202	✓
**(B2) Sequential vs concurrent anthracycline/taxane usage (difference in drugs between arms)**					
00)	Pfizer NCT00140075	[4**EC**; 4(**P**$_{175}$/**D**$_{75}$) v 8**E**75(**P**175/**D**$_{75}$)] **q3**	E 1.2 P/D 1.0	617	×
04F	GEICAM 2003-10 Spain	(4**EC**; 4**D**$_{100}$†) **q3** v (4**E**$_{90}$**D**$_{75}$†; 4**Cap**$_{2500x14}$) **q3**	E 1.0 D 1.3	1384	✓
**(C) Shorter interval between cycles AND sequential anthracycline/taxane usage**					
98Q	AGO-1 Germany	[(3**E**$_{150}$; 3**P**$_{250}$)**q2**† v 4**E**$_{90}$**P**$_{175}$**q3**]‡; 3**CMF q4**	E 2.5 P 2.1	668	✓

$98V_1$	AGO IDD-ETC Germany	(3$\mathbf{E}_{150}$; 3$\mathbf{P}_{225}$; 3$\mathbf{C}_{2500}$) **q2**† v (4**EC**; 4$\mathbf{P}_{175}$) **q3**	E 2.5 P 1.9	1255	✓
00X	HE 10/00 Greece	[(3$\mathbf{E}_{110}$; 3$\mathbf{P}_{250}$) **q2**† v 4$\mathbf{E}_{83}\mathbf{P}_{187}$ **q3**]; 3**CMF** **q2**†	E 2.0 P 2.0	1121	✓
03L	AERO B-03 France	(4**EC**; 4**D** or 4$\mathbf{D}_{100}$; 4$\mathbf{E}_{100}\mathbf{C}_{600}$) **q2**† v 6$\mathbf{E}_{75}\mathbf{D}_{75}\mathbf{C}_{500}$ **q3**	E 2.0 D 2.0	100	✓
04=	SBG 2004-1 Sweden	[(4**EC**; 4**D**) or (4$\mathbf{E}_{100}\mathbf{C}_{825}$; 4$\mathbf{D}_{80}$)] **q2**† v 6**ADC** **q3**†	E/A1.9 D 1.5	124	✓
04D	NSABP B-38 USA	(4**AC**; 4$\mathbf{P}_{175}$) **q2**† v 6**ADC** **q3**†	A 1.8 P/D ~1.8	3264	✓
**(D) Same total dose in fewer cycles**					
85A	CALGB 8541 USA	(4$\mathbf{F}_{600x2}\mathbf{A}_{60}\mathbf{C}_{600}$ v 6$\mathbf{F}_{400x2}\mathbf{A}_{40}\mathbf{C}_{400}$) **q4**	A 1.5	1050	✓
$88T_1$	NCC Tokyo	(6$\mathbf{C}_{130}\mathbf{M}_{26}\mathbf{F}_{600}\mathbf{A}_{26}$ v 12$\mathbf{C}_{65}\mathbf{M}_{13}\mathbf{F}_{300}\mathbf{A}_{13}$) **q4**	A 2.0	202	✗
94L	SWOG 9313 USA	[(4$\mathbf{A}_{40x2}$ **q3**; 3$\mathbf{C}_{2400}$ **q2**) v 6$\mathbf{A}_{54}\mathbf{C}_{1200}$**q3**] †	A 1.5	3176	✓

† with growth factor; ‡ neoadjuvant treatment; (✓) data last received before 2005

Dose intense trial list

YEAR CODE	TRIAL NAME	REFERENCES
**(A1) Shorter interval between cycles (same drugs, doses and number of cycles in each arm)**		
92E	GONO Italy	Venturini M, Del Mastro L, Aitini E, et al. Dose-dense adjuvant chemotherapy in early breast cancer patients: results from a randomized trial. *J Natl Cancer Inst* 2005; **97**: 1724-33.
92J	PisaGenoa	Baldini E, Gardin G, Giannessi PG, et al. Accelerated versus standard cyclophosphamide, epirubicin and 5-fluorouracil or cyclophosphamide, methotrexate and 5-fluorouracil: a randomized phase III trial in locally advanced breast cancer. *Ann Oncol* 2003; **14**(2): 227-32.
97D	CALGB 9741	Citron ML, Berry DA, Cirrincione C, et al. Randomized trial of dose-dense versus conventionally scheduled and sequential versus concurrent combination chemotherapy as postoperative adjuvant treatment of node-positive primary breast cancer: first report of Intergroup Trial C9741/Cancer and Leukemia Group B Trial 9741. *J Clin Oncol* 2003; **21**(8): 1431-9.
97X	Bayreuth	Wulfing P, Tio J, Kersting C, et al. Expression of Endothelin-A-Receptor predicts unfavourable response to neoadjuvant chemotherapy in locally advanced breast cancer. *Br J Cancer* 2004; **91**(3): 434-40.
03D	RM NHST London	Jones RL, Walsh G, Ashley S, et al. A randomised pilot Phase II study of doxorubicin and cyclophosphamide (AC) or epirubicin and cyclophosphamide (EC) given 2 weekly with pegfilgrastim (accelerated) vs 3 weekly (standard) for women with early breast cancer. *Br J Cancer* 2009; **100**(2): 305-10.
$03Q_{1-4}$	GIM2 Italy	Del Mastro L, De Placido S, Bruzzi P, et al. Fluorouracil and dose-dense chemotherapy in adjuvant treatment of patients with early-stage breast cancer: an open-label, 2 x 2 factorial, randomised phase 3 trial. *Lancet* 2015; **385**: 1863-72.
04)	CAMs Beijing	Wu WH, Li Q, Xu BH, et al. [Safety of adjuvant dose-dense chemotherapy with paclitaxel and epirubicin for high-risk breast cancer] [Chinese]. *Chung Hua Chung Liu Tsa Chih* 2008; **30**(7): 548-51.
05P	TACT2	Cameron D, Morden JP, Canney P, et al. Accelerated versus standard epirubicin followed by cyclophosphamide, methotrexate, and fluorouracil or capecitabine as adjuvant therapy for breast cancer in the randomised UK TACT2 trial (CRUK/05/19): a multicentre, phase 3, open-label, randomised, controlled trial. *Lancet Oncol* 2017: **18**(7):929-945.
**(A2) Shorter interval between cycles (but additional drugs in control arm)**		
93#	EORTC MA.10	Therasse P, Mauriac L, Welnicka Jaskiewicz M, et al. Final results of a randomized phase III trial comparing cyclophosphamide, epirubicin, and fluorouracil with a dose-intensified epirubicin and cyclophosphamide + filgrastim as neoadjuvant treatment in locally advanced breast cancer: an EORTC-NCIC-SAKK 5ulticentre study. *J Clin Oncol* 2003; **21**(5): 843-50.
93W	Hamburg	Untch M, Thomssen C, Steffen K, et al. Five year results of a randomised 5ulticentre dose intense (DI-EC) study with Epirubicin © and

		Cyclophosphamide © in high risk breast cancer patients-a treatment of short duration with comparable efficacy to conventional chemotherapy. *Breast Cancer Res Treat* 2002; **76**(Suppl 1): S158, A641.
99Y	GEPARDUO	von Minckwitz G, Raab G, Caputo A, et al. Doxorubicin with cyclophosphamide followed by docetaxel every 21 days compared with doxorubicin and docetaxel every 14 days as preoperative treatment in operable breast cancer: the GEPARDUO study of the German Breast Group. *J Clin Oncol* 2005; **23**(12): 2676-85.
00Y	GASD Germany	Eggemann H, Krocker J, Kuemmel S, et al. Sequential dose-dense epirubicin/paclitaxel (E-T) with G-CSF support compared to standard EC — T (epirubicin/cyclophosphamide followed by paclitaxel) for patients with operable breast cancer and 1-3 positive lymph nodes first toxicity analysis. *Breast Cancer Res Treat* 2002; **76**(Suppl 1): S159, A646.
07B	SBG 2004-1 Panther	Foukakis T, von Minckwitz G, Bengtsson N-O, et al. Effect of tailored dose-dense chemotherapy vs standard 3-weekly adjuvant chemotherapy on recurrence-free survival among women with high-risk early breast cancer. A randomized trial. *JAMA* 2016; **316**(18): 1888-96.
**(A3) Shorter interval between cycles (plus additional treatment in dose-dense arm)**		
00F	MA21	Burnell M, Levine MN, Chapman J-AW, et al. Cyclophosphamide, epirubicin, and fluorouracil versus dose-dense epirubicin and cyclophosphamide followed by paclitaxel versus doxorubicin and cyclophosphamide followed by paclitaxel in node-positive or high-risk node-negative breast cancer. *J Clin Oncol* 2010; **28**(1): 77-82.
02C	PREPARE	Untch M, Fasching PA, Konecny GE, et al. PREPARE trial: a randomized phase III trial comparing preoperative, dose-dense, dose-intensified chemotherapy with epirubicin, paclitaxel and CMF versus a standard-dosed epirubicin/cyclophosphamide followed by paclitaxel +/- darbepoetin alfa in primary breast cancer—results at the time of surgery. *Ann Oncol* 2011; **22**(9): 1988-98.
**(B1) Sequential vs concurrent anthracycline/taxane usage (same drugs in each arm)**		
98D$_{1+2}$	BIG 02-98	Francis P, Crown J, Di Leo A, et al. Adjuvant chemotherapy with sequential or concurrent anthracycline and docetaxel: Breast International Group 02-98 randomized trial. *J Natl Cancer Inst* 2008; **100**: 121-33.<br>Oakman C, Francis PA, Crown J, et al. Overall survival benefit for sequential doxorubicin-docetaxel compared with concurrent doxorubicin and docetaxel in node-positive breast cancer—8-year results of the Breast International Group 02-98 phase III trial. *Ann Oncol*. 2013;**24**(5):1203-11.
99G	NSABP B-30	Swain SM, Jeong JH, Geyer CEJ, et al. Longer therapy, iatrogenic amenorrhea, and survival in early breast cancer. *N Engl J Med* 2010; **362**(22): 2053-65.
00H	BCIRG 005	Mackey JR, Pienkowski T, Crown J, et al. Long-term outcomes after adjuvant treatment of sequential versus combination docetaxel with doxorubicin and cyclophosphamide in node-positive breast cancer: BCIRG-005 randomized trial. *Ann Oncol* 2016; **27**(6): 1041-7.<br>Eiermann W, Pienkowski T, Crown J, et al. Phase III study of doxorubicin/cyclophosphamide with concomitant versus sequential docetaxel as adjuvant treatment in patients with human epidermal growth factor receptor 2-normal, node-positive breast cancer: BCIRG-005 trial. *Journal of Clinical Oncology* 2011; **29**(29): 3877-84.
01=	CT/01.04	-
03K	Fudan CHN TAX 619	Shen ZZ, Liu GY, Shao ZM, et al. A feasibility study of docetaxel in combination with anthracycline and cyclophosphamide or docetaxel followed by anthracycline plus cyclophosphamide in adjuvant therapy for operable breast cancer with positive axillary lymph nodes: CHN TAX 619. *Breast Cancer Research and Treatment* 2005; **94**(suppl 1): S107, A2066.<br>https://www.clinicaltrials.gov/ct2/show/NCT00525642?term=CHN+TAX+619&rank=1
06&	Sanofi Aventis	Yardley DA, Hart L, Waterhouse D, et al. Addition of bevacizumab to three docetaxel regimens as adjuvant therapy for early stage breast cancer. *Breast Cancer Research & Treatment* 2013; **142**(3): 655-65.
06T	BOOG 2007-02	Vriens BE, Aarts MJ, de Vries B, et al. Doxorubicin/cyclophosphamide with concurrent versus sequential docetaxel as neoadjuvant treatment in patients with breast cancer. *Eur J Cancer* 2013; **49**(15): 3102-10.
**(B2) Sequential vs concurrent anthracycline/taxane usage (difference in drugs between arms)**		

00)	Pfizer NCT00140075	Lambert-Falls R, Deutsch MA, Desch C, Zhou K, Perez E. Phase III adjuvant trial of concurrent epirubicin/taxane vs. sequential epirubicin/cyclophosphamide followed by taxane for node positive breast cancer. *J Clin Oncol* 2006; **24**(18S): 21s, A573.
04F	GEICAM 2003-10	Martín M, Ruiz Simón A, Ruiz Borrego M, et al. Epirubicin plus cyclophosphamide followed by docetaxel versus epirubicin plus docetaxel followed by capecitabine as adjuvant therapy for node-positive early breast cancer: results from the GEICAM/2003-10 study. *J Clin Oncol* 2015; **33**(32): 3788-95.<br>Margolin S, Bengtsson NO, Carlsson L, et al. A randomised feasibility/phase II study (SBG 2004-1) with dose-dense/tailored epirubicin, cyclophoshamide (EC) followed by docetaxel (T) or fixed dosed dose-dense EC/T versus T, doxorubicin and C (TAC) in node-positive breast cancer. *Acta Oncol* 2011; **50**(1): 35-41.
**(C) Shorter interval between cycles ANF sequential anthracycline/taxane usage**		
98Q	AGO Germany	Untch M, Mobus V, Kuhn W, et al. Intensive dose-dense compared with conventionally scheduled preoperative chemotherapy for high-risk primary breast cancer. *J Clin Oncol* 2009; **27**(18): 2938-45.<br>Hudis CA. Intensive dose-dense compared with conventionally scheduled preoperative chemotherapy for high-risk primary breast cancer. *Breast Diseases* 2010; **21**(4): 377-8.
$98V_1$	ETC Germany	Moebus V, Jackisch C, Lueck HJ, et al. Intense dose-dense sequential chemotherapy with epirubicin, paclitaxel, and cyclophosphamide compared with conventionally scheduled chemotherapy in high-risk primary breast cancer: mature results of an AGO phase III study. *J Clin Oncol* 2010; **28**(17): 2874-80.
00X	HE10/00	Fountzilas G, Dafni U, Gogas H, et al. Postoperative dose-dense sequential chemotherapy with epirubicin, paclitaxel and CMF in patients with high-risk breast cancer: safety analysis of the Hellenic Cooperative Oncology Group randomized phase III trial HE 10/00. *Ann Oncol* 2008; **19**(5): 853-60.<br>Gogas H, Dafni U, Karina M, et al. Postoperative dose-dense sequential versus concomitant administration of epirubicin and paclitaxel in patients with node-positive breast cancer: 5-year results of the Hellenic Cooperative Oncology Group HE 10/00 phase III Trial. *Breast Cancer Research & Treatment* 2012; **132**(2): 609-19.
03L	AERO B-03	Piedbois P, Serin D, Priou F, et al. Dose-dense adjuvant chemotherapy in node-positive breast cancer: docetaxel followed by epirubicin/cyclophosphamide (T/EC), or the reverse sequence (EC/T), every 2 weeks, versus docetaxel, epirubicin and cyclophosphamide (TEC) every 3 weeks. AERO B03 randomized phase II study. *Ann Oncol* 2007; **18**(1): 52-7.
04=	SBG 2004-1 feasibility	Margolin S, Bengtsson NO, Carlsson L, et al. A randomised feasibility/phase II study (SBG 2004-1) with dose-dense/tailored epirubicin, cyclophoshamide (EC) followed by docetaxel (T) or fixed dosed dose-dense EC/T versus T, doxorubicin and C (TAC) in node-positive breast cancer. *Acta Oncol* 2011; **50**(1): 35-41.<br>Matikas A, Margolin S, Hellstrom M, et al. Long-term safety and survival outcomes from the Scandinavian Breast Group 2004-1 randomized phase II trial of tailored dose-dense adjuvant chemotherapy for early breast cancer. *Breast Cancer Res Treat* 2018; **168**(2): 349-55.
04D	NSABP-B38	Swain SM, Tang G, Geyer CE, Jr., et al. Definitive results of a phase III adjuvant trial comparing three chemotherapy regimens in women with operable, node-positive breast cancer: the NSABP B-38 trial. *Journal of Clinical Oncology* 2013; **31**(26): 3197-204.
**(D) Same total dose in fewer cycles**		
85A	CALGB CLB-8541	Budman DR, Berry DA, Cirrincione CT, et al. Dose and dose intensity as determinants of outcome in the adjuvant treatment of breast cancer. *J Natl Cancer Inst* 1998; **90**(16): 1205-11.
$88T_1$	NCC Tokyo	Fukutomi T, Akashi S, Nanasawa T, Yamamoto H. Adjuvant six cycles of high-dose adriamycin, cyclophosphamide, methotrexate, 5-fluorouracil (ACMF) vs. 12 cycles of low-dose ACMF with tamoxifen for premenopausal, node-positive breast cancer patients: results of a prospective randomized study. *J Surg Oncol* 1995; **60**(4): 242-6.
94L	SWOG 9313	Linden HM, Haskell CM, Green SJ, et al. Sequenced compared with simultaneous anthracycline and cyclophosphamide in high-risk stage I and II breast cancer: final analysis from INT-0137 (S9313). *Journal of clinical oncology : official journal of the American Society of Clinical Oncology* 2007; **25**(6): 656-61.

Appendix 2 –List of variables requested

CORE VARIABLES - BASELINE (Q1-27)

A) RANDOMISATION AND PATIENT CHARACTERISTICS (Q1-9)

1. **Your patient identifier** *(preferably specifying uniquely which trial as well as which patient)*
2. **Date of randomisation** *(specify your format for dates [in your covering document])*
3. **Allocated treatment** *(specify your codes)*
4. **Age** at randomisation *(years)* **NB Here & everywhere else, leaving an item Blank means Not Known**
5. **Height** at randomisation *(m)*
6. **Weight** at randomisation *(kg)*
7. **Menopausal status** at randomisation *(1=pre-, 2=peri-, 3=postmenopausal with intact ovaries & uterus, 4=ovarian ablation, 5=hysterectomy, 6=both [ie, 4 and 5])*
8. **Did chemotherapy cause apparently permanent cessation of menses?** *(1=no/not applicable, 2=yes)*
9. **Would your group's preferred analyses exclude this patient?** NB A few trial patients may be randomised in error, otherwise ineligible, lost with no follow-up, unevaluable or withdraw consent. *(1=no known reason for exclusion, 2=yes [specify main reason(s) for preferring exclusion, if known])*

B) SURGICAL DETAILS (Q10-11; OR, DEFINE AND USE YOUR OWN CODES)

10. **Breast surgery** *(1=none, 2=only lumpectomy or wide local excision, 3=quadrantectomy or sector resection, 4=partial mastectomy, 5=simple or total mastectomy, 6=radical mastectomy)*
11. **Axillary surgery** *(1=none, 2=sentinel node biopsy only, 3=axillary sampling, 4=surgical clearance of less than levels I & II, 5=full clearance of axillary levels I & II, 6=clearance of more than levels I & II, 7=axillary clearance, but levels cleared unspecified)*

C) NODAL STATUS (Q12-13; OR, USE YOUR OWN CODES [EG, TNM])

12. **Sentinel node biopsy** *(1=not done, 2=done and negative for cancer, 3= isolated tumour cells [≤0.2 mm], 4=micrometastasis, 5=macroscopic nodal deposit [>2 mm], 6=positive, size unknown)*
13. **Axillary status** *(specify codes, or: 1=N- histologically, 2=N- other/unknown method, 3=1-3 positive nodes, 4=4-9 [or 4+] positive, 5=10+ positive, 6=N+ histologically, unknown number, 7=N+ other/unknown method)*

D) TUMOUR CHARACTERISTICS (Q14-18; OR, USE YOUR OWN CODES [EG, TNM])

14. **Method first detected** *(1=mammographic screening, 2=incidental, 3=symptomatic, 4=other)*
15. **Laterality** *(1=left, 2=right, 3=bilateral)*
16. **Pathological grade** *(1=well differentiated, 2=moderately,3=poorly)*
17. **Histological type** *(1=invasive, not otherwise specified, 2=ductal, 3=lobular, 4=other, 5=mixed)*
18. **Tumour diameter in mm:** largest diameter of excised primary *(mm)*

E) RECEPTOR STATUS (Q19-24; OR, USE YOUR OWN CODES)

19. **Summary of Estrogen Receptor (ER) status of primary tumour** *(1=ER-poor, 2=ER+, 3=ER++ [define in cover document, unless ER-poor is <10 fm/mg and ER++ is ER definitely ≥100 fm/mg])*
20. **Quantitative ER measurement** *(measured in central/reference lab if possible, otherwise best available)*
21. **Units for ER** *(1=fm/mg, 2=% +ve by IHC, 3=Allred score [category score], 4=H-score, 9=other [specify])*

22. **Summary of Progesterone Receptor (PR) status of primary tumour** *(1=PR-poor, 2=PR+, 3=PR++ [define in cover document, unless PR-poor is <10 fm/mg and PR++ is PR definitely ≥100 fm/mg])*
23. **Quantitative PR measurement** *(done in central/reference lab if possible, otherwise best available)*
24. **Units for PR** *(coded as Q21)*

25. **Summary of HER2 status of primary** *(1=negative/normal, 2=positive/over-expressing)*
26. **Quantitative HER2 measurement** *(done in central/reference lab if possible, otherwise best available)*
27. **Units for HER2** *(1=IHC [% staining], 2=IHC score [0, 1+, 2+, 3+], 3=FISH [# copies], 4=FISH [HER2:CEP17 ratio], 5=CISH [# copies], 6=CISH [HER2:CEP17], 9=other [please specify])*

CORE VARIABLES – FOLLOW-UP (Q28-43)

F) NON-COMPLIANCE BEFORE ANY RECURRENCE (Q28-29; OR, USE YOUR OWN CODES)

28. **Any substantial deviation from trial treatment allocation (before any breast cancer recurrence)?**
    (1=no, 2=never started, 3=discontinued, 4=switched to opposite trial group, 5=other [specify])
29. **Date of first such deviation from allocated treatment** *(ignore deviations after recurrence)*

G) CANCER RECURRENCE AND SECOND CANCERS (Q30-40; OR, USE YOUR OWN CODES)

30. **Any recurrence of invasive breast cancer (ie, locoregional, contralateral or distant)?**
    NB Includes any occurrence of new ipsilateral or contralateral breast cancer *(1=no, 2=yes)*
31. **<u>If no:</u> Date patient last known to be free of such recurrence; <u>If yes:</u> Date of first such recurrence**

32. **Site of first distant recurrence (ie, possibly distant; not just locoregional/contralateral)**
    (1=no distant recurrence, 2=recurrence, unknown if distant, 3=distant recurrence, unknown/multiple sites, 4=only in distant soft tissue, 5=only in distant nodes, 6=only in bone, 7=only visceral, 8=only in CNS)
33. **Date of first distant recurrence** NB Locoregional recurrence can precede first distant recurrence

34. **Site of first locoregional recurrence** *(1=no locoregional recurrence recorded, 2=multiple or unspecified locoregional sites 3=only in breast [new or recurrent cancer] or chest wall, 4=only in axilla, 5=only in other locoregional nodes [eg, supraclavicular or internal mammary])*
35. **Date of first locoregional recurrence**

36. **Contralateral breast cancer?** *(1=no, 2=yes: new invasive cancer thought to have arisen during follow-up in the contralateral breast)*
37. **Date of first contralateral breast cancer**

NB If patient had more than one second malignancy during follow-up, <u>repeat</u> variables 38-40 for each.

38. **Site of any second malignancy *[except breast cancer]* during follow-up** *(Describe ALL sites. Use and specify your own codes; if you use ICD codes specify revision, eg ICD-9 or ICD-10)*
39. **Date of this second malignancy**
40. **MIGHT this have been a breast cancer metastasis?** *(1=no, 2=possibly/not yet certain [eg, possible lung, liver, bone or brain metastasis: please do not report definite breast metastases as second cancers])*

H) SURVIVAL (Q41-43)

41. **Is patient known to have died?** *(1=no, 2=yes)*
42. **<u>If no:</u> Date patient last known to be alive; <u>If yes:</u> Date of death**
43. **<u>If yes:</u> Cause of death** *(use and specify your own codes; if you use ICD codes specify which version, eg ICD-9 or ICD-10)*

ADDITIONAL VARIABLES (Q44-54)

I) ADDITIONAL TUMOUR MARKER DATA (Q44-52; OR, USE YOUR OWN CODES)
Note: If tests of gene expression or special tests of IHC quantitation were done on the excised primary then please send a separate file in your own format with the fully detailed set of results on each individual.

44. Summary of gene-expression status of primary tumour *(1=low risk, 2=intermediate risk, 3=high risk):* NB Please also provide the fully detailed gene expression results for each patient as a separate dataset.
45. Quantitative gene-expression prognostic score *(best available single numerical measure)*
46. Prognostic score used to quantify gene expression profile *(use own code, or: 1=OncotypeDx prognostic score, 2=Mammaprint prognostic score, 9=other [please specify])*

47. Summary of Topo-isomerase II alpha (TOPO2A) status of primary tumour *(1= normal [ie, no gene over-expression or deletion], 2=positive/over-expressing, 3=deleted)*
48. Quantitative TOPO2A measurement *(done in central/reference laboratory if possible)*
49. Units for TOPO2A *(1=IHC [% staining], 2=IHC score [0, 1+, 2+, 3+], 3=FISH [number of copies], 4=FISH [TOPO:CEP17 ratio], 5=CISH [# copies], 6=CISH [TOPO:CEP17], 9=other [please specify])*

50. Summary of Proliferation Index of primary tumour *(1=low, 2=intermediate, 3=high)*
51. Quantitative Proliferation Index *(best available single numerical measure, in central/ ref lab if possible)*
52. Factor measured for Proliferation Index *(1=S-phase fraction [%], 2=thymidine labelling index [%],
3=Ki-67 by IHC [% staining], 9=other [please specify]*)

J) BONE FRACTURES AND CARDIOVASCULAR EVENTS (Q53-54; OMIT IF NOT SOUGHT)
Some trial treatments may cause or prevent bone fractures or cardiovascular events. Please describe all such events (eg, hip fracture, spinal fracture, myocardial infarction, stroke, pulmonary embolus, episode of cardiac failure) if, but only if, such events were sought and recorded systematically for the trial.

Note: If tests of bone density or cardiac ejection fraction (LVEF, %) were done systematically on all patients then please send a separate file in your own format with all test results (and their dates) on each individual.

If more than one bone fracture or cardiovascular event was recorded, repeat variables 53-54 for each.

53. Nature of event *(use your own codes; if you use ICD codes, specify which version, eg ICD-9 or ICD-10, and if you use CTC Adverse Event codes, please specify version number, eg CTCAE-3 or CTCAE-4)*
54. Date of event
